# Rapid divergence of a gamete recognition gene promoted macroevolution of Eutheria

**DOI:** 10.1186/s13059-022-02721-y

**Published:** 2022-07-11

**Authors:** Emma K. Roberts, Steve Tardif, Emily A. Wright, Roy N. Platt, Robert D. Bradley, Daniel M. Hardy

**Affiliations:** 1 Department of Biological Sciences, Texas Tech University, Lubbock, TX USA; 2 Department of Cell Biology and Biochemistry, Texas Tech University Health Sciences Center, Lubbock, TX USA; 3 Reproductive Biology Division, JangoBio, Fitchburg, WI USA; 4 Host-Pathogen Interaction Program, Texas Biomedical Research Institute, San Antonio, TX USA; 5 Natural Science Research Laboratory, Museum of Texas Tech University, Lubbock, TX USA

**Keywords:** Bioinformatics, Fertilization, Gamete recognition, Mammals, Molecular evolution, Phylogenetics, Reproductive isolation, Speciation, Species specificity, Zonadhesin

## Abstract

**Background:**

Speciation genes contribute disproportionately to species divergence, but few examples exist, especially in vertebrates. Here we test whether *Zan*, which encodes the sperm acrosomal protein zonadhesin that mediates species-specific adhesion to the egg’s zona pellucida, is a speciation gene in placental mammals.

**Results:**

Genomic ontogeny reveals that *Zan* arose by repurposing of a stem vertebrate gene that was lost in multiple lineages but retained in Eutheria on acquiring a function in egg recognition. A 112-species *Zan* sequence phylogeny, representing 17 of 19 placental Orders, resolves all species into monophyletic groups corresponding to recognized Orders and Suborders, with <5% unsupported nodes. Three other rapidly evolving germ cell genes (*Adam2*, *Zp2*, and *Prm1*), a paralogous somatic cell gene (*TectA*), and a mitochondrial gene commonly used for phylogenetic analyses (*Cytb*) all yield trees with poorer resolution than the *Zan* tree and inferior topologies relative to a widely accepted mammalian supertree. *Zan* divergence by intense positive selection produces dramatic species differences in the protein’s properties, with ordinal divergence rates generally reflecting species richness of placental Orders consistent with expectations for a speciation gene that acts across a wide range of taxa. Furthermore, *Zan*’s combined phylogenetic utility and divergence exceeds those of all other genes known to have evolved in Eutheria by positive selection, including the only other mammalian speciation gene, *Prdm9*.

**Conclusions:**

Species-specific egg recognition conferred by *Zan*’s functional divergence served as a mode of prezygotic reproductive isolation that promoted the extraordinary adaptive radiation and success of Eutheria.

**Supplementary Information:**

The online version contains supplementary material available at 10.1186/s13059-022-02721-y.

## Background

Eighty years since Dobzhansky and Mayr unified Darwinian evolution and Mendelian genetics in the Modern Synthesis [1, 2], we still seek full understanding of speciation’s genetic basis [3–7]. In its simplest conception, speciation may be viewed as an inevitable consequence of prolonged microevolution, with species differences arising entirely by gradual accumulation of mostly neutral genetic changes over a long period of time, ultimately producing genetically distinct populations (“phyletic gradualism” [8–12];). Consistent with that view, robust phylogenetic supertrees constructed in part by comparing thousands of neutrally evolving gene sequences provide strong insight into timing and order of speciation events [10, 13–19]. However, any tenable theory of speciation genetics must also account for adaptive changes that enable evolving species to exploit new “opportunities for living” [7, 20–22]. Such adaptive evolution can occur rapidly, between periods of relative stasis (“punctuated equilibria” [9];), driven in part by action of positive selection for functional differences in gene products that contribute to the fitness and success of nascent species [23–25].

The idea that adaptive molecular evolution may drive speciation prompted searches for “speciation genes,” defined as genes that contribute disproportionately to species divergence [4, 22, 26, 27]. Speciation genes have proven difficult to identify; indeed, in the absence of a universally accepted definition of species, the definition of a speciation gene is necessarily subject to interpretation [28–32]. In animals, fewer than 10 speciation genes have been explicitly identified, mostly in *Drosophila* species, according to strict criteria [3, 22, 29, 30, 33–40]. In contrast, more than 40 plant speciation genes have been described [26, 30, 41, 42]. The greater number in plants derives in part from the pervasive facilitation of speciation by polyploidy, especially in crop species, which helps sustain fertility of hybrids, and in part because any gene that contributes to reproductive isolation, whether a complete or even a partial barrier, is considered a speciation gene [26, 41, 43–46]. Notwithstanding differences in underlying genetic processes and lack of strict consensus on criteria for their identification, some generalizations about speciation genes can be made: (1) few have been identified either in plants or animals, despite great interest in their discovery; (2) most if not all have only been shown to promote divergence of closely related pairs or small numbers of species [29, 30, 39, 47]; (3) selection for species-divergent function drives their rapid evolution [24, 48]; and (4) their products’ activities are known or at least suspected to contribute to reproductive isolation [3, 29, 30, 35, 38, 49–51].

Reproductive isolation promotes speciation by limiting homogenizing gene flow between incipient species as they diverge to become independent genetic entities [29, 52–54]. Genetic changes that promote reproductive isolation serve not only to reinforce speciation secondary to geographic isolation, but also to initiate and drive speciation in populations with overlapping or identical ranges or niches [55]. In vertebrates, modes of reproductive isolation vary and include prezygotic barriers such as mate discrimination, anatomical incompatibility, and fertilization specificity, as well as postzygotic barriers such as embryo inviability and hybrid sterility [25, 55–57]. Partly reflecting a paucity of information on prezygotic barriers, most known speciation genes promote postzygotic reproductive isolation, primarily hybrid inviability or subfertility, especially in animals [3, 4, 12, 29, 38, 49, 58, 59].

In animal species ranging from marine invertebrates to placental mammals, unique pairs of sperm-egg adhesion molecules mediate species-specific gamete recognition that serves as a barrier to interspecific fertilization, and thereby likely contributes to prezygotic reproductive isolation [60–63]. In molluscs and echinoderms, species-specific sperm-egg recognition prevents formation of hybrid offspring during spawning of species with overlapping ranges [25, 64, 65]. The active recognition molecule pairs, sperm lysin and its egg counterpart VERL (Vitelline Envelope Receptor for Lysin) in molluscs [66–68], and sperm bindin and its egg counterpart EBR (Egg Bindin Receptor) in echinoderms [69, 70], acquired their species-specific binding activities through combined action of positive selection and concerted evolution [24, 25, 71]. Thus, in these externally fertilizing organisms, rapid molecular evolution of gamete recognition proteins confers fertilization specificity that serves as a primary mode of reproductive isolation [22]. Despite the obvious implication that these well-characterized pairs of gamete recognition molecules promote speciation in molluscs and echinoderms, their corresponding genes are not generally recognized as speciation genes, in part because a lack of genome data precludes robust phylogenetic analyses. In addition, strict criteria typically applied in animals favor identification of genes or loci that confer absolute barriers most amenable to experimental analysis, especially postzygotic barriers such as hybrid inviability or sterility, between closely related pairs or small numbers of species [3, 29, 30, 35, 38, 39, 49, 51, 57–59], rather than partial barriers such as gamete incompatibility that may act broadly in taxa. Analogous to mollusc lysin and echinoderm bindin, in mammals the rapidly evolving sperm protein zonadhesin (gene: *Zan*) mediates species-specific adhesion to the egg’s zona pellucida (ZP) [62, 72–74]. No studies have yet determined the extent to which fertilization barriers in any vertebrate species contribute to reproductive isolation (i.e., quantified the “effect size” of the barrier [30]), or for that matter shown unequivocally that such barriers are even relevant in animals such as mammals that fertilize internally. Nevertheless, given the established functions of lysin, VERL, bindin, EBR, and zonadhesin as mediators of species-specific gamete recognition, their corresponding genes may be speciation genes that act by promoting post-mating, prezygotic reproductive isolation [75, 76].

Species divergence of any gene can potentially serve as a clock to measure time passed since a speciation event [77, 78]. However, gene divergence reflects not only passage of time but also evolution of gene product functions, with negative selection acting to preserve function and positive selection acting to bestow beneficial new traits in the evolving organisms [79]. Indeed, because evolution of a gene product is likely to reflect selection-driven conservation or divergence of its function rather than overall species divergence, supertree phylogenies typically omit genes subject to selection, and instead include large numbers of neutrally evolving genes [10, 18]. Nevertheless, a speciation gene that acts more broadly than to promote divergence of a few closely related species should (1) evolve in strict concordance with species phylogeny and divergence rate; (2) exhibit signatures of positive selection for species-divergent function; and (3) plausibly contribute to reproductive isolation. To investigate possible genetic contributions to post-mating, prezygotic barriers that may promote speciation among placental mammals, here we tested the hypothesis that *Zan* is a speciation gene using a combination of genome ontogeny, gene tree phylogeny with selection analysis, and biochemical approaches. Specifically, we characterized *Zan*’s molecular evolution and phylogenetic utility in comparison to all Eutherian genes previously shown to have evolved under positive selection, with detailed analyses of four gamete-specific, germ cell genes (*Zan*, *Adam2*, *Zp2*, and *Prm1*), a somatic paralog of *Zan* (*Tecta*), and a mitochondrial gene (*Cytb*), among more than 100 species representing 17 of 19 Eutherian Orders.

## Results

### Zan ontogeny

**Fig. 1:**
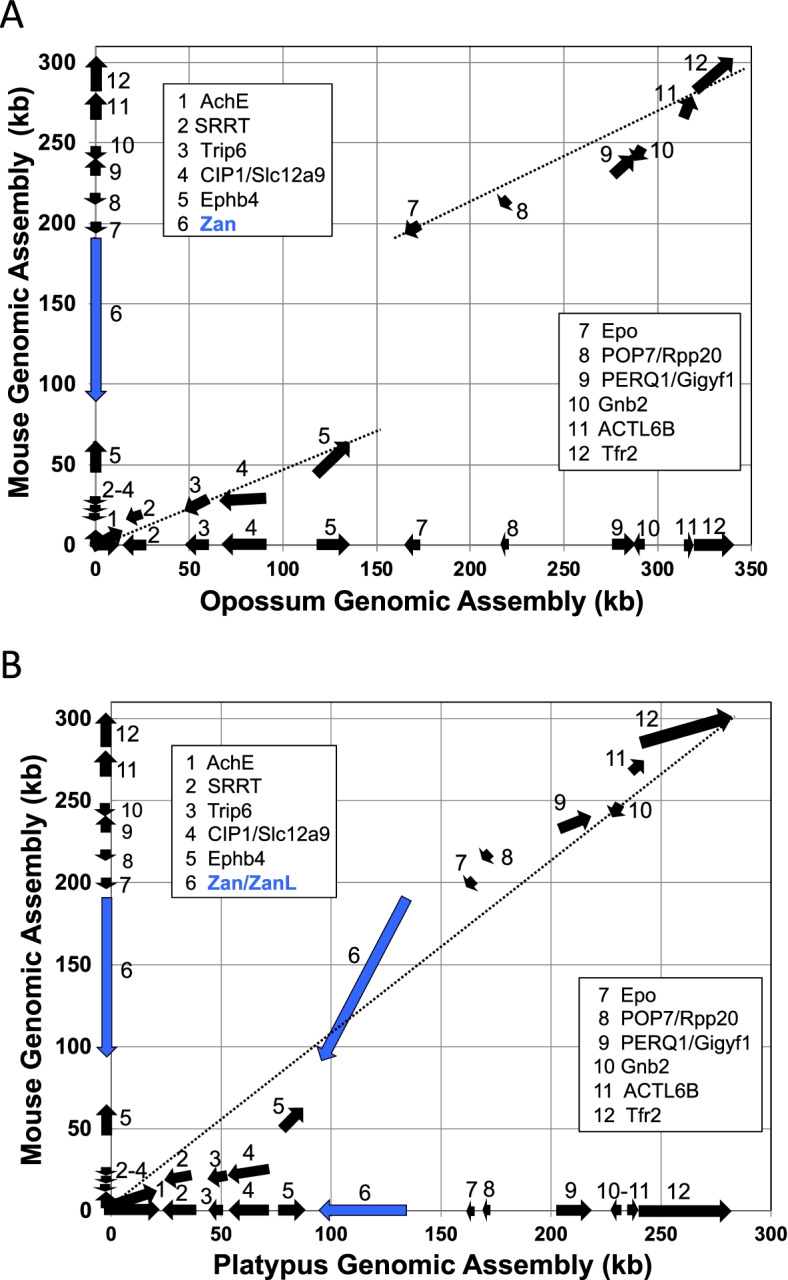
Two-dimensional comparison of mouse, opossum, and platypus genomic loci spanning *AchE*-*Tfr2.***A** Comparison of syntenic loci from mouse Chr 5 (~310 kb) encompassing 12 identified genes and opossum Chr 2 (~340 kb) encompassing 11 of the same 12 genes. The arrows represent the locations and orientations of the respective genes (first through last exons plus introns; blue arrow = mouse *Zan*). Note the co-linearity and conserved order and orientation of genes 1–5 (*AchE*–*Ephb4*) and 7–12 (*Epo*–*Tfr2*), and the generally greater content of intergenic and intronic DNA in the opossum. Note also the comparatively short segment of intergenic DNA (30 kb) between opossum *Ephb4* and *Epo*, and the corresponding discontinuity in the co-linear relationship (dotted lines) between the mouse and opossum loci, reflecting the absence of ~100 kb *Zan* in the opossum. **B** Corresponding comparison of the mouse *Zan* locus with the syntenic locus from platypus Chr X5 (~270 kb). Note the presence of a *Zan*-like gene (*ZanL*) in platypus between *Ephb4* and *Epo*

Rapid gene divergence presents difficulties for distinguishing between paralogs and distant orthologs. Accordingly, to identify *Zan* orthologs, we initially retrieved candidate sequences by querying NCBI and Ensembl databases, as well as raw sequence from several non-Eutherian species, by a combination of word, TBLASTX, and BLASTp searches. NCBI Protein database queries retrieved >1200 entries annotated as “zonadhesin,” including sequences from viruses, bacteria, protists, fungi, and plants. Animals accounted for the vast majority of entries (>1000), with species ranging broadly from cnidarians and nematodes to fishes, reptiles, birds, and mammals. However, entries from most species other than Eutherian mammals differed markedly in predicted gene product size, sequence, and domain composition as compared to prototypical *Zan* gene products in species such as pig, mouse, and human that have been directly characterized, suggesting those entries were not truly *Zan*. We therefore set two criteria to identify authentic *Zan* in genome assemblies: (1) predicted protein domain composition to include, in order, MAM (meprin/A5 antigen/receptor protein tyrosine phosphatase *mu*), mucin, and tandem VWD (von Willebrand factor type-D) domains [73], and (2) fully shared synteny among species, evident as conserved gene content, order, and orientation between *Ephb4* and *Epo* in the genomic locus spanning *AchE* to *Tfr2* [80]. No non-Eutherian genes annotated as *Zan* met these criteria. Indeed, two-dimensional comparison of the mouse (*Mus musculus*) *Zan* genomic locus spanning *AchE*–*Tfr2* with the corresponding opossum (*Monodelphis domestica*) locus revealed a marked discontinuity between the *Ephb4* and *Epo* genes flanking *Zan* (Fig. 1A) owing to the absence of *Zan* in opossum despite conservation and shared synteny of the other eleven genes. The full opossum assembly was approximately 30 kb longer than the mouse assembly (340 vs. 310 kb), reflecting a generally greater content of non-coding intergenic and intronic DNA in the opossum locus. Nevertheless, the *Ephb4*–*Epo* intergenic segment spanned only 30 kb, which is too short to accommodate 100+ kb *Zan*, and local TBLASTX search of the 30 kb with mouse *Zan* detected no *Zan*-like sequences. Surprisingly, even though monotremes (Prototheria) diverged basal to Metatheria and Eutheria (estimated at 166 vs. 148 Myr ago, respectively [15];), two-dimensional comparison of mouse and platypus (*Ornithorhynchus anatinus*) *AchE*–*Tfr2* syntenic loci (Fig. 1B) revealed presence of a platypus *Zan*-like gene (hereafter designated *ZanL*) encoding a protein with mucin and tandem VWD domains but incongruous predicted domain content (no MAM domains and double the number of full VWD domains) in comparison to authentic *Zan*.

In searches for *Zan* loci that may have eluded previous annotation efforts, TBLASTX query with 112 Eutherian *Zan* DNA sequences retrieved no matching *Zan* sequences from raw genomic reads of six non-Eutherian species, including one amphibian (*Xenopus laevis*), one bird (*Gallus gallus*), and three marsupials (*M. domestica*, *Sarcophilus harrisii*, and *Notamacropus eugenii*). Similar query of the same six sets of genomic reads with aligned DNA sequences encoding ADAM 3, another rapidly evolving sperm-specific protein that functions in fertilization [81, 82], retrieved multiple ADAM sequences from each organism, suggesting that this search strategy would have retrieved *Zan* had it been present in the genomes of the queried species.

Despite the apparent absence of *Zan* in marsupials, birds, and amphibians, BLASTp search with armadillo (a basal Eutherian mammal from Superorder Xenarthra) zonadhesin protein sequence retrieved not only 100+ mammalian sequences as expected, but also zonadhesin-like predicted sequences in three reptiles (Chinese soft-shelled turtle, *Pelodiscus sinensis*; painted turtle, *Chrysemys picta*; Chinese alligator, *Alligator sinensis*), three ray-finned fish (large yellow croaker, *Larimichthys crocea*; pufferfish, *Takifugu rubripides*; zebrafish, *Danio reria*), and one lobe-finned fish (coelocanth, *Latimeria chalumnae*) (Additional file 1: Table S1). Similar to the zonadhesin-like protein encoded by the *ZanL* gene identified in platypus, the reptile and fish proteins differed from zonadhesin in predicted size, domain composition, and domain arrangement. To determine the relationship of the corresponding reptile and fish genes to *Zan*, we evaluated the *Alligator*, *Pelodiscus*, *Latimeria*, *Takifugu*, and *Danio* genomic loci for evidence of shared synteny with the Eutherian *Zan* locus. The *Alligator* locus comprised, in part, eight (*Actl6B*, *Srrt*, *EphB4*, *Tfr2*, *Epo*, *Pop7/Rpp20*, *GigyF1*, and *AchE*) of the 11 genes flanking *Zan* in the Eutherian syntenic region spanning *AchE–Tfr2*, but without conserved gene order and orientation. Similarly, the *Pelodiscus* locus comprised in part five genes (*Srrt*, *EphB4*, *Gnb2*, *Actl6B*, and *GigyF1*), and the *Latimeria* locus five other genes (*Tfr2*, *SLC12A9*, *Pop7/Rpp20*, *Epo*, and *GigyF1*) present in the Eutherian *Zan* syntenic region, also without conserved order and orientation. Finally, in *Danio* and *Takifugu*, the 10 nearest genes on either side of the genes encoding zonadhesin-like proteins included only *AchE* from the *Zan* syntenic region, and *Serpine1*, which in human is 230 kb distal to *AchE* outside the *Zan* syntenic region. In sum, shared synteny among the loci decreased progressively with increasing evolutionary distance, suggesting that, like *Ornithorhynchus ZanL*, the *Alligator*, *Pelodiscus*, *Latimeria*, *Takifugu*, and *Danio* genes are also *ZanL* genes descended from an ancient *Zan/ZanL* progenitor that has been retained in fish but lost in multiple tetrapod lineages.

Altogether, we identified *Zan* in 112 species representing 17 of 19 placental Orders, and *ZanL* genes in one monotreme and five non-mammalian vertebrates, but found no *Zan* or *ZanL* genes in marsupials, birds, or amphibians. Thus, authentic *Zan* appeared only in genomes of Eutherian mammals.

### Zan phylogeny

**Fig. 2:**
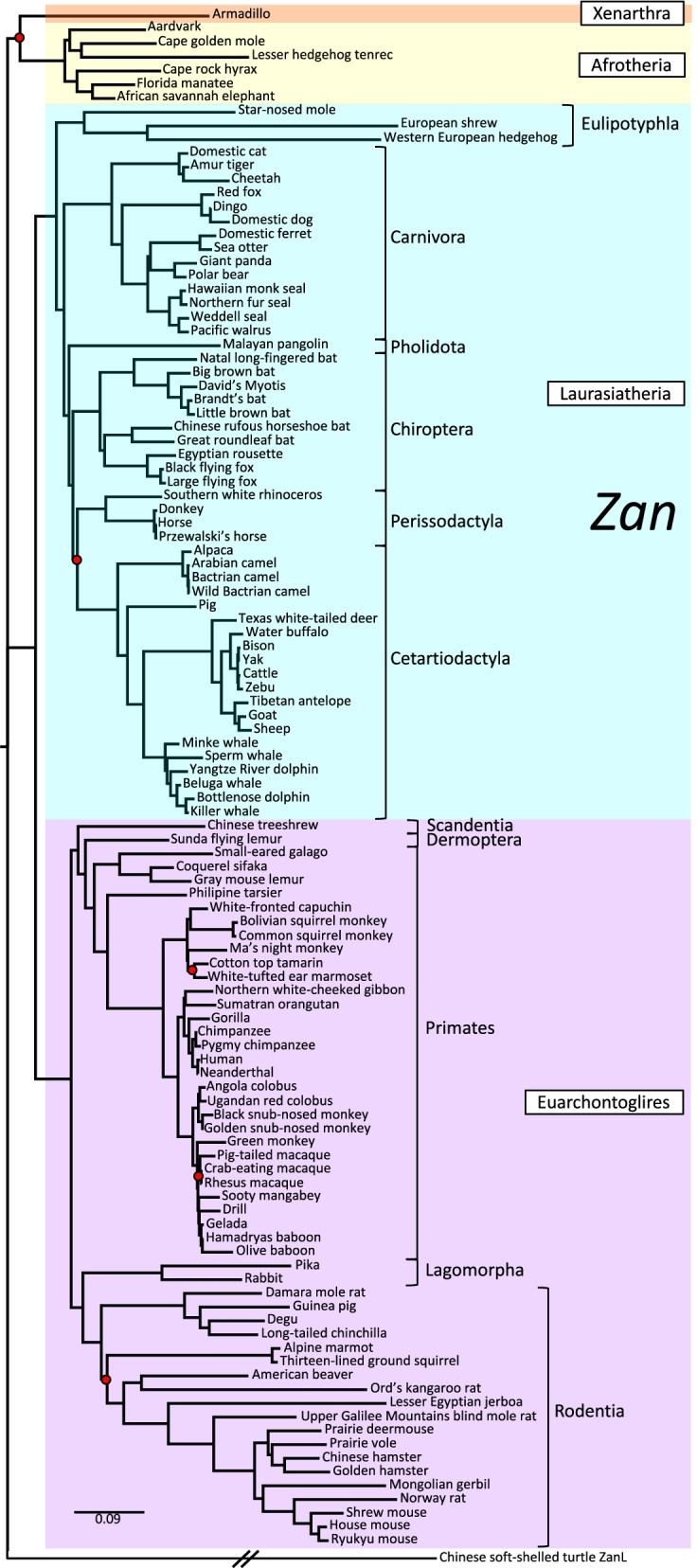
Phylogenetic analysis of *Zan* DNA sequence divergence. Shown is the gene tree constructed by Bayesian analysis of 112 aligned *Zan* sequences, with *ZanL* from Chinese soft-shelled turtle as outgroup. Red dots denote nodes lacking statistical support (5/112 nodes = 4.5%). Note the supported grouping of cetaceans with artiodactyls, as well as the monophyletic grouping of all species into their respective Orders and of all Orders into their respective Superorders. Taxonomy per [84]

To compare *Zan* among the 112 placental species, we first characterized the genes’ exon and encoded domain structures. Zonadhesin’s ZP-binding activity resides in its D0-D4 VWD domains [72, 73]*.* However, human *ZAN* comprises 48 exons, whereas mouse *Zan* comprises 88 exons owing to presence in the mouse protein of an additional 20 partial VWD domains between D3 and D4, designated D3p domains, each encoded by a two-exon cassette [80, 83]. We found D3p domain expansions only in the 10 species from rodent Superfamily Muroidea among the 11 species from Suborder Myomorpha, with the number of domains ranging from zero in Lesser Egyptian jerboa (*Jaculus jaculus*, the only myomorph species in our analysis from Superfamily Dipodoidea) to 24 in North American deermouse (*Peromyscus maniculatus*, one of the 10 myomorph species from Muroidea in our analysis). Therefore, to compare orthologous *Zan* sequences across all placental Orders, we removed D3p domain coding regions from the predicted *Zan* mRNA sequences of the 10 muroid rodent species, then aligned sequences encoding D0–D4, with corresponding tandem VWD domains of Chinese soft-shelled turtle (*P. sinensis*) *ZanL* as outgroup. Bayesian analysis of the alignment (GTR+Γ+I nucleotide substitution model; Additional file 1: Tables S2A-S2B) produced a phylogenetic tree (Fig. 2) that corresponded closely with Eutherian phylogenies constructed from other morphometric and molecular data [15, 18, 85–87], with posterior support (*Ρ* ≥ 0.95) at 107 of 112 nodes.

### Phylogeny comparisons

**Fig. 3:**
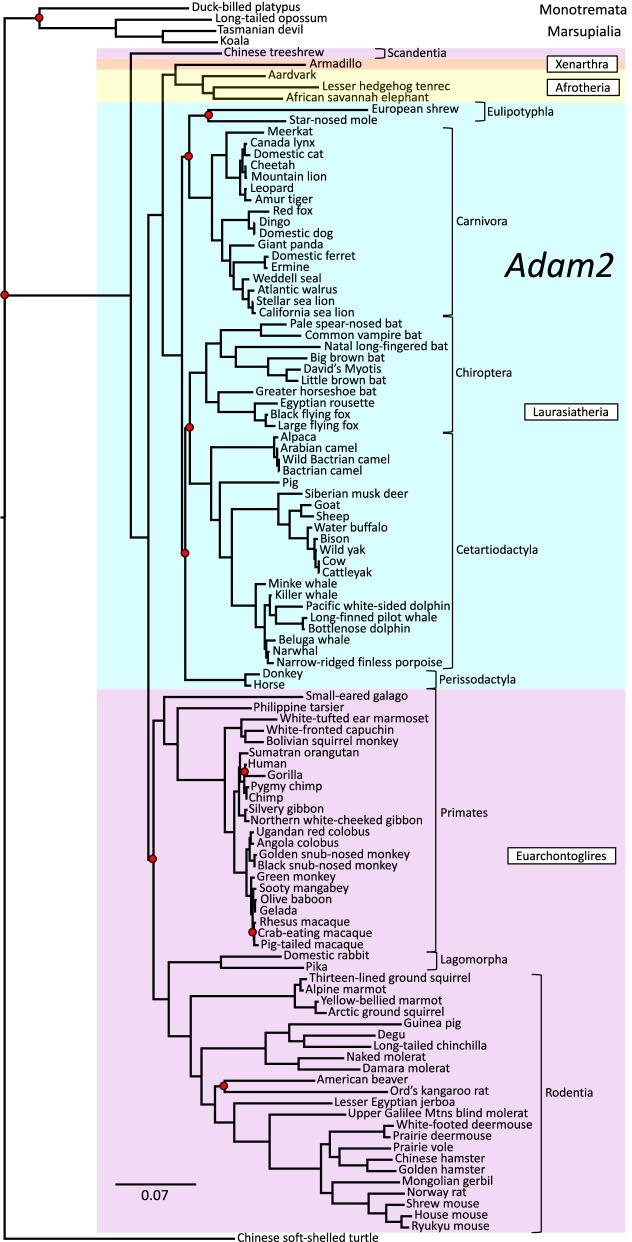
Phylogenetic analysis of *Adam2* DNA sequence divergence. Shown is the gene tree constructed by Bayesian analysis of 110 aligned *Adam2* sequences, with Chinese soft-shelled turtle as outgroup. Red dots denote nodes lacking statistical support (10/109 = 9.2%). Note the supported but aberrant grouping of Order Scandentia basal to Superorders Xenarthra, Afrotheria, and Laurasiatheria rather than basal to Order Primates within Superorder Euarchontoglires

**Fig. 4:**
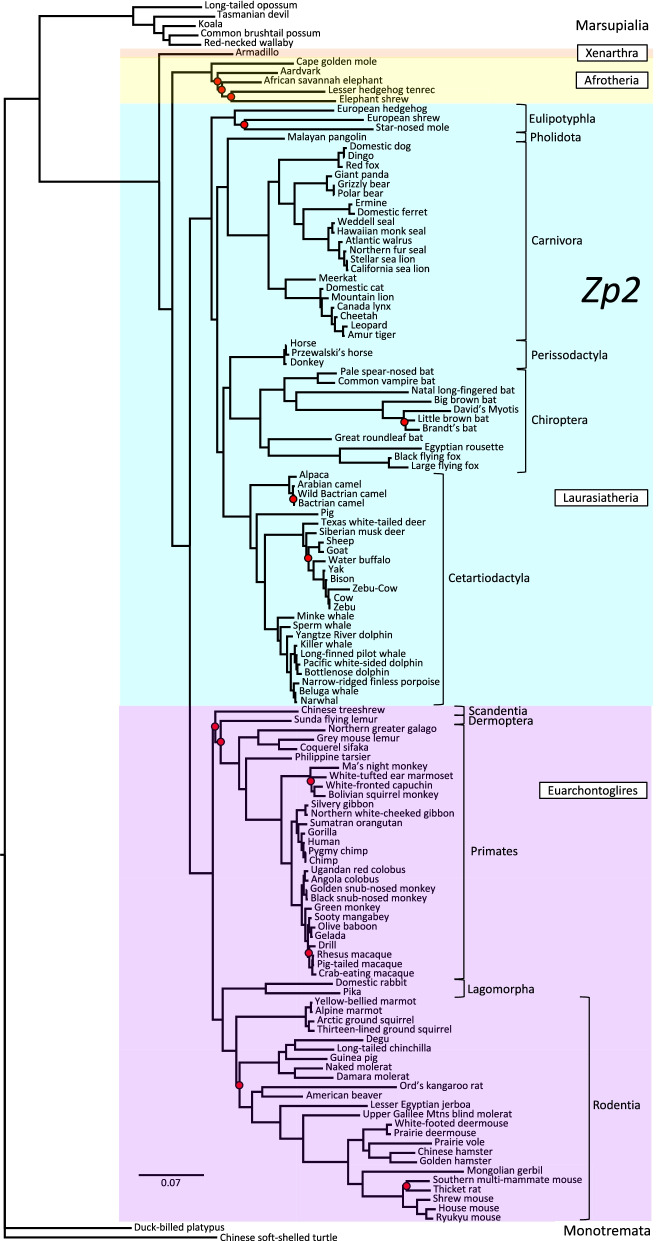
Phylogenetic analysis of *Zp2* DNA sequence divergence. Shown is the gene tree constructed by Bayesian analysis of 132 aligned *Zp2* sequences, with Chinese soft-shelled turtle as outgroup. Red dots denote nodes lacking statistical support (13/131 = 9.9%). Note the supported grouping of Order Perissodactyla with Chiroptera rather than with Cetartiodactyla as in the *Zan* tree, consistent with ongoing controversy in its phylogenetic placement [88–90]

**Fig. 5:**
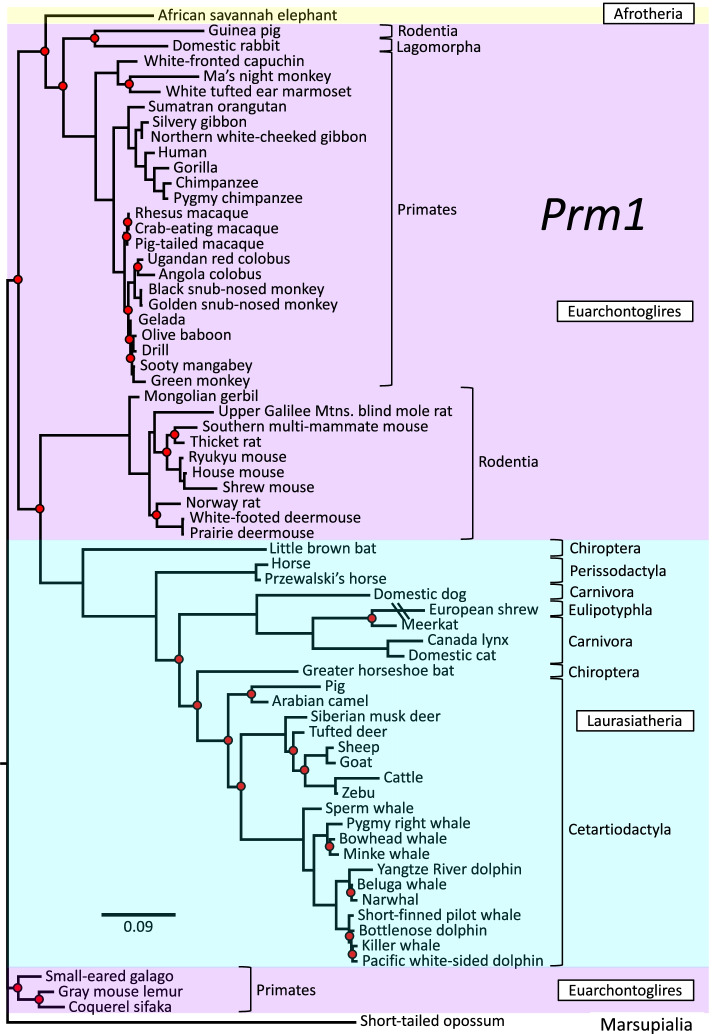
Phylogenetic analysis of *Prm1* DNA sequence divergence. Shown is the gene tree constructed by Bayesian analysis of 67 aligned *Prm1* sequences, with short-tailed opossum as outgroup. Red dots denote nodes lacking statistical support (29/66 nodes = 44%). Note the supported absence of monophyly for Order Primates within Superorder Euarchontoglires and the supported but aberrant placement of the Sumatran orangutan basal to gibbons in the apes clade

**Fig. 6:**
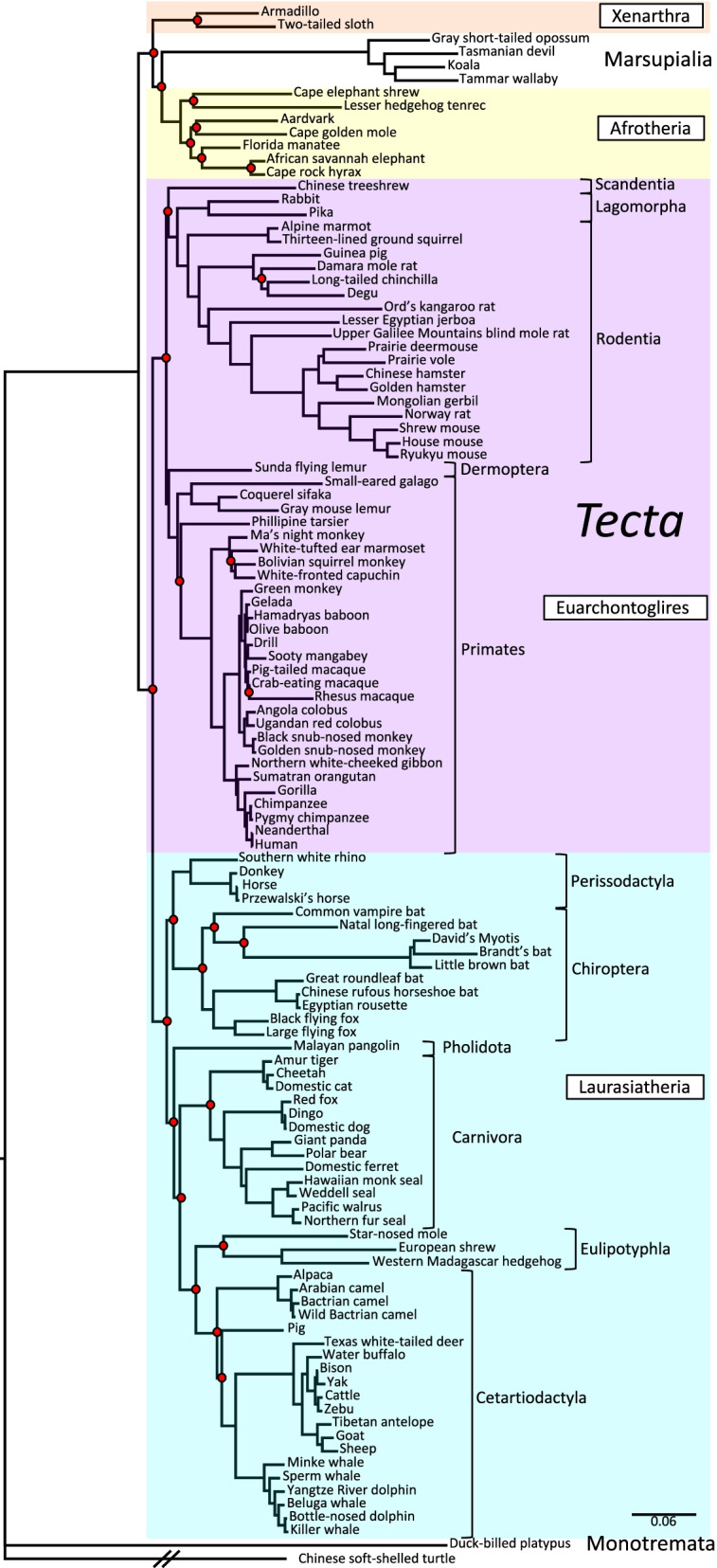
Phylogenetic analysis of *Tecta* DNA sequence divergence. Shown is the gene tree constructed by Bayesian analysis of 116 aligned *Tecta* sequences, with Chinese soft-shelled turtle as outgroup. Red dots denote nodes lacking statistical support (27/115 = 23% unsupported). Note the discrepant grouping of Infraclass Marsupialia with Superorders Xenarthra and Afrotheria of placental mammals, rather than basal to all Eutheria. Note also the prevalent lack of support in Afrotheria and at deeper nodes in Superorder Laurasiatheria

**Fig. 7:**
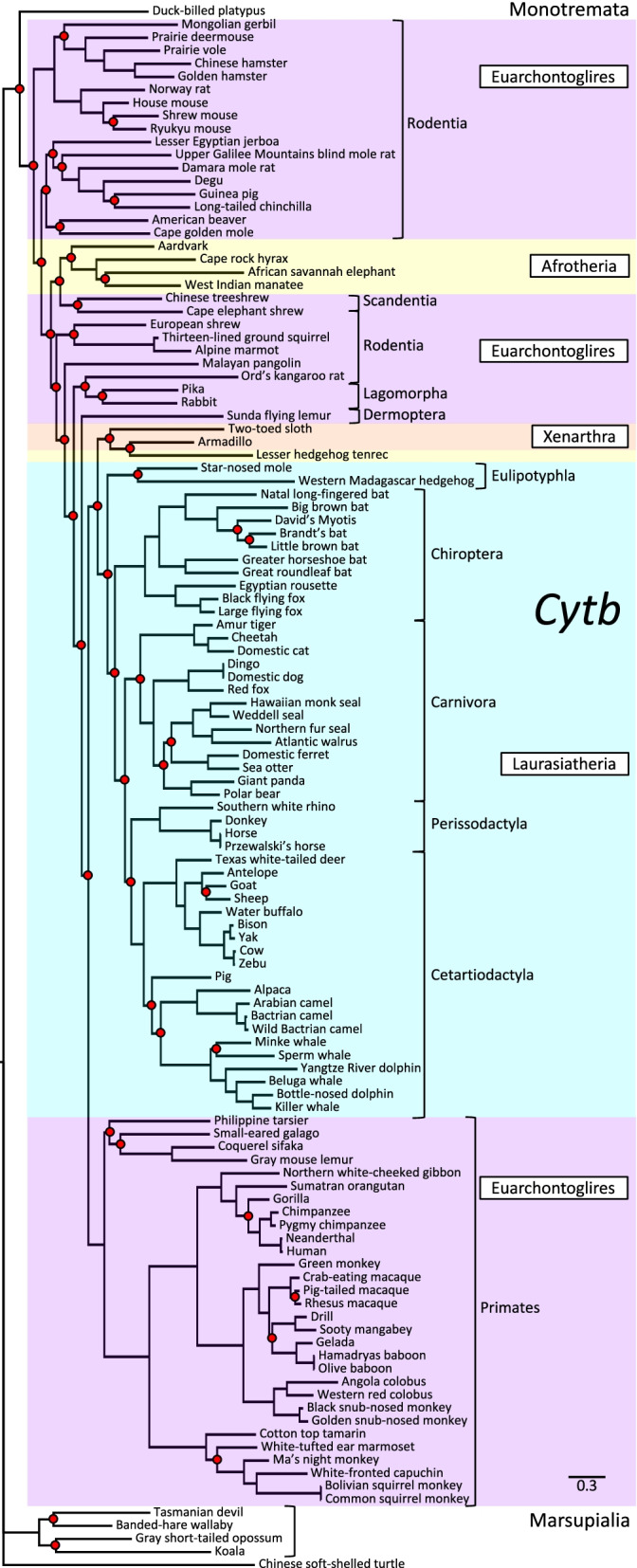
Phylogenetic analysis of *Cytb* DNA sequence divergence. Shown is the gene tree constructed by Bayesian analysis of 119 aligned *Cytb* sequences, with Chinese soft-shelled turtle as outgroup. Red dots denote nodes lacking statistical support (47/118 = 39.8% unsupported). Note the many discrepant groupings, including absence of monophyly for Superorders Euarchontoglires and Afrotheria, grouping of Superorder Xenarthra with Superorder Laurasiatheria rather than branching basal to all other Eutherian Superorders, and grouping of mammalian Infraclasses Monotremata with Eutheria rather than basal to Marsupialia

In contrast to the highly resolved *Zan* phylogeny, similar analyses of five other genes (*Adam2*, *Zp2*, *Prm1*, *Tecta*, and *Cytb*) yielded trees with many more unsupported nodes and with discrepant grouping of species and Orders (Figs. 3, 4, 5, 6, and 7). Among these comparison genes, *Adam2* and *Zp2* are plausible, candidate speciation genes owing to their suspected or established functions in mammalian sperm-egg recognition. Specifically, *Adam2* encodes ADAM 2 (a disintegrin and metallopeptidase domain 2), a rapidly evolving, sperm membrane-specific protein with a yet-unclear function in sperm-egg interaction [91, 92], and *Zp2* encodes ZP2, an egg-specific zona pellucida glycoprotein that mediates sperm adhesion during fertilization [24, 63, 93, 94]. A third germ cell-specific comparison gene, *Prm1*, has served extensively as a model for rapid evolution of reproductive genes, and encodes protamine 1, which replaces histones in chromatin condensation during spermatogenesis [95–99]. For a somatic, nuclear comparison gene, we chose *Tecta*, which encodes α-tectorin, a tectorial membrane protein comprising tandem VWD domains paralogous to zonadhesin D0-D4 [100–102]. And finally, for a rapidly evolving mitochondrial gene, we chose *Cytb*, which is commonly used as a marker for molecular phylogeny and encodes the electron transport chain protein cytochrome b [11, 86, 103]. The *Adam2*, *Zp2*, *Tecta*, and *Cytb* trees (Figs. 3, 4, 6, and 7, respectively) each included more than 100 species similar to those in the *Zan* tree, but the *Prm1* tree (Fig. 5) included only 67 species because no *Prm1* sequence was available in genomic databases for many of the species represented in the *Zan* tree. Fraction of nodes lacking posterior support for these comparison genes ranged from 9.2 to 44% (*Adam2*, 10/109 = 9.2%; *Zp2*, 13/131 = 9.9%; *Tecta*, 27/115 = 23%; *Cytb*, 47/119 = 40%; *Prm1*, 29/66 = 44%), as opposed to only 4.5% unsupported nodes in the *Zan* tree. The *Zp2* tree, like the *Zan* tree, largely reflected established phylogenetic relationships, whereas the other four genes each exhibited discrepant taxonomic groupings evident as superordinal polytomies (Figs. 3, 4, 5, 6, and 7).

To determine if *Zan* gene phylogeny accurately reflected Eutherian species phylogeny, we compared the *Zan* tree topology to that of a widely accepted species supertree constructed from extensive gene sequence and morphometric data [15] that nevertheless contains many polytomies [104]. Parsimony analysis (PAUP* v4.0a166; ref. [105]) yielded only three candidate *Zan* trees, each about equally likely, that largely recapitulated the established supertree phylogeny. Shimodaira-Hasegawa (SH) and Shimodaira Approximately Unbiased (AU) tests (α values all Bonferroni-corrected for *Zan* and all subsequent genes’ comparisons) revealed that the global topology of the best (i.e., most likely) *Zan* tree differed (*Ρ* <0.0001, both tests) from a single best candidate tree constrained to the topology of the established supertree, because the *Zan* tree is more fully resolved (Additional file 1: Table S3). Parsimony analysis also yielded single best *Zan* topologies for Superorder Afrotheria and the five Orders with four or more species represented (Carnivora, Cetartiodactyla, Chiroptera, Primates, Rodentia; Additional file 1: Table S4). Ordinal *Zan* topologies did not differ (AU test) from their respective supertree topologies for Orders in which the supertree is well resolved (Cetartiodactyla, *Ρ* = 0.5; Primates, *Ρ* = 0.21), but did differ for Superorder Afrotheria (*Ρ* > 0.0001), as well as for Orders with polytomies in the supertree (Carnivora, *Ρ* = 0.01; Chiroptera, *Ρ* < 0.0001; Rodentia, *Ρ* > 0.0001), consistent with the higher resolution of the *Zan* tree.

Detailed topological comparisons of *Adam2*, *Zp2*, *Prm1*, *Tecta*, and *Cytb* gene trees by parsimony analysis and SH and AU tests (Additional file 3) identified numerous ambiguities, including excessive numbers (*Adam2* and *Zp2*) and inferior unconstrained topologies (*Tecta*) of candidate trees, as well as gross incongruities with the supertree topology (*Prm1* and *Cytb*). Altogether, among the six genes examined, only *Zan* yielded a phylogenetic tree that was more highly resolved than and congruent with accepted relationships portrayed in a well-established Eutherian supertree [15].

### Divergence comparisons

To determine if *Zan* divergence subserves biologically relevant species differences in the zonadhesin precursor’s amino acid sequence, we globally evaluated the 112 species RNA sequence alignment for relative contribution of neutral evolution, negative selection, and positive selection (PAML test, ref. [106]). The analysis detected intense (*ω*_8_ (*dN/dS*) = 8.67) positive selection (PAML model M8; Additional file 1: Table S5) at 425 of 1932 sites (22.0%) in the compared *Zan* coding region (Bayes empirical Bayes posterior probability ≥ 0.95). In contrast, corresponding analyses of other reproductive genes each detected weaker positive selection (model M8; Additional file 1: Table S5) at a variable proportion of sites. Specifically, *Adam2* exhibited comparatively weak (*ω*_8_ = 2.34) and less pervasive (12.4% of sites) positive selection, *Zp2* also exhibited weak (*ω*_8_ = 2.13) but more pervasive (33.8% of sites) positive selection, and *Prm1* exhibited weaker (*ω*_8_ = 4.03) but similarly pervasive (22.7% of sites) positive selection.

For the somatic, non-reproductive genes, selection analysis of *Tecta* revealed equal likelihoods for the neutral evolution and positive/negative selection models (PAML M7 and M8, respectively; Additional file 1: Table S5) with, for model M8, overall weak (*ω*_8_ = 1.79) and relatively limited (7.7% sites) positive selection. Conversely, despite the more rapid divergence of the *Cytb* mitochondrial DNA sequences, *Cytb* exhibited no signatures of positive selection. Instead, PAML analysis of *Cytb* detected only neutral evolution (model M1, *ω*_1_ = 1.000) at a small proportion (6.4%) of sites, as well as intense (model M1, *ω*_0_ < 0.035) and extraordinarily pervasive (93.6% of sites) negative selection (Additional file 1: Table S5), consistent with the idea that species differences in *Cytb* serve primarily as a marker for time passed since species diverged [77] rather than adaptive changes in amino acid sequence associated with that divergence, owing to expected functional constraints on the evolution of an ancient and universally essential metabolic enzyme.

**Fig. 8:**
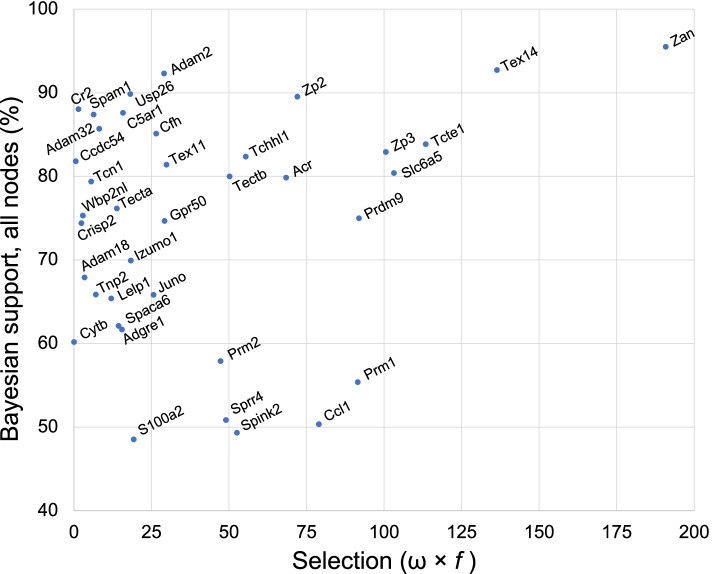
Relationship between phylogenetic support and selection intensity of all positively selected genes. Shown is a plot of support (percent of all Eutherian nodes with >95% posterior Bayesian support) vs. overall selection intensity [calculated as *ω* × *f* , the product of selection magnitude (*d*N/*d*S = *ω*) and pervasiveness (frequency = *f*), both assessed by Model M8]. Note the remarkably high support value (95.5%) and selection intensity (*ω* × *f* = 191) observed for *Zan* in comparison to the other genes, as well as the low support of several genes (*Prm1*, *Prm2*, *Ccl1*, *Spink2*, *Sprr4*) in the high end of the selection intensity range (*ω* × *f* = 45–100). We did not plot the points for the sensory/taste gene *Tas1r2* or the metabolic genes *Man2b1* and *Mgam* because they exhibited very low overall intensity of positive selection (*ω* × *f* < 4) similar to the data points for *Ccdc54*, *Crisp2*, and *Wbp2nl*

To assess relationships of positive selection to phylogenetic support more broadly, we retrieved all reliable sequences of all genes previously shown to have evolved by positive selection [24, 91, 95, 100, 107–122] and conducted Bayesian and selection analyses on individually constructed gene trees. Excluding 12 genes with too little taxonomic representation (either fewer than nine Orders total or no basal Orders), the analysis yielded Bayesian (>95% posterior support) and M7 and M8 model selection data (magnitude = *d*N/*d*S = *ω*, and frequency= *f*) for 40 positively selected genes with widely ranging functions, including 23 genes that function in reproduction, seven in sensory perception, five in immunity, three in metabolism, and one each in the nervous system and the cell cycle. We then plotted support (percent of all nodes) vs. a value reflecting overall intensity of positive selection (magnitude × frequency = *ω* × *f* ) calculated for each gene (Fig. 8 and Additional file 1: Table S5). The analysis included rapidly evolving but negatively selected (*ω* × *f* = 0) *Cytb* for reference. Bayesian posterior support levels ranged widely, from a low of 49% among all Eutherian nodes for the cell cycle gene *S100a2* to a high of 95.5% for *Zan*. Overall selection intensity ranged on the low end from less than nine for 11 genes, including six reproductive genes (*Adam18*, *Adam32*, *Ccdc54*, *Crisp2*, *Spam1*, *Tnp2*, and *Wbp2nl*), all three metabolic genes (*Man2b1*, *Mgam*, *Tcn1*), the immunity gene *Cr2*, and the sensory gene *Tas1r2*, up to a high value of 191 for *Zan*. Genes expressed exclusively in spermatozoa (*Tcte1*, *Tex14*, and *Zan*) or eggs (*Zp2* and *Zp3*) dominated the subset with highest combined phylogenetic support and overall intensity of positive selection. For many of the genes, resolution at terminal (within Family or Genus) nodes largely accounted for their support values, as support dropped substantially at deeper nodes, for example from 75 to 50% for *Prdm9*, from 60 to 38% for *Cytb*, from 70 to 54% for *Izumo1*, and from 62 to 39% for *Adgre2* (Roberts et al. unpublished). In contrast, deep node support in the *Zan* tree dropped by only 0.7%, from 95.5 to 94.8%.

**Fig. 9:**
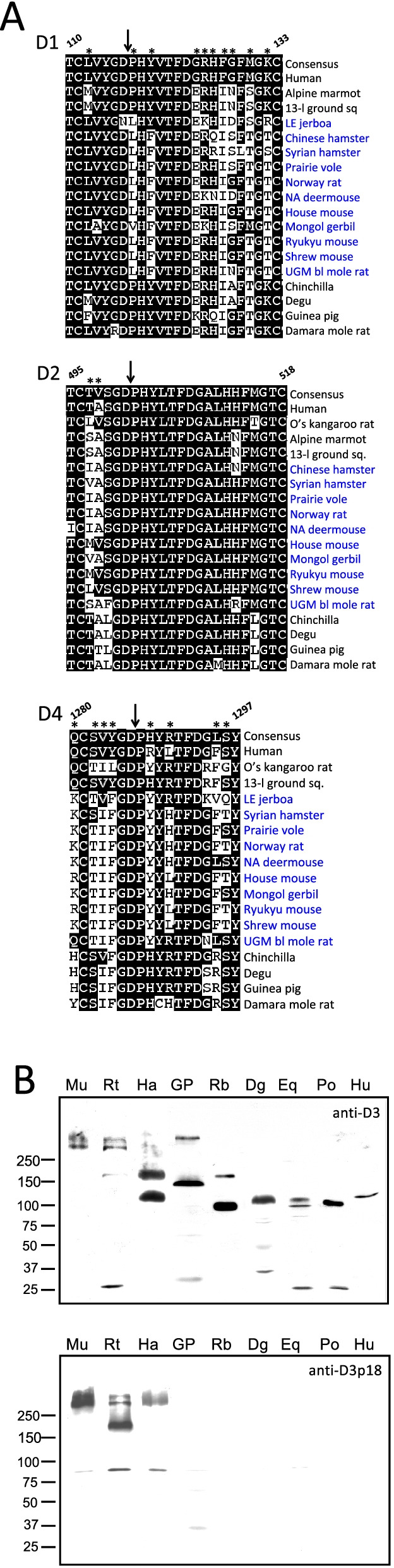
Species diversity of *Zan* amino acid sequence and processing. **A.** Taxon-specific variation in proteolytic processing sites of the zonadhesin precursor. Asp-Pro bonds (“DP,” downward arrows) cleaved during the proteolytic processing of the pig zonadhesin precursor [123] are widely conserved among mammalian taxa in the D2 and D4 VWD domains (panels D2, D4), but the D1 domain site is not conserved in myomorph rodents (panel D1). Numbers denote amino acid positions of the consensus sequence downstream of the alignment’s start at the D0 domain. Note the P→L/V substitution, driven by positive selection (sites denoted by asterisks), in all 11 myomorph species, but conservation of P in the others. Note also in the rodent species the downstream amino acid substitutions in D1 as well as the substitutions both upstream and downstream of the cleaved DP in D4, also driven by positive selection, reflecting the accelerated divergence of *Zan* in these animals. **B.** Species heterogeneity of zonadhesin D3 and D3p domain polypeptides. Shown are western blots of resolved sperm proteins from nine species, representing Orders Rodentia, Lagomorpha, Carnivora, Perissodactyla, Cetartiodactyla, and Primates, probed as indicated with affinity-purified antibody to the mouse zonadhesin D3 VWD domain (anti-muD3) or the D3p18 partial VWD domain (anti-muD3p18). Migration of size markers (M_*r*_ × 10^−3^) is indicated on the left. Species abbreviations are as follows: Mu, mouse; Rt, Norway rat; Ha, Syrian hamster; GP, guinea pig; Rb, rabbit; Dg, dog; Eq, horse; Po, pig; Hu, human. Note with anti-muD3 the detection of the well-characterized M_*r*_ 105,000 zonadhesin D3 polypeptides of porcine and equine spermatozoa [90, 124, 125], the detection of similarly sized D3 polypeptides in human, dog, and rabbit spermatozoa, and the detection of multiple larger polypeptides in rodent spermatozoa. Note with anti-muD3p18 the detection only in the three species of myomorph rodents of high M_*r*_ D3p18 immunoreactivity (>160,000 M_*r*_), including the well-characterized M_*r*_ 300,000 polypeptide of mouse and hamster spermatozoa [62, 126]

Multiple alignment of protein sequences encoded by 106 *Zan* DNAs, with the corresponding sequence of soft-shelled turtle *ZanL* as outgroup, produced a phylogenetic tree nearly identical to that obtained from DNA alignments (Additional file 2: Fig. S1). The alignment readily evinced regions of high protein sequence variation, with characteristic sequences corresponding to taxonomic groups, including insertions unique to certain taxa (e.g., a 4–11 residue insertion in the D1 domain only in myomorph rodents and a 4 residue insertion in the D3 domain only in bats), as well as loss of an otherwise conserved proteolytic processing site in the D1 domain only in myomorph rodents (Fig. 9, upper panel). The loss of the D1 processing site, together with differences in other posttranslational events such as glycosylation, manifested as striking levels of species heterogeneity in the sizes of zonadhesin D3 polypeptides produced in the precursor’s maturation (Fig. 9, lower panel). Importantly, the P➔L/V replacement in the D1 domain’s otherwise conserved Y^114^GDPH processing site proved to be a result of positive selection (Additional file 1: Table S5), suggesting the change is functionally important, consistent with findings of previous studies [74, 107].

**Fig. 10:**
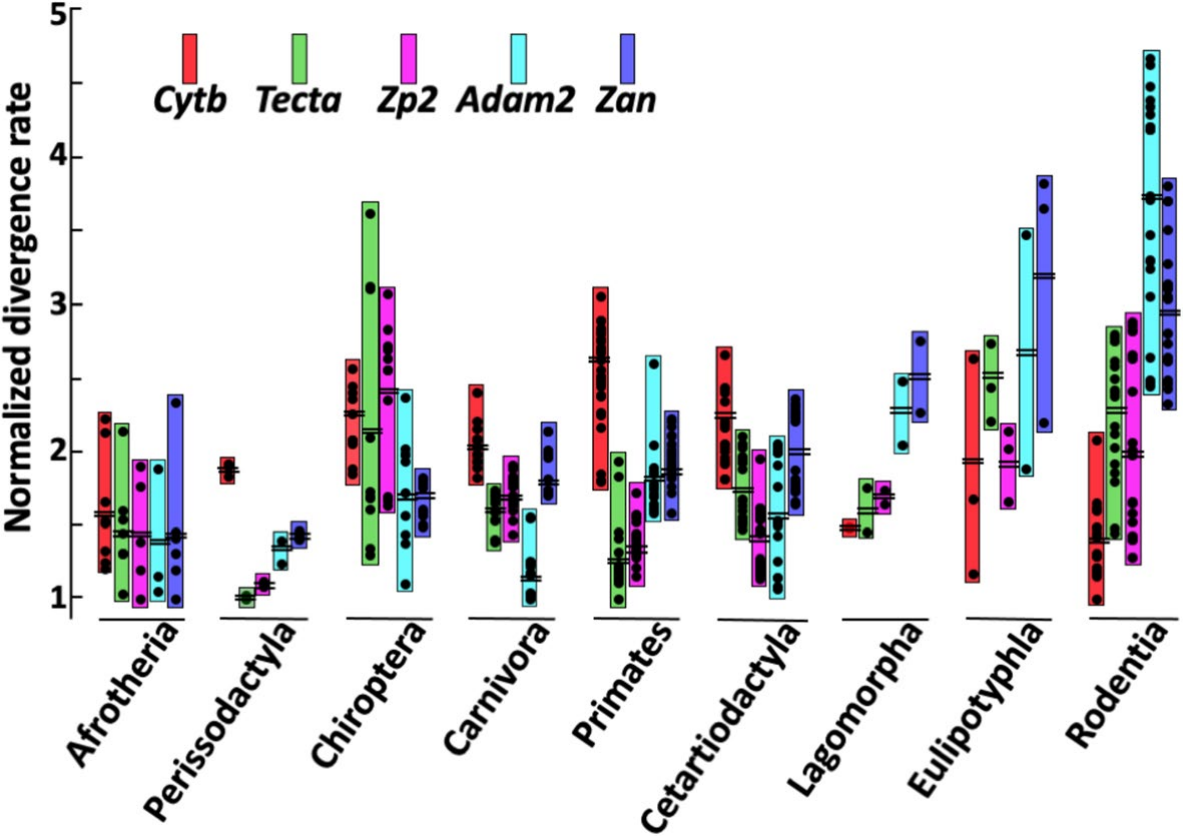
Variation of *Zan* DNA sequence divergence rate among species. Shown are the divergence rates of *Cytb*, *Tecta*, *Zp2*, *Adam2*, and *Zan* from each species (filled black circles), calculated from branch lengths back to the originating node of the species’ respective Superorders, and normalized to the rate of the species with the slowest divergence for each gene, i.e., normalized rate = 1.0 for *Cytb* in Norway rat (*Rattus norvegicus*), for *Tecta* in donkey (*Equus asinus*), for *Zp2* in elephant (*Loxodonta africana*), for *Adam2* in cheetah (*Acinonyx jubatus*), and for *Zan* in Florida manatee (*Trichechus manatus*). Data are grouped by taxa (Superorder Afrotheria and eight individual Orders) represented by at least three species. Double horizontal hashes denote mean divergence rates for each gene in each taxon. Note the low average *Zan* divergence rate in Afrotheria, which comprises six Orders with only 89 extant species, and the much higher rates in Eulipotyphla and Rodentia, which together comprise 3079 (>50%) of 6111 currently recognized, extant Eutherian species [127]

Although *Zan* DNA and protein sequence divergence correlated closely with presumptive species divergence, branch length differences suggested *Zan* divergence rate differed among the compared species. A rank-rate plot of *Zan* divergence since origination of the species’ respective Superorders revealed an inflection between the 91 most slowly diverging species, which included the 90 species from all Orders except Rodentia and Eulipotyphla, and the 21 most rapidly diverging species, which included 18 of 19 rodent species comprising, in part, all 11 species from Suborder Myomorpha (Additional file 2: Fig. S2). Among species in Superorder Afrotheria and eight other Eutherian Orders with at least three *Zan* sequences, five of the six genes diverged with normalized dynamic ranges of 3.6 for *Zan*, 4.4 for *Adam2*, 2.9 for *Zp2*, 3.4 for *Tecta*, and 2.9 for *Cytb* (Fig. 10); we omitted *Prm1* from this analysis because its Bayesian tree included many fewer species, lacked support at nearly half of its nodes, and contained so many polytomies and discrepant groupings as to make divergence rate calculations meaningless. Notably, the divergence rate range of *Tecta*, which encodes a protein important in audition, decreased to 2.0 exclusive of the three species of echolocating bats, illustrating possible selection-driven divergence in gene product function that generally diminishes the phylogenetic utility of adaptively evolving genes. Divergence rates for the five genes differed overall as well as between Orders and between species (Additional file 1: Tables S6-S12). *Zan* and *Adam2* divergence rate ranged highest in Rodentia and Eulipotyphla, whereas *Zp2* and *Tecta* ranged highest in Chiroptera, and *Cytb* divergence ranged highest in Primates. Within Orders, *Zan* and *Adam2* exhibited generally greater divergence range than *Zp2*, *Tecta*, or *Cytb*, with the exception only of *Tecta* in Chiroptera and *Cytb* in Primates. Furthermore, Rodentia, Eulipotyphla, and Lagomorpha exhibited higher average *Zan* and *Adam2* normalized divergence rate than *Zp2*, *Tecta*, or *Cytb* (*Ρ* < 0.0001; Additional file 1: Table S7). Finally, *Zan* divergence rate in Rodentia exceeded the rate in Superorder Afrotheria (*P* = 0.010; Additional file 1: Table S7) and in Orders Perissodactyla, Chiroptera, Carnivora, Cetartiodactyla, and Primates (*Ρ* < 0.0001; Additional file 1: Table S7), whereas *Adam2* divergence rate in Rodentia exceeded the rate in Orders Perissodactyla, Chiroptera, Carnivora, Cetartiodactyla, and Primates (*Ρ* < 0.008; Additional file 1: Table S8).

## Discussion

Four key findings in this study support the view that *Zan* is a speciation gene in Eutheria.

**Fig. 11:**
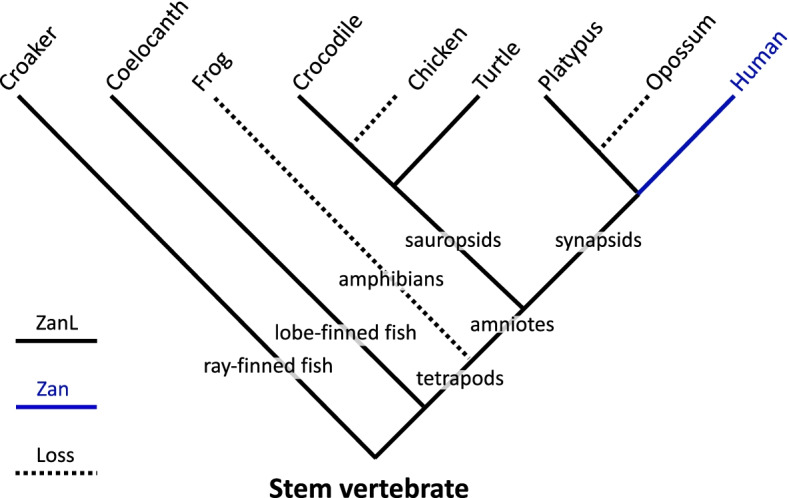
*Zan* ontogeny. In this proposed ontogeny, *Zan* and *ZanL* evolve from an ancient progenitor in a stem vertebrate, with the syntenic region of the progenitor comprising in part at least two genes present in progressively conserved *Zan* and *ZanL* syntenic loci of extant species. The *ZanL* descendant persists in ray- and lobe-finned fish and in reptiles (black branches) but is lost in amphibians, birds, and marsupials (dotted branches). *ZanL* also persists in Prototheria (monotremes) after divergence of the therian crown group but is lost in Metatheria (marsupials) after divergence from Eutheria, whereas authentic *Zan* evolves in the latter (blue branch) as a consequence of its neofunctionalization to a sperm-egg recognition molecule

1. *Genomic locus ontogeny suggests Zan represents a unique functional adaptation in placental mammals*. Absence of *Zan* in the otherwise conserved region spanning *AchE*–*Tfr2* in marsupials shows it is dispensable in this reproductively distinct Subclass of mammals. Indeed, *Zan*’s presence in Eutheria but absence from other vertebrates raises the question of when and how the gene originated. A plausible ontogeny (Fig. 11) emerges from presence of *Zan*-like genes (*ZanL*) in the largely conserved *AchE*–*Tfr2* locus of a monotreme (*Ornithorhynchus*) and in progressively poorly conserved loci of three reptiles (*Pelodiscus*, *Chrysemys*, and *Alligator*), a lobe-finned fish (*Latimeria*), and three ray-finned fish (*Larimichthys*, *Takifugu*, and *Danio*; Additional file 1: Table S1). In this proposed ontogeny, *Zan* and *ZanL* evolve from an ancestral *Zan/ZanL* precursor gene in stem vertebrates that is lost in amphibians, birds, and marsupials but persists as *ZanL* in monotremes, fishes, and reptiles because of some yet unidentified function, and persists as *Zan* in placental mammals because of its acquired function in sperm-egg recognition and species divergence [73].

(2) *Zan divergence directly reflects species diversification in Eutheria*. The *Zan* gene tree yields a more resolved phylogeny than trees constructed with gene sequences whose variation reflects either time passed since species diverged or unrelated functional evolution of their gene products rather than a direct contribution to speciation itself. Notably, the *Zan* tree yielded greater phylogenetic resolution than supertrees constructed from extensive gene sequence and morphometric data [15, 17, 18, 85–87] despite the prevailing view that single genes, especially genes evolving under selection, are poor phylogenetic markers. Consistent with its superior utility as a single-character phylogenetic marker, *Zan* yielded fewer candidate topologies than five comparison genes, including three other rapidly evolving reproductive/germ cell genes, a somatic gene similar in size and domain content to *Zan*, and a mitochondrial gene commonly used for phylogenetic analysis. Furthermore, only *Zan* candidate topologies resolved polytomies in the comparison supertree. Thus, among the genes studied, only *Zan* has evolved in strict concordance with Eutherian species phylogeny.

(3) *Positive selection drives Zan functional divergence.* Signatures of intense (*ω*_8_> 8.6) and pervasive (22% of sites) positive selection within the compared *Zan* sequences reveal that zonadhesin amino acid sequences diversified rapidly by adaptive evolution as expected for a speciation gene. Identified changes include numerous amino acid substitutions, insertions, and deletions characteristic of specific taxa, and altered processing sites hydrolyzed in the precursor’s functional maturation, all of which likely contribute to taxonomic variation in the protein’s species-specific egg recognition activity. The observed changes necessarily represent a minimal estimate of *Zan*’s species divergence, as our comparisons excluded sequences encoding expansions of partial VWD domains unique to species in rodent Superfamily Muroidea, which collectively represent 91% of the species in Suborder Myomorpha, 68% of the species in Rodentia, and 28% of Eutheria [80, 83, 127]. Indeed, we also detect strong signatures of positive selection in these expansions (Roberts et al., unpublished data), which may further serve to drive speciation in this most speciose Superfamily of mammals. The combined magnitude and pervasiveness of *Zan* diversifying selection exceed not only those of the five comparison genes in this study, but also those observed for noteworthy examples of rapid molecular evolution, including sperm protamine and other male reproductive proteins in Old World primates (*ω* ≤ 3 at 3.6% of sites [95]), bindin F-lectin repeat 1 in oyster spermatozoa (*ω* = 6.0 at 7.1% of sites [128]), various fertilization proteins of mammalian spermatozoa (*ω* = 3.9 at 3% of sites in fertilin α/ADAM1 [112], *ω* = 7.6 at 1% of sites in preproacrosin [112]), and even HIV envelope protein in rapid viral escape from neutralizing antibody (*ω* = 8.1 at <5% of sites [129]). In addition, by conducting our selection analysis on *Zan* sequence encoding VWD domain polypeptides with demonstrated ZP-binding activity [72, 73] among >100 taxonomically diverse species, we also observed substantially greater magnitude and pervasiveness of positive selection than previous comparisons of full or partial *Zan* sequences among many fewer species from a limited range of taxa (*ω* = 1.6–3.6 at <2% of sites among 12 or fewer species, mostly primates, from five or fewer Orders [74, 107, 130–132]). Finally, the *Zan* gene tree’s combined posterior support (95.5% of nodes) and aggregate intensity of positive selection (*ω* × *f* = 8.6 × 22.0 = 191) exceeded those of all other genes previously shown to have evolved by positive selection in Eutheria (and amenable to phylogenetic analysis), including the only previously identified speciation gene, *Prdm9* [114].

(4) *Zan divergence rate variation generally reflects species richness of Eutherian Orders*. *Zan* divergence rate ranged highest in Rodentia and Eulipotyphla, which together comprise more than half of the >6000 currently recognized placental species, and lowest among the six Orders of Afrotheria, which comprise fewer than 100 extant species [127]. Of the other genes analyzed, only *Adam2*’s ordinal divergence rate also generally correlated with species richness.

The foremost criterion for identification of speciation genes is a disproportionate contribution to species divergence. Our detailed phylogenetic analyses show that *Zan* meets this criterion. Among the genes we studied, including seven encoding other gamete-specific proteins with known or suspected functions in sperm-egg interactions (*Acr*, *Adam2*, *Izumo1*, *Juno*, *Spam1*, *Zp3*, and *Zp2*) as well as a rapidly evolving mitochondrial gene commonly used for phylogenetic analysis (*Cytb*), only *Zan* exhibited the combined phylogenetic attributes expected of a speciation gene. Because adaptive evolution of a gene directly manifests as functional divergence of its product, the extraordinary congruence between *Zan* evolution and species diversification must reflect a function of zonadhesin in speciation. Considered in reverse, what selective forces other than a function in speciation could explain the direct relationship between *Zan* adaptive evolution and species divergence? The lack of such a direct association among our comparison genes highlights the fact that their molecular evolution, whether conservative, neutral, or adaptive, reflects their involvement in processes other than speciation. Indeed, given the intensity and pervasiveness of *Zan*’s evolution by positive selection, it almost certainly would be a particularly bad phylogenetic marker were it not a speciation gene. Only two of the comparison genes (*Adam2* and *Tex14*) that have evolved by positive selection produced a tree with more than 90% posterior support, and five of the genes (*Prm1*, *Prm2*, *Ccl1*, *Sprr4*, and *Spink2*) among those that have evolved by high aggregate positive selection (*ω* × *f* >40) produced trees with less than 60% posterior support. Furthermore, many of the comparison genes also produced trees with supported polytomies and grossly incongruent topologies in comparison to widely accepted taxonomic relationships established by supertree studies (Roberts et al., unpublished), further confirming their poor phylogenetic utility. Finally, the *Zan* gene tree’s resolution at deep nodes in the mammalian phylogeny stands in stark contrast to the characteristics of rapidly evolving genes in general, which typically yield good resolution of terminal branches (because a fast clock is needed to measure the timing of more recent events) but not at earlier divergence events (because in the absence of relevant selection the numerous accumulated changes produce reversions). Thus, *Zan* molecular evolution appears to have promoted species divergence events at all taxonomic levels in the Eutherian lineage. In sum, the collective characteristics of *Zan*’s evolution, specifically its remarkable utility as a single gene marker (evident as high resolution and minimal topological ambiguity), rapid evolution by pervasive and intense positive selection, and speciosity-consistent variation in ordinal divergence rate, strongly support the view that it contributed disproportionately to speciation of placental mammals, and thus fulfills the fundamental criterion for identity as a speciation gene.

Definitions or expected properties of speciation genes also invariably include a contribution to reproductive isolation or, synonymously, to restriction of gene flow between incipient species [3, 29, 30, 35, 38, 49, 51]. Consistent with that expectation, zonadhesin’s function in fertilization directly implicates it as a mediator of reproductive isolation. Zonadhesin is a surface component of the sperm acrosomal matrix where, along with other ZP-binding proteins, it interacts with the ZP at the onset of acrosomal exocytosis [60, 129, 133, 134]. However, among known gamete adhesion molecules in placental mammals, zonadhesin is unique in its ability to bind directly and in a species-specific manner to the egg’s ZP [72, 73], and to confer species specificity to sperm-ZP adhesion [62]. Notably, in co-insemination studies, spermatozoa from *Zan* knockout mice adhere readily to ZP surrounding eggs from other species whereas wild-type spermatozoa do not, even though *Zan*-null and wild-type cells recognize conspecific mouse eggs identically [62]. These sperm competition experiments, deemed necessary for reliable establishment of “sperm precedence” [79] (a recognized mechanism of prezygotic reproductive isolation [132–140]), revealed not only that gamete recognition in placental mammals does indeed occur with some degree of species specificity (previous cross-species comparisons were fraught with technical difficulties arising from species differences in optimal conditions for fertilization in vitro), but also that zonadhesin is both necessary and sufficient to confer that specificity [62]. Thus, given its apparent contribution to reproductive isolation, *Zan* meets a second major criterion for identity as a speciation gene.

Nosil and Schluter [30] proposed two additional criteria for identification of animal speciation genes: (1) quantification of the gene’s effect size on reproductive isolation and (2) demonstration that gene divergence occurred before completion of speciation. Many speciation gene candidates have failed to meet these criteria, particularly the requirement for quantification of effect size, which for prezygotic barriers is more difficult to assess than it is for postzygotic barriers such as hybrid sterility (hence the preponderance of identified speciation genes that act postzygotically [29, 30, 57];). Indeed, abalone *Lysin* and sea urchin *Bindin*, as gamete recognition genes that act prezygotically, did not meet the stricter criteria because their effect size is unknown [30]. Similar to *Lysin* and *Bindin*, *Zan* acts prezygotically, and its effect size on reproductive isolation remains unknown. Nevertheless, our branch length analyses do provide insight into effect size variation between Orders, with larger apparent effects in more rapidly diverging Orders, as expected for a speciation gene. Furthermore, even a small effect on reproductive isolation acting over a long period of time can contribute disproportionately to speciation, so genes that act in this fashion do fulfill the major criteria for identity as a speciation gene whether or not their effect size has been explicitly quantified.

*Zan* differs from the relatively small number of known animal speciation genes, as it appears to have acted across a wide range of taxa (possibly throughout the evolution of placental mammals). Most speciation genetics studies focus on identifying barriers to gene flow between pairs of races, strains, or species that differ in relatively few characters [3, 29, 39, 57–59, 138]. The emphasis on pairs or small numbers of closely related organisms simulates divergence of incipient species, but also predictably results in identification of speciation genes whose action is restricted only to those same few species. Furthermore, such studies typically provide little or no information on the timing of gene divergence relative to speciation, which is difficult to determine in part because neither is a discrete event; indeed, phylogenetic studies must account for the fact that speciation is a protracted process that takes time to complete [139]. So how can relative timing of gene and species divergence be determined? Nosil and Schluter [30] proposed that evolution of “speciation genes should reflect species boundaries, whereas loci not involved in speciation might…show little phylogenetic resolution.” Interestingly, these authors did view *Lysin* and *Bindin* as having satisfied this divergence timing criterion, at least for a limited set of species, on the basis of comparatively little phylogenetic analysis (owing primarily to lack of genome sequence data from relevant taxa). In contrast, our robust phylogenetic analysis (more than 100 species) provides strong insight into the timing of *Zan*’s divergence relative to, and indeed seemingly coincident with, the timing of speciation events at all levels in the Eutherian tree. Thus, by approaching the identification of speciation genes phylogenetically in a wide range of taxa rather than in a small number of closely related species, we found that *Zan* also meets the additional “divergence timing” expectations of an animal speciation gene.

In sum, *Zan* satisfies not only the major criteria for identification as a speciation gene (disproportionate contribution to speciation and promotion of reproductive isolation) but also additional criteria uniquely applied in animals (effect size and divergence timing).

*Zan* is only the second speciation gene identified in mammals, and the first that acts prezygotically. The other known mammalian speciation gene, *Prdm9* (PR-domain 9), produces hybrid sterility between closely related mouse species, possibly as an essential component of a Dobzhansky-Muller incompatibility that contributes to incipient speciation [38, 114, 140, 141]. Discovered as a mouse germ cell gene (*Meisetz*) abundantly expressed upon entry into meiosis that encodes a histone H3 methyltransferase required for fertility [140], and subsequently identified by positional cloning as the active gene in a hybrid sterility locus (*Hst1*) that causes spermatogenic failure in *M. musculus*-*M. domesticus* hybrids [38], *Prdm9* shares certain characteristics with *Zan*. Like *Zan*, *Prdm9* encodes proteins comprising in part varying numbers, arrangements, and relatedness of domain repeats (7-13 zinc-finger domain repeats among 13 rodent and six primate species) and has evolved rapidly by positive selection [38, 140, 141]. Indeed, among all positively selected genes we subjected to comprehensive phylogenetic and selection analysis using sequences from ≥100 species (where available), only *Prdm9*, *Slc6a5*, *Zp2*, *Zp3*, *Tcte1*, and *Tex14* approached *Zan*’s combined resolution (posterior support) and aggregate intensity of positive selection (*ω* × *f*). Unlike *Zan*, however, *Prdm9* acts at the genomic level as a major determinant of recombination hotspot distribution and produces only transient incompatibility between diverging species [141]. Thus, *Zan*’s apparently persistent action in a discrete, prezygotic cellular event throughout the diversification of Eutheria sets it apart mechanistically as a unique, “trans-taxa” speciation gene.

Reproduction varies dramatically among species, with unique reproductive specializations constituting the defining traits of some taxa (e.g., the placenta in Eutheria). Accordingly, reproductive proteins exhibit high rates of evolution by positive selection [24, 107, 112, 142–144]. However, in contrast to gamete recognition molecules that can potentially confer a direct barrier to gene flow, many if not most rapidly evolving reproductive proteins mediate processes that may contribute to reproductive isolation only indirectly, if at all (for example, Transition protein 2, Protamines P15 and 2, Sperm protein 10, Acrosin, and TPX1/CRISP2 [24, 72, 73, 95, 111, 145–147]). Consistent with that view, these genes exhibited relatively low phylogenetic resolution and overall intensity of positive selection in our support vs. selection analysis. Our findings show that in mammals, as in marine invertebrates, rapid evolution of reproductive protein that mediates gamete recognition confers fertilization species specificity that directly serves to promote prezygotic reproductive isolation. Thus, internally fertilizing species appear to evolve by this mode of reproductive isolation even though other prezygotic processes can prevent insemination altogether, for example mating barriers arising from anatomical incompatibilities or differences in courtship behavior or breeding seasons [148].

Our study does not provide insight into the function of the *Zan* progenitor. No tissue expression data are available for the reptile and *Latimeria ZanL* genes identified, so we cannot definitively rule out the possibility that their products are sperm proteins. However, fish *ZanL* cannot act as *Zan* does in gamete interactions because fertilization in teleosts occurs by penetration of acrosomeless spermatozoa through the egg micropyle, a process that in general exhibits little if any species specificity [149, 150]. Consistent with that inference, the *ZanL* genes we identified in *Takifugu* and *Danio* correspond to pufferfish and zebrafish *Zan*-related loci that Hunt et al. [151] showed to be strongly expressed in zebrafish gut but not in testis or ovary. Thus, although the *Takifugu* and *Danio ZanL* genes may have descended directly from our proposed *Zan*/*ZanL* ancestor, their gene products likely function in teleost fish primarily as gut mucins, and not as gamete recognition molecules. We therefore propose that *Zan* arose in Eutheria by repurposing of a gene from stem vertebrates early in or coincident with the divergence of Eutheria from Metatheria, suggesting a contribution not only to species divergence within Eutheria but possibly also to the origination of the entire placental clade.

Variable action of selection influences divergence of any given trait, so single-character markers, especially those that evolve under selection, are generally considered unreliable for phylogenetic studies (a view confirmed by the low support we observed for the majority of trees constructed from single, positively selected genes). Contrary to this conventional wisdom, however, we found that *Zan* is an extraordinarily reliable single-character marker for speciation in placental mammals owing to its function as a trans-taxa speciation gene. Thus, *Zan* sequence comparisons may prove useful for resolving uncertainties in the relative timing of speciation events not only within Orders but also at more basal nodes in the placental phylogeny [18]. Indeed, our branch length analyses showed that average *Zan* sequence divergence rate among Eutherian Orders generally correlated with species richness, suggesting that the rate differences we observed reflect variation in *Zan*’s effect size as a speciation gene across different taxa [30] and that divergence rate could be calibrated for estimating when individual speciation events occurred. Unfortunately, quantitative analysis of that association proved impossible owing to lack of accepted ordinal speciation rates, which are difficult to estimate in part because they depend on extinction rates skewed by the “pull of the recent,” and in part because lineages-through-time plots underestimate rates of recent divergences that take time to complete [139]. Also, average speciation rate for the evolution of a lineage does not reflect the dynamic rate variation likely to result from action of speciation genes subjected to episodic positive selection [152]. Nevertheless, for Eutheria in general, it should still be possible to generate highly resolved phylogenies by comparing, as done here, *Zan* sequences spanning the full VWD domains among more extensive sets of species.

Future phylogenetic studies using *Zan* as a single-character marker need not focus solely on sequences shared by all placental species as done here. The presence of partial VWD domains (D3p [80, 83]; only in Superfamily Muroidea of rodent Suborder Myomorpha suggests that D3p duplications represent a dramatic and comparatively recent development in the molecular evolution of *Zan* that accelerated species diversification of this highly speciose taxon, which diverged from Superfamily Dipodoidea ~66 Myr ago [103]. The number of D3p domains detected thus far among muroid species varies from 9 to 24; simply comparing the ontogeny of their expansions in these animals may provide insight into the evolution and diversification of Muroidea. Importantly, D3p domains in these species likely contribute to egg recognition, as affinity-purified antibody to the D3p18 domain inhibits mouse sperm-egg adhesion [73]. Furthermore, mouse zonadhesin is highly autoimmunogenic [153], suggesting *Zan*’s rapid evolution, including D3p expansions, outraces the immune system’s ability to establish tolerance [154]. Thus, the partial VWD domains in some rodents provide additional opportunity not only for greater phylogenetic resolution of those species, but also for characterizing the protein-protein interactions underlying the species specificity of fertilization itself.

## Conclusions

In sum, *Zan* is a speciation gene that promoted macroevolution widely among placental mammals, as shown by the remarkable concordance of *Zan* molecular and mammalian species phylogenies, the rapid evolution of *Zan* by positive selection, and the contribution of the *Zan* gene product to post-mating, prezygotic reproductive isolation. And because *Zan* functional evolution directly reflects its contribution to species divergence, *Zan* may prove to have great utility as a single-character marker for resolving polytomies that remain in the mammalian phylogenetic tree.

## Methods

### Computational analyses

We conducted computationally intensive processes on supercomputer clusters Quanah (467 Dell PowerEdge C6320 nodes) or Hrothgar (100 Dell PowerEdge C6220 II nodes) accessed through the Texas Tech University High Performance Computing Center. These analyses included TBLASTX searches, T-coffee alignments, MrBayes analyses, and PAML selection tests. All statistical tests were performed using IBM SPSS STATISTICS 25.

### Database mining for Zan sequences

We first retrieved nucleotide sequences annotated as “zonadhesin” in GenBank or Ensembl by word query, then identified authentic *Zan* in 112 Eutherian mammals representing 17 of 19 Eutherian mammal Orders as described in “Results.” Because searches of non-Eutherian (*O. anatinus*, *M. domestica*, *Sarcophilus harrisi*, and *Macropus eugenii*) genomic databases (GenBank and Ensembl) yielded no *Zan* gene in the conserved mammalian *AchE*–*Tfr2* locus [80], we subsequently compared corresponding *AchE*–*Tfr2* syntenic loci between representative Eutherian mammals and non-Eutherian genomes (listed above) to search for *Zan* specifically between the genes *Epo* (erythropoietin) and *Ephb4* (ephrin b4 receptor). Finally, to identify possible distantly related *Zan* remnants in the *Epo*–*Ephb4* syntenic region and *AchE*–*Tfr2* locus, TBLASTX 2.8.0+ searches aligned and queried all possible reading frames of the aforementioned genomic reads, using default parameters and reward/penalty ratio (1/−1) to detect more divergent sequences [155].

To determine if *Zan* absence in several non-Eutherian genomes might be an artifact of genome assembly, we queried raw genomic reads of various vertebrates (*O. anatinus* assembly ornAna2, *M. domestica* assembly MonDom5, *Sarcophilus harrisi* assembly sarHar1, *Macropus eugenii* assembly macEug2, *Crocodylus porosus* assembly CroPor_comp1, *G. gallus* assembly galGal6, *Anolis carolinensis* assembly anoCar5, and *X. laevis* assembly xenLae2) by TBLASTX search with the 112 species Eutherian *Zan* nucleotide alignment (see below) and with a six species (*Rattus norvegicus*, *Bos taurus*, *Sus scrofa*, *Myotis lucifugus*, *Canis lupus familiaris*, and *Dasypus novemcinctus*) *ADAM3* (a germ cell-specific gene expressed widely among vertebrate taxa) nucleotide alignment for validation of the approach. To identify divergent, zonadhesin-like candidate progenitors in non-mammals, we queried NCBI non-redundant protein sequences by BLASTp 2.8.0+ [155] with the least derived *Zan* protein (*Dasypus novemcinctus*), then evaluated synteny to authentic *Zan* by inspection of their corresponding genetic loci in NCBI Genome Data Viewer.

### Sequence alignments

We aligned authentic *Zan* nucleotide sequences (Additional file 1: Table S2) encoding the zonadhesin protein’s von Willebrand D0, D1, D2, D3, and approximately the first 25% of D4 domains (range 330–1560 nts each) using T-coffee software in Meta-coffee mode to align with multiple algorithms, consolidate the output into a single model, and produce a local estimation of consistency with the individual alignment from which it was derived [156]. To confirm correct reading frames and detect premature stop codons, we translated the aligned sequences in MEGA X [157]. All species descriptions, sequence alignments, and resultant trees are deposited in the DRYAD Digital Repository [123].

### Phylogenetic analyses

We examined 88 maximum likelihood models with the hierarchical likelihood ratio tests with Akaike Information Criterion-correction in jModelTest2.1.10 [158] to detect the best-fit model of nucleotide substitution (Additional file 1: Table S2), and identified GTR+Γ+I as the most appropriate model. Because authentic *Zan* proved to be absent from non-mammals, we selected the *ZanL* gene from Chinese soft-shelled turtle (*Pelodiscus sinicus*) as outgroup in the *Zan* alignment. As outgroups for all other phylogenies, we selected orthologs also from reptiles where possible (*Acr*, *Adam2*, *Adam32*, *Adgre1*, *C5ar1*, *Cfh*, *Cr2*, *Crisp2*, *Cytb*, *Gpr50*, *Izumo1*, *Man2b1*, *Prdm9*, *Slc6a5*, *Spaca6*, *Spam1*, *Spink2*, *Tas1r2*, *Tchhl1*, *Tcn1*, *Tcte1*, *Tecta*, *Tectb*, *Tex11*, *Tex14*, *Wbp2nl*, *Zp2*, *Zp3*). Where no orthologous reptilian sequences were available, we used an amphibian as outgroup for *Juno*, avian species as outgroups for *Adam18*, *Ccl1*, and *Mgam*, a monotreme outgroup for *Ccdc54*, marsupials as outgroups for *Lelp1*, *S100a2*, *Prm1*, and *Sprr4*, xenarthrans for *Tnp2* and *Usp26*, and an afrotherian for *Prm2*. To perform likelihood analysis under a Bayesian inference model, we used MrBayes 3.2.6 [159] with the following options: 2 independent runs with four chains, one cold and three heated (Metropolis-coupled Markov chain Monte Carlo numerical method), 10 million generations, and sample frequency every 100th generation from the last 750,000 generated, then constructed a consensus tree (50% majority rule) from the remaining trees and plotted posterior probability values on the topology in FigTree 1.4.4 [160].

### Phylogenetic comparisons

To determine if gene trees recapitulated mammalian evolutionary relationships, we compared their topologies to an established mammalian species supertree phylogeny [15] by both global and intra-ordinal Shimodaira-Hasegawa (SH) and Shimodaira Approximately Unbiased (AU) tests [161]. The supertree [15] was constructed from extensive gene sequence and morphometric data and is very well-represented taxonomically, so we first pruned it in Phylomatic v.3 [162] to only those species represented in each of the *Zan*, *Adam2*, *Zp2*, *Prm1*, *Tecta*, and *Cytb* phylogenies, we input the unconstrained gene and corresponding pruned, constrained supertree files to PAUP v4.0a166 [105], and we ran one-tailed SH and AU tests using automated model selection (Additional file 1:Table S3), 10,000 RELL (Resampling Estimated Log Likelihoods) bootstrap generations, and Bonferroni correction. We considered trees significantly different at *Ρ* < 0.05.

### Selection tests

To assess DNA sequence divergence for relative contribution of neutral evolution, negative selection, and positive selection, we used the CODEML program in the PAMLX desktop computer package or PAML4.9j supercomputer package depending upon dataset size [106]. We first calculated dN/dS ratios (*ω*, omega) from the codon alignments with two comparisons wherein the null model, M0, assumed one global ratio and constrained *ω* to be equal on all branches in the phylogeny. The initial comparison (M0 vs. M7) tested for neutrality, wherein M7 assumed independent *ω* ratios for all branches in the phylogeny. The subsequent comparison (M7 vs. M8) tested for selection, wherein M8 allowed *ω* > 1 and detected variation in *ω* among sites using a Bayes Empirical Bayes approach to calculate posterior probabilities for sites under selective pressures [106]. We then determined which model, M7 or M8, was most appropriate for each gene by likelihood ratio tests (LTRs), using a chi-squared distribution, degrees of freedom equaling 2, and statistical significance of *Ρ* < 0.05.

### Characterization of zonadhesin D3 and D3p polypeptides

We detected zonadhesin D3 polypeptides as previously described [72, 73, 129, 130] on western blots using monospecific antibodies to the mouse D3 domain [73], and similarly detected D3p polypeptides using monospecific antibodies to the mouse D3p18 domain [73, 153, 154]. Briefly, after washing spermatozoa twice with 20 mM NaHEPES, 130 mM NaCl, 1 mM NaEDTA, pH 7.5 by centrifugation at 900*g*, 10 min, 23 °C, and once at 10,000*g*, 1 min, 23°C to remove epididymal or seminal fluid, we extracted sperm pellets with 10 volumes of SDS-PAGE sample buffer containing 25 mM dithiothreitol. We then resolved the extracted, disulfide-reduced sperm proteins by SDS-PAGE on 4–10% linear gradient gels, blotted them to nitrocellulose membranes, probed the membranes for zonadhesin polypeptides with antigen-affinity-purified, domain-specific antibodies to the mouse zonadhesin D3 domain (amino acids Ile^2168^-Thr^2270^) or D3p18 domain (Cys^4502^-Lys^4621^) at 80 or 40 ng/μl, respectively in 10 mM TrisHCl, 150 mM NaCl, 0.1% (v/v) Tween 20, pH 7.5, and finally detected bound zonadhesin antibodies with horseradish peroxidase-conjugated anti-rabbit secondary antibody (Biosource International) diluted 1/50,000 in TBST, followed by chemiluminescence visualization (SuperSignal, Pierce Chemical Co.).

### Divergence rate comparisons

To assess differences in divergence rates between species, we visualized gene trees in FigTree 1.4.4 [160] and summed species’ branch lengths back to the origins of their corresponding Superorders. We then identified the correct tests for divergence rate ANOVA and subsequent post hoc pairwise comparisons by conducting Shapiro-Wilk and Levene’s tests for normality and homogeneity of divergence rate variances, respectively, between and among Eutherian Orders for each gene. Shapiro-Wilk test showed that the assumption of normality was valid for *Cytb* (*P* = 0.056) but not for the other five genes individually or all six genes collectively (*P* < 0.0001). Levene’s test showed that the assumption of homoscedasticity was valid for *Prm1* (*Ρ* = 0.578) but not for the other five genes individually (all *Ρ* < 0.0001) or all six genes collectively (*Ρ* < 0.0001). Because *Cytb* divergence rates violated the assumption of homoscedasticity but were normally distributed, we conducted the more robust Welch’s *T*-test, which confirmed unequal variance of *Cytb* divergence rates (*Ρ* < 0.0001). Given the non-normal distributions and unequal variances of divergence rate data for *Zan*, *Adam2*, *Zp2*, and *Tecta*, we log-transformed the data for those four genes, then performed Kruskall-Wallis H non-parametric ANOVA on ranks to identify differences in global divergence rates between genes and within and among Orders for each gene. Finally, for post hoc analyses to determine stochastic domination of divergence rate between genes and between Orders within each gene [163], we performed Games-Howell tests on all six genes collectively and on the genes with unequal variances in rates individually (*Zan*, *Adam2*, *Zp2*, *Tecta*, and *Cytb*), and Ryan’s Q test on *Prm1*.

### Quantification and statistical analyses

Statistical details in Additional file 1: Tables S6-S12 state how normalization was achieved for the datasets, identify statistical tests used, list exact value of *n* and what it represents, and define relevant terms. For all tests, we rejected the null hypothesis (no difference) at *Ρ* < 0.05, except where multiple comparisons necessitated Bonferroni correction of *α* (topology tests). For posterior support probabilities, we accepted significance at *Ρ* ≥ 0.95. All statistical analyses for selection tests and divergence rate data are described in the “Methods” section above. Statistical analyses of divergence rate data performed using SPSS are described in Additional file 1: Tables S6-S12.

## Supplementary Information

Additional file 1. Comprises supplemental tables S1-S12 listing search results, method parameters and models, and numerical results of extensive phylogenetic and topological comparisons.

Additional file 2. Comprises supplemental figures S1-S3 illustrating linear comparison of mouse and opossum *Zan* loci, a zonadhesin protein sequence tree, and *Zan* divergence rate ranked by species, respectively.

Additional file 3. Presents full results narratives for topological comparisons of the *Adam2*, *Zp2*, *Prm1*, *Tecta*, and *Cytb* gene trees, as well as the DRYAD DOI and URL for shared data, including accession numbers for all database sequence files.

Additional file 4. Comprises the peer review history.

## Data Availability

The species and accession numbers, nucleotide and protein alignments, and resultant trees are available in the DRYAD Digital Repository [123] accessible at URL https://datadryad.org/stash/dataset/doi:10.5061/dryad.44j0zpcdf.

## References

[1] Dobzhansky T (1937). Genetics and the origin of species.

[2] Mayr E (1942). Systematics and the origin of species from the viewpoint of a zoologist.

[3] Orr HA, Masly JP, Presgraves DC (2004). Speciation genes. Curr Opin Genet Dev.

[4] Butlin RK, Ritchie MG (2009). Genetics of speciation. J Hered.

[5] McNiven VTK, LeVasseur-Viens HLN, Kanippayoor RL, Laturney M, Moehring AJ (2011). The genetic basis of evolution, adaptation and speciation. Mol Ecol.

[6] Seehausen O, Butlin RK, Keller I, Wagner CE, Boughman JW, Hohenlohe PA (2014). Genomics and the origin of species. Nat Rev Genet.

[7] Castillo DM, Barbash DA (2017). Moving speciation genetics forward: modern techniques build on foundational studies in *Drosophila*. Genetics.

[8] Darwin C (1859). The origin of species.

[9] Eldredge N, Gould SJ, Eldredge N, Gould SJ, Schopf TJ (1972). Chapter 5: punctuated equilibria: an alternative to phyletic gradualism. Models in Paleobiology.

[10] Ohta T (1992). The nearly neutral theory of molecular evolution. Annu Rev Ecol Syst.

[11] Baker RJ, Bradley RD (2006). Speciation in mammals and the genetic species concept. J Mammal.

[12] Schluter D, Conte GL (2009). Genetics and ecological speciation. Proc Natl Acad Sci U S A.

[13] Sanderson MJ, Purvis A, Henze C (1998). Phylogenetic supertrees: assembling the trees of life. Trends Ecol Evol.

[14] Bininda-Emonds OR, Gittleman JL, Steel MA (2002). The (super) tree of life: procedures, problems, and prospects. Annu Rev Ecol Syst.

[15] Bininda-Emonds OR, Cardillo M, Jones KE, MacPhee RDE, Beck RMD, Grenyer R (2007). The delayed rise of present-day mammals. Nature.

[16] Bininda-Emonds OR (2004). The evolution of supertrees. Trends Ecol Evol.

[17] Springer MS, Stanhope MJ, Madsen O, de Jong WW (2004). Molecules consolidate the placental mammal tree. Trends Ecol Evol.

[18] Foley NM, Springer MS, Teeling EC (2016). Mammal madness: is the mammal tree of life not yet resolved? Phil. Trans R Soc B.

[19] Upham NS, Esselstyn JA, Jetz W (2019). Inferring the mammal tree: species-level sets of phylogenies for questions in ecology, evolution, and conservation. PLoS Biol.

[20] Dobzhansky T (1973). Nothing in biology makes sense except in the light of evolution. Am Biol Teacher.

[21] Dobzhansky T, Ayala FJ, Dobzhansky T (1974). Chance and creativity in evolution. Studies in the Philosophy of Biology.

[22] Wolf JBW, Lindell J, Backstrom N (2010). Speciation genetics: current status and evolving approaches. Philos Trans R Soc B.

[23] Wu CI (2001). The genic view of the process of speciation. J Evol Biol.

[24] Swanson WJ, Vacquier VD (2002). The rapid evolution of reproductive proteins. Nat Rev Genet.

[25] Palumbi SR (2009). Speciation and the evolution of gamete recognition genes: pattern and process. J Hered.

[26] Rieseberg LH, Blackman BK (2010). Speciation genes in plants. Ann Bot.

[27] Wang X, Que P, Heckel G, Hu J, Zhang X, Chiang CU (2019). Genetic, phenotypic and ecological differentiation suggests incipient speciation in two *Charadrius* plovers along the Chinese coast. BMC Evol Biol.

[28] Cracraft J, Johnston RF (1983). Species concepts and speciation analysis. Current Ornithology.

[29] Wu CI, Ting CT (2004). Genes and speciation. Nat Rev Genet.

[30] Nosil P, Schluter D (2011). The genes underlying the process of speciation. Trends Ecol Evol.

[31] The Marie Curie Speciation Network (2012). What do we need to know about speciation?. Trends Ecol Evol.

[32] Wang X, He Z, Shi S, Wu CI (2020). Genes and speciation: is it time to abandon the biological species concept?. Natl Sci Rev.

[33] Ayala FJ, Tracey ML, Hedgecock D, Richmond RC (1974). Genetic differentiation during the speciation process in Drosophila. Evolution.

[34] Orr HA (1992). Mapping and characterization of a ‘speciation gene’ in *Drosophila*. Genet Res.

[35] Orr HA (2005). The genetic basis of reproductive isolation: insights from *Drosophila*. Proc Natl Acad Sci U S A.

[36] Mallet J (2006). What does *Drosophila* genetics tell us about speciation?. Trends Ecol Evol.

[37] Presgraves DC (2008). Sex chromosomes and speciation in *Drosophila*. Trends Genet.

[38] Mihola O, Trachtulec Z, Vlcek C, Schimenti JC, Forejt J (2009). A mouse speciation gene encodes a meiotic histone H3 methyltransferase. Science.

[39] Harrison RG (2012). The language of speciation. Evolution: Intl J Organ Evol.

[40] Meier JI, Marques DA, Mwaiko S, Wagner CE, Excoffier L, Seehausen O (2017). Ancient hybridization fuels rapid cichlid fish adaptive radiations. Nature Comm.

[41] Rieseberg LH, Willis JH (2007). Plant speciation. Science.

[42] Soltis PS, Soltis DE (2009). The role of hybridization in plant speciation. Annu Rev Plant Biol.

[43] Stebbins GL. Polyploidy, hybridization, and the invasion of new habitats. Ann Mo Bot Gard. 1985:824–32. 10.2307/2399224.

[44] Mallet J (2007). Hybrid speciation. Nature.

[45] Wood TE, Takebayashi N, Barker MS, Mayrose I, Greenspoon PB, Rieseberg LH (2009). The frequency of polyploid speciation in vascular plants. Proc Natl Acad Sci U S A.

[46] Pelé A, Rousseau-Gueutin M, Chèvre AM (2018). Speciation success of polyploid plants closely relates to the regulation of meiotic recombination. Front Plant Sci.

[47] Avise JC, Smith JJ, Ayala FJ (1975). Adaptive differentiation with little genic change between two native California minnows. Evolution.

[48] Byers KJ, Xu S, Schlüter PM (2017). Molecular mechanisms of adaptation and speciation: why do we need an integrative approach?. Mol Ecol.

[49] Orr HA, Presgraves DC. Speciation by postzygotic isolation: forces, genes and molecules. BioEssays. 2000;22(12):1085–94. 10.1002/1521-1878(200012)22:12<1085::AID-BIES6>3.0.CO;2-G. 10.1002/1521-1878(200012)22:12<1085::AID-BIES6>3.0.CO;2-G 11084624

[50] Ortiz-Barrientos D, Counterman BA, Noor MAF (2004). The genetics of speciation by reinforcement. PLoS Biol.

[51] Lassance JM, Groot AT, Liénard MA, Antony B, Borgwardt C, Andersson F (2010). Allelic variation in a fatty-acyl reductase gene causes divergence in moth sex pheromones. Nature.

[52] Via S (2009). Natural selection in action during speciation. Proc Natl Acad Sci U S A.

[53] Feder JL, Nosil P, Wacholder AC, Egan SP, Berlocher SH, Flaxman SM (2014). Genome-wide congealing and rapid transitions across the speciation continuum during speciation with gene flow. J Hered.

[54] Pfennig CA (2016). Reinforcement as an initiator of population divergence and speciation. Curr Zool.

[55] Coyne JA, Orr HA (2004). Speciation.

[56] Turelli M, Orr HA (2000). Dominance, epistasis, and the genetics of postzygotic isolation. Genetics.

[57] Morgan K, Harr B, White MA, Payseur BA, Turner LM (2020). Disrupted gene networks in subfertile hybrid house mice. Mol Biol Evol.

[58] Presgraves DC (2010). The molecular evolutionary basis of species formation. Nat Rev Genet.

[59] Turner LM, Harr B (2014). Genome-wide mapping in a house mouse hybrid zone reveals hybrid sterility loci and Dobzhansky-Muller interaction. eLife.

[60] Bi M, Wassler MJ, Hardy DM, Hardy DM (2002). Sperm adhesion to the extracellular matrix of the egg. Fertilization.

[61] Karr TL, Swanson WJ, Snook RR, Birkhead TR, Hosken DJ, Pitnick S (2009). Chapter 8: The evolutionary significance of variation in sperm–egg interactions. Sperm Biology: An Evolutionary Perspective.

[62] Tardif S, Wilson MD, Wagner R, Hunt P, Gertsenstein M, Nagy A (2010). Zonadhesin is essential for species specificity of sperm adhesion to the egg zona pellucida. J Biol Chem.

[63] Avella MA, Baibakov B, Dean J (2014). A single domain of the ZP2 zona pellucida protein mediates gamete recognition in mice and humans. J Cell Biol.

[64] Palumbi SR (1994). Genetic divergence, reproductive isolation, and marine speciation. Annu Rev Ecol Syst.

[65] Lessios HA (2011). Speciation genes in free-spawning marine invertebrates. Int Comp Biol.

[66] Swanson WJ, Vacquier VD (1997). The abalone egg vitelline envelope receptor for sperm lysin is a giant multivalent molecule. Proc Natl Acad Sci U S A.

[67] Galindo BE, Moy GW, Swanson WJ, Vacquier VD (2002). Full-length sequence of VERL, the egg vitelline envelope receptor for abalone sperm lysin. Gene.

[68] Aagaard JE, Vacquier VD, MacCoss MJ, Swanson WJ (2010). ZP domain proteins in the abalone egg coat include a paralog of VERL under positive selection that binds lysin and 18-kDa sperm proteins. Mol Biol Evol.

[69] Metz EC, Palumbi SR (1996). Positive selection and sequence rearrangements generate extensive polymorphism in the gamete recognition protein bindin. Mol Biol Evol.

[70] Zigler KS, McCartney MA, Levitan DR, Lessios HA (2005). Sea urchin bindin divergence predicts gamete compatibility. Evolution.

[71] Swanson WJ, Vacquier VD (1998). Concerted evolution in an egg receptor for a rapidly evolving abalone sperm protein. Science.

[72] Hardy DM, Garbers DL (1994). Species-specific binding of sperm proteins to the extracellular matrix (zona pellucida) of the egg. J Biol Chem.

[73] Hardy DM, Garbers DL (1995). A sperm membrane protein that binds in a species-specific manner to the egg extracellular matrix is homologous to von Willebrand factor. J Biol Chem.

[74] Herlyn H, Zischler H (2008). The molecular evolution of sperm zonadhesin. Int J Dev Biol.

[75] Howard DJ (1999). Conspecific sperm and pollen precedence and speciation. Annu Rev Ecol Syst.

[76] Howard DJ, Palumbi SR, Birge LM, Manier MK, Birkhead TR, Hosken DJ, Pitnick S (2009). Sperm and speciation. Chapt. 9. Sperm Biology: An Evolutionary Perspective.

[77] Arbogast BS, Slowinski JB (1998). Pleistocene speciation and the mitochondrial DNA clock. Science.

[78] Kumar S (2005). Molecular clocks: four decades of evolution. Nat Rev Genet.

[79] Sabeti PC, Schaffner SF, Fry B, Lohmueller J, Varilly P, Shamovsky O (2006). Positive natural selection in the human lineage. Science.

[80] Wilson MD, Riemer C, Martindale DW, Schnupf P, Boright AP, Cheung TL (2001). Comparative analysis of the gene-dense ACHE/TFR2 region on human chromosome 7q22 with the orthologous region on mouse chromosome 5. Nucleic Acids Res.

[81] Nishimura H, Myles DG, Primakoff P (2007). Identification of an ADAM2-ADAM3 complex on the surface of mouse testicular germ cells and cauda epididymal sperm. J Biol Chem.

[82] Long J, Li M, Ren Q, Zhang C, Fan J, Duan Y (2012). Phylogenetic and molecular evolution of the ADAM (A Disintegrin And Metalloprotease) gene family from *Xenopus tropicalis* to *Mus musculus*, *Rattus norvegicus*, and *Homo sapiens*. Gene.

[83] Gao Z, Garbers DL (1998). Species diversity in the structure of zonadhesin, a sperm-specific membrane protein containing multiple cell adhesion molecule-like domains. J Biol Chem.

[84] McKenna MC, Bell SK (1997). Classification of mammals above the species level.

[85] Murphy WJ, Eizirik E, O’Brian SJ, Madsen O, Scally M, Douady CJ (2001). Resolution of the early placental mammal radiation using Bayesian phylogenetics. Science.

[86] Springer MS, DeBry RW, Douady C, Amrine HM, Madsen O, de Jong WW (2001). Mitochondrial versus nuclear gene sequences in deep-level mammalian phylogeny reconstruction. Mol Biol Evol.

[87] Meredith RW, Janecka JE, Gatesy J, Ryder OA, Fisher CA, Teeling EC (2011). Impacts of the Cretaceous terrestrial revolution and KPg extinction on mammal diversification. Science.

[88] Steiner CC, Ryder OA (2011). Molecular phylogeny and evolution of the Perissodactyla. Zool J Linn Soc.

[89] Tsagkogeorga G, Parker J, Stupka E, Cotton JA, Rossiter SJ (2013). Phylogenomic analyses elucidate the evolutionary relationships of bats. Curr Biol.

[90] Bi M, Winfrey VP, Olson GE, Hardy DM (2003). Processing, localization, and binding activity of zonadhesin suggest a function in sperm adhesion to the zona pellucida during exocytosis of the acrosome. Biochem J.

[91] Glassey B, Civetta A (2004). Positive selection at reproductive ADAM genes with potential intercellular binding activity. Mol Biol Evol.

[92] Choi H, Jin S, Kwon JT, Kim J, Jeong J, Kim J (2016). Characterization of mammalian ADAM2 and its absence from human sperm. PLoS One.

[93] Gahlay G, Gauthier L, Baibakov B, Epifano O, Dean J (2010). Gamete recognition in mice depends on the cleavage status of an egg’s zona pellucida protein. Science.

[94] Dean J, Sawada H, Inoue N, Iwano M (2014). A ZP2 Cleavage model of gamete recognition and the postfertilization block to polyspermy. Sexual Reproduction in Animals and Plants.

[95] Wyckoff GJ, Wang W, Wu C-I (2000). Rapid evolution of male reproductive genes in the descent of man. Nature.

[96] Martin-Coello J, Dopazo H, Arbiza L, Ausio J, Roldan ERS, Gomendio M (2009). Sexual selection drives weak positive selection in protamine genes and high promoter divergence, enhancing sperm competitiveness. Proc R Soc B.

[97] Kasinsky HE, Eirin-Lopez JM, Ausió J (2011). Protamines: structural complexity, evolution and chromatin patterning. Protein Pept Lett.

[98] Lüke L, Vicens A, Tourmente M, Roldan ER (2014). Evolution of protamine genes and changes in sperm head phenotype in rodents. Biol Reprod.

[99] Bao J, Bedford MT (2016). Epigenetic regulation of the histone-to-protamine transition during spermiogenesis. Reproduction.

[100] Legan PK, Rau A, Kee JN, Richardson GP (1997). The mouse tectorins modular matrix proteins of the inner ear homologous to components of the sperm-egg adhesion system. J Biol Chem.

[101] Verhoeven K, Van Laer L, Kirschhofer K, Legan PK, Hughes DC, Schatteman I (1998). Mutations in the human α-tectorin gene cause autosomal dominant non-syndromic hearing impairment. Nat Genet.

[102] Alloisio N, Morle L, Bozon M, Godet J, Verhoeven K, Van Camp G (1999). Mutation in the zonadhesin-like domain of α-tectorin associated with autosomal dominant non-syndromic hearing loss. Eur J Hum Genet.

[103] Honeycutt RL, Nedbal MA, Adkins RM, Janecek LL (1995). Mammalian mitochondrial DNA evolution: a comparison of the cytochrome b and cytochrome c oxidase II genes. J Mol Evol.

[104] Stadler T, Bokma F (2013). Estimating speciation and extinction rates for phylogenies of higher taxa. Syst Biol.

[105] Swofford DL (2003). PAUP*: phylogenetic analysis using parsimony, version 4.0 b10.

[106] Yang Z (2007). PAML 4: phylogenetic analysis by maximum likelihood. Mol Biol Evol.

[107] Swanson WJ, Nielsen R, Yang Q (2003). Pervasive adaptive evolution in mammalian fertilization proteins. Mol Biol Evol.

[108] Torgerson DG, Kulathinal RJ, Singh RS (2002). Mammalian sperm proteins are rapidly evolving: evidence of positive selection in functionally diverse genes. Mol Biol Evol.

[109] Dufourny L, Levasseur A, Martine M, Callebaut I, Pontarotti P, Malpaux B, Monget P (2008). GPR50 is the mammalian ortholog of Mel1c: evidence of rapid evolution in mammals. BMC Evol Biol.

[110] Kosiol C, Vinar T, Fonseca RR, Hubisz MJ, Bustamante CD, Nielsen R, Siepe A (2008). Patterns of positive selection in six mammalian genomes. PLoS Genet.

[111] Raterman D, Springer MS (2008). The molecular evolution of acrosin in placental mammals. Mol Reprod Dev.

[112] Turner LM, Chuong EB, Hoekstra HE (2008). Comparative analysis of testis protein evolution in rodents. Genetics.

[113] Waddell LA, Lefevre L, Bush SJ, Raper A, Young R, Lisowski ZM, Mary E, McCulloch B, Muriuki C, Sauter KA, Clark EL, Irvine KM, Pridans C, Hope JC, Hume DA (2008). ADGRE1 (EMR1, F4/80) is a rapidly-evolving gene expressed in mammalian monocyte-macrophages. Front Immunol.

[114] Oliver PL, Goodstadt L, Bayes JJ, Birtle Z, Roach KC, Phadnis N (2009). Accelerated evolution of the *Prdm9* speciation gene across diverse metazoan taxa. PLoS Genet.

[115] Finn S, Civetta A (2010). Sexual selection and the molecular evolution of ADAM proteins. J Mol Evol.

[116] Grayson P, Civetta A (2013). Positive selection in the adhesion domain of *Mus* sperm Adam genes through gene duplications and function-driven gene complex formations. BMC Evol Biol.

[117] Grayson P (2015). Izumo1 and Juno: the evolutionary origins and coevolution of essential sperm-egg binding partners. R Soc Open Sci.

[118] Zhang Y, Li HQ, Yao YF, Liu W, Ni QY, Zhang MW, Xu HL (2015). Uneven evolutionary rate of the melatonin-related receptor gene (GPR50) in primates. Genet Mol Res.

[119] Goodwin ZA, de Guzman SC (2017). Recent positive selection in genes of the mammalian epidermal differentiation complex locus. Front Genet.

[120] Zhao Z, Campbell MC, Li N, Lee DSW, Zhang Zhang Z, Townsend JP (2017). Detection of regional variation in selection intensity within protein-coding genes using DNA sequence polymorphism and divergence. Mol Biol Evol.

[121] Cantsilieris S, Nelson BJ, Huddleston J, Baker C, Harshman L, Penewit K, Munson KM, Sorensen M, Welch AE, Dang V, Grassmann F, Richardson AJ, Guymer RH, Graves-Lindsay TA, Wilson RK, Weber BHF, Baird PN, Allikmets R, Eichler EE (2018). Recurrent structural variation, clustered sites of selection, and disease risk for the complement factor H (CFH) gene family. Proc Natl Acad Sci U S A.

[122] Moros-Nicolás C, Fouchécourt S, Goudet G, Monget P (2018). Genes encoding mammalian oviductal proteins involved in fertilization are subjected to gene death and positive selection. J Mol Evol.

[123] Roberts EK, Tardif S, Wright EA, Platt II, Roy N, Bradley RD, Hardy DM (2022). Rapid divergence of a gamete recognition promoted macroevolution of Eutheria. Datasets DRYAD.

[124] Hickox JR, Bi M, Hardy DM (2001). Heterogeneous processing and zona pellucida binding activity of pig zonadhesin. J Biol Chem.

[125] Tardif S, Brady JA, Breazeale KR, Bi M, Thompson LD, Bruemmer JE (2010). Zonadhesin D3-polypeptides vary among species but are similar in *Equus* species capable of interbreeding. Biol Reprod.

[126] Olson GE, Winfrey VP, Bi M, Hardy DM, NagDas SK (2004). Zonadhesin assembly into the hamster sperm acrosomal matrix occurs by distinct targeting strategies during spermiogenesis and maturation in the epididymis. Biol Reprod.

[127] Burgin CJ, Colella JP, Kahn PL, Upham NS (2018). How many species of mammals are there?. J Mammal.

[128] Moy GW, Springer SA, Adams SL, Swanson WJ, Vacquier VD (2008). Extraordinary intraspecific diversity in oyster sperm bindin. Proc Natl Acad Sci U S A.

[129] Frost SDW, Wrin T, Smith DM, Kosakovsky Pond SL, Liu Y (2005). Neutralizing antibody responses drive the evolution of human immunodeficiency virus type 1 envelope during recent HIV infection. Proc Natl Acad Sci U S A.

[130] Herlyn H, Zischler H (2005). Identification of a positively evolving putative binding region with increased variability in posttranslational motifs in zonadhesin MAM domain 2. Mol Phylogenet Evol.

[131] Herlyn H, Zischler H (2005). Sequence evolution, processing, and posttranslational modification of zonadhesin D domains in primates, as inferred from cDNA data. Gene.

[132] Gasper J, Swanson WJ (2006). Molecular population genetics of the gene encoding the human fertilization protein zonadhesin reveals rapid adaptive evolution. Am J Hum Genet.

[133] Gerton GL, Hardy DM (2002). Function of the sperm acrosome. Fertilization.

[134] Buffone MG, Foster JA, Gerton GL (2008). The role of the acrosomal matrix in fertilization. Int J Dev Biol.

[135] Klibansky LKJ, McCartney MA (2014). Conspecific sperm precedence is a reproductive barrier between free-spawning marine mussels in the northwest Atlantic mytilus hybrid zone. PLoS One.

[136] Firman RC, Simmons LW (2014). No evidence of conpopulation sperm precedence between allopatric populations of house mice. PLoS One.

[137] Castillo DM, Moyle LC (1899). Conspecific sperm precedence is reinforced, but postcopulatory sexual selection weakened, in sympatric populations of Drosophila. Proc Biol Soc.

[138] Ravinet M, Faria R, Butlin RK, Galindo J, Bierne N, Rafajlovic M (2017). Interpreting the genomic landscape of speciation: a road map for finding barriers to gene flow. J Evol Biol.

[139] Etienne RS, Rosindell J (2012). Prolonging the past counteracts the pull of the present: protracted speciation can explain observed slowdowns in diversification. Syst Biol.

[140] Hayashi K, Yoshida K, Matsui Y (2005). A histone H3 methyltransferase controls epigenetic events required for meiotic prophase. Nature.

[141] Brand CL, Presgraves DC (2016). Evolution: on the origin of symmetry, synapsis, and species. Curr Biol.

[142] Palumbi SR (1999). All males are not created equal: fertility differences depend on gamete recognition polymorphisms on sea urchins. Proc Natl Acad Sci U S A.

[143] Turner LM, Hoekstra HE (2006). Adaptive evolution of fertilization proteins within a genus: variation in ZP2 and ZP3 in deer mice (*Peromyscus*). Mol Biol Evol.

[144] Turner LM, Hoekstra HE (2008). Causes and consequences of the evolution of reproductive proteins. Int J Dev Biol.

[145] Hardy DM, Wild GC, Tung KSK (1987). Purification and initial characterization of proacrosins from guinea pig testes and epididymal spermatozoa. Biol Reprod.

[146] Hardy DM, Huang TTF, Driscoll WJ, Tung KSK, Wild GC (1988). Purification and characterization of the primary acrosomal autoantigen of the guinea pig epididymal spermatozoa. Biol Reprod.

[147] Foster JA, Gerton GL. Autoantigen 1 of the guinea pig sperm acrosome is the homologue of mouse Tpx-1 and human TPX1 and is a member of the cysteine-rich secretory protein (CRISP) family. Mol Reprod Dev. 1996;44(2):221–9. 10.1002/(SICI)1098-2795(199606)44:2<221::AID-MRD11>3.0.CO;2-5. 10.1002/(SICI)1098-2795(199606)44:2<221::AID-MRD11>3.0.CO;2-5 9115720

[148] Kaneshiro KY (1980). Sexual isolation, speciation and the direction of evolution. Evolution.

[149] Yanagimachi R, Knobil E, Neill JD (1994). Mammalian Fertilization. The Physiology of Reproduction.

[150] Killingbeck EE, Swanson WJ (2018). Egg coat proteins across metazoan evolution. Curr Top Dev Biol.

[151] Hunt PN, Wilson MD, Von Schalburg KR, Davidson WS, Koop BF (2005). Expression and genomic organization of zonadhesin-like genes in three species of fish give insight into the evolutionary history of a mosaic protein. BMC Genomics.

[152] Kumar S, Filipski AJ, Battistuzzi FU, Kosakovsky Pond SL, Tamura K (2012). Statistics and truth in phylogenomics. Mol Biol Evol.

[153] Wheeler K, Tardif S, Rival C, Luu B, Bui E, del Rio R (2011). Regulatory T cells control tolerogenic versus autoimmune response to sperm in vasectomy. Proc Natl Acad Sci U S A.

[154] Tung KSK, Harakal J, Qiao J, Rival C, Li JCH, Paul AGA (2017). Egress of sperm autoantigen from seminiferous tubules maintains systemic tolerance. J Clin Invest.

[155] Camacho C, Coulouris G, Avagyan V, Ma N, Papadopoulos J, Bealer K (2009). BLAST+: architecture and applications. BMC Bioinf.

[156] Notredame C, Higgins DG, Heringa J (2000). T-Coffee: A novel method for fast and accurate multiple sequence alignment. J Mol Biol.

[157] Kumar S, Stecher G, Li M, Knyaz C, Tamura K (2018). MEGA X: molecular evolutionary genetics analysis across computing platforms. Mol Biol Evol.

[158] Darriba D, Taboada GL, Doallo R, Posada D (2012). jModelTest 2: more models, new heuristics and parallel computing. Nat Methods.

[159] Ronquist F, Teslenko M, van der Mark P, Ayers DL, Darling A, Hohna S (2012). MrBayes 3.2: efficient Bayesian phylogenetic inference and model choice across a large model space. Syst Biol.

[160] Rambaut A (2018). FigTree v. 1.4.4: a graphical viewer of phylogenetic trees.

[161] Shimodaira H (2002). An approximately unbiased test of phylogenetic tree selection. Syst Biol.

[162] Webb CO, Donoghue MJ (2005). Phylomatic: tree assembly for applied phylogenetics. Mol Ecol Notes.

[163] Day RW, Quinn GP (1989). Comparisons of treatments after an analysis of variance in ecology. Ecol Mono.

