# Supplementary material for: Rapid divergence of a gamete recognition gene promoted macroevolution of Eutheria
Source: Genome Biol. 2022 Jul 11;23:155. doi: 10.1186/s13059-022-02721-y (PMC9275260; doi:10.1186/s13059-022-02721-y)
Supplement: Supplementary file 1 — Additional file 1. Comprises supplemental tables S1-S12 listing search results, method parameters and models, and numerical results of extensive phylogenetic and topological comparisons. [file 13059_2022_2721_MOESM1_ESM.pdf]

Table S1. BLASTp summary results for *Zan* sequence mining in non-mammals

Species	Common name	NCBI description	E val	Bitscore	Seq ID	Accession No.
Alligator sinensis	Chinese alligator	zonadhesin-like	1E-170	2405	64%	XP_025067289.1
Chrysemys picta bellii	Painted turtle	zonadhesin-like	0.00	3512	46%	XP_023969614.1
Pelodiscus sinensis	Chinese soft-shelled turtle	zonadhesin	0.00	3186	45%	XP_025045216.1
Latimeria chalumnae	Coelocanth	PREDICTED: zonadhesin	0.00	3564	44%	XP_014346149.1
Larimichthyes crocea	Yellow croaker	zonadhesin	0.00	2961	42%	KKF20408.1
Danio rerio	Zebrafish	zonadhesin iso-forms x2, x1	0.00	1249	42%	XP_021333481.1, XP_001921732.1

Table S2A. Global and Ordinal nucleotide substitution models for each gene. GTR=general time reversal; TVM=transversion model; HKY=Hasegawa-Kishino-Yano; +G=gamma; +I+G=inverse gamma. NA=no model because of limited taxon sampling.

**Dataset**	***Zan***	***Tecta***	***Cytb***	***Adam2***	***Zp2***	***Prm1***
Global	GTR+I+G	GTR+I+G	GTR+I+G	GTR+I+G	GTR+I+G	TVM+I+G
Afrotheria	GTR+G	GTR+I+G	GTR+I+G	NA	GTR+G	NA
Chiroptera	GTR+G	GTR+G	GTR+I+G	GTR+G	GTR+G	NA
Carnivora	GTR+G	GTR+I+G	GTR+I+G	GTR+G	GTR+G	NA
Primates	GTR+G	GTR+I+G	GTR+I+G	GTR+G	GTR+G	HKY85+G
Cetartiodactyla	GTR+G	GTR+I+G	GTR+I+G	GTR+G	GTR+G	GTR+G
Rodentia	GTR+I+G	GTR+I+G	GTR+I+G	GTR+I+G	GTR+I+G	GTR+G

Gene	Model of Evolution
Acr	TVM+I+G
Adam2	GTR+I+G
Adam18	TVM+I+G
Adam32	TPM3uf+I+G
Adgre1	GTR+I+G
C5ar1	GTR+I+G
Ccdc54	TVM+I+G
Ccl1	TPM3+G
Cfh	TIM3+I+G
Cr2	GTR+I+G
Crisp2	TVM+I+G
Cytb	GTR+I+G
Gpr50	TIM1+I+G
Izumo1	TIM2+I+G
Juno	GTR+I+G
Lelp1	TrN+G
Man2b1	TVM+I+G
Prdm9	TIM3+G
Prm1	TVM+I+G
Prm2	TIM2+I+G
S100a2	K80+G
Slc6a5	TVM+I+G
Spaca6	TIM2+G
Spam1	TPM3uf+I+G
Spink2	TPM3uf+I+G
Sprr4	TPM3uf+G
Tas1r2	GTR+I+G
Tchhl1	GTR+I+G
Tcn1	TVM+I+G
Tcte1	TPM2uf+I+G
Tecta	GTR+I+G
Tectb	TPM3uf+I+G
Tex11	TVM+G
Tex14	GTR+I+G
Tnp2	TIM3+I+G
Usp26	GTR+I+G
Wbp2nl	TIM1+I+G
Zan	GTR+I+G
Zp2	GTR+I+G
Zp3	TIM1+I+G

Table S2B. Global nucleotide substitution models for each gene. TVM=transversion model; GTR=general time reversal; TIM=transition model; TrN= Tamura-Nei; K=Kimura 2-Parameter; HKY=Hasegawa-Kishino-Yano; +G=gamma; +I+G=inverse gamma.

Table S3. Global phylogenetic comparisons by Shimodaira-Hasegawa (SH) and Shimodaira Approximately Unbiased (AU) tests for each gene. These tests compared the best unconstrained topology (gene tree), inclusive of all Orders, to gene trees constrained to an established supertree topology [15]. Decreased likelihood score (LH) for the constrained trees thus reflects differences between the gene trees and the supertree phylogeny. Columns are: LH, likelihood score; Δ(LH), difference in the likelihood scores between the best tree and remaining trees; SH, *P*-value for SH test; AU, *P*-value for AU test. When the PAUP software yielded multiple trees, α-values were adjusted accordingly using the Bonferroni correction. Asterisks denote significant *P*-values.

Gene	Tree	LH	Δ(LH)	SH	AU
Zan	Unconstrained #1	-167536.10	(best)		
	Unconstrained #2	-167546.20	10.11	0.48	0.19
	Unconstrained #3	-167548.49	12.41	0.43	0.09
	Constrained	-167794.75	258.67	<0.0001*	<0.0001*
Adam2	Unconstrained #1	-59665.91	(best)		
	Unconstrained #2	-59666.20	0.30	0.99	0.59
	Unconstrained #3	-59666.26	0.35	0.99	0.62
	Unconstrained #4	-59666.36	0.45	0.99	0.60
	Unconstrained #5	-59666.56	0.65	0.99	0.58
	Unconstrained #6-96	-59679.85, -59666.65	0.14, 0.95	0.65, 0.98	0.05, 0.60
	Constrained #1	-59754.19	88.28	0.0125	<0.0001*
	Constrained #2	-59754.46	88.55	0.011	0.0125
Zp2	Unconstrained #1	-78636.02	(best)		
	Unconstrained #2	-78636.08	0.06	0.90	0.29
	Unconstrained #3	-78636.08	0.06	0.90	0.29
	Unconstrained #4	-78638.16	2.14	0.74	0.56
	Unconstrained #5	-78638.22	2.20	0.74	0.18
	Unconstrained #6-10	-78643.44, -78638.22	2.20, 7.42	0.50, 0.74	0.03, 0.18
	Constrained #1	-78800.62	164.60	<0.0001*	<0.0001*
	Constrained #2	-78802.16	166.14	<0.0001*	<0.0001*
	Constrained #3	-78804.38	168.36	<0.0001*	<0.0001*

Gene	Tree	LH	Δ(LH)	SH	AU
Prm1	Unconstrained #1	-8269.71	(best)		
	Unconstrained #2	-8270.46	0.75	0.94	0.64
	Unconstrained #3	-8271.32	1.60	0.97	0.41
	Unconstrained #4	-8271.32	1.60	0.97	0.41
	Unconstrained #5	-8271.32	1.60	0.97	0.41
	Unconstrained #6-100	-8278.11, -8271.32	1.60, 8.40	0.53, 0.97	0.08, 0.63
	Constrained #1	-8298.10	28.39	0.15	0.16
	Constrained #2	-8299.81	30.09	0.13	0.06
Tecta	Unconstrained #1	-107633.05	(best)		
	Unconstrained #2	-107636.69	3.64	0.76	0.30
	Unconstrained #3	-107637.82	4.77	0.67	0.29
	Unconstrained #4	-107641.46	8.41	0.59	0.10
	Constrained	-111977.28	4344.23	<0.0001*	<0.0001*
Cytb	Constrained	-54959.91	(best)		
	Unconstrained #1	-55061.86	101.95	0.027	0.066
	Unconstrained #2	-55064.97	105.06	0.024	0.017
	Unconstrained #3	-55065.35	105.44	0.024	0.017
	Unconstrained #4	-55066.98	107.07	0.023	0.023

Table S4. Ordinal phylogenetic comparisons by Shimodaira-Hasegawa (SH) and Shimodaira Approximately Unbiased (AU) tests for each gene. These tests compared the best unconstrained topologies (gene trees) by individual Order to the corresponding ordinal topologies of the supertree [15]. Columns are defined as in Additional file 1: Table S3.

Gene	Order	Tree	LH	Δ(LH)	SH	AU
Zan	Afrotheria	Unconstrained	-17882.75	(best)		
		Constrained	-17913.45	30.70	0.01*	<0.0001*
	Carnivora	Unconstrained	-21418.57	(best)		
		Constrained	-21437.16	18.59	0.04*	0.01*
	Cetartiodactyla	Unconstrained	-21736.83	(best)		
		Constrained	-21736.88	0.00	1.00	0.50
	Chiroptera	Unconstrained	-19384.06	(best)		
		Constrained	-19470.36	86.30	0.0002*	<0.0001*
	Primates	Constrained	-31529.82	(best)		
		Unconstrained	-31536.62	6.80	0.26	0.21
	Rodentia	Unconstrained	-46874.33	(best)		
		Constrained	-46930.12	55.79	0.0004*	<0.0001*
Adam2	Carnivora	Unconstrained	-6049.88	(best)		
		Constrained	-6063.74	13.86	0.10	0.06
	Cetartiodactyla	Unconstrained #1	-7817.87	(best)		
		Unconstrained #2	-7818.04	0.17	0.83	0.46
		Unconstrained #3	-7818.05	0.19	0.83	0.53
		Unconstrained #4	-7818.22	0.36	0.80	0.46
		Unconstrained #5	-7833.54	15.67	0.09	0.006*
		Constrained	-7833.68	15.82	0.09	0.005*
	Chiroptera	Unconstrained	-8782.30	(best)		
		Constrained	-8782.30	0.00	1.00	0.50
	Primates	Unconstrained #1	-8733.41	(best)		
		Unconstrained #2	-8733.88	0.47	0.93	0.57
		Unconstrained #3	-8735.17	1.76	0.80	0.57
		Unconstrained #4	-8735.64	2.22	0.77	0.48
		Unconstrained #5	-8741.25	7.84	0.46	0.08
		Unconstrained #6-8	-8743.48, -8741.73	8.31, 10.07	0.34, 0.44	0.06, 0.05
		Constrained	-8744.00	10.58	0.21	0.04
	Rodentia	Unconstrained	-21032.05	(best)		
		Constrained	-21063.00	30.94	0.02*	0.02*

Gene	Order	Tree	LH	Δ(LH)	SH	AU
Zp2	Afrotheria	Unconstrained	-7596.55	(best)		
		Constrained	-7596.79	0.24	0.42	0.37
	Carnivora	Unconstrained	-9991.31	(best)		
		Constrained	-10040.10	48.79	<0.0001*	<0.0001*
	Cetartiodactyla	Unconstrained #1	-9595.23	(best)		
		Unconstrained #2	-9595.31	0.08	0.76	0.18
		Unconstrained #3	-9595.31	0.08	0.76	0.12
		Unconstrained #4	-9600.64	5.41	0.43	0.01
		Unconstrained #5	-9600.64	5.41	0.43	0.01
		Constrained	-9630.81	35.58	0.02	<0.0001*
	Chiroptera	Unconstrained	-14056.91	(best)		
		Constrained	-14057.02	0.11	0.39	0.36
	Primates	Unconstrained	-11410.61	(best)		
		Constrained	-11419.94	9.33	0.13	0.02*
	Rodentia	Unconstrained	-20091.50	(best)		
		Constrained	-20116.80	25.31	0.02*	<0.0001*
Prm1	Primates	Unconstrained #1	-1686.05	(best)		
		Unconstrained #2	-1686.05	0.00	0.98	0.73
		Unconstrained #3	-1686.05	0.00	0.98	0.60
		Unconstrained #4	-1686.95	0.90	0.75	0.48
		Unconstrained #5	-1686.95	0.90	0.75	0.48
		Unconstrained #6-12	-1690.62, -1686.95	0.90, 4.57	0.55, 0.87	0.008*, 0.48
		Constrained	-1698.53	12.48	0.24	0.01*
	Cetartiodactyla	Unconstrained #1	-1883.24	(best)		
		Unconstrained #2	-1883.24	0.00	0.93	0.61
		Unconstrained #3	-1883.24	0.00	0.93	0.61
		Unconstrained #4	-1883.31	0.07	0.65	0.47
		Unconstrained #5	-1883.31	0.07	0.65	0.45
		Unconstrained #6-9	-1883.31, -1883.31	0.07, 0.07	0.65, 0.65	0.44, 0.46
		Constrained	-1887.85	4.60	0.31	0.18
	Rodentia	Unconstrained #1	-1559.98	(best)		
		Unconstrained #2	-1560.01	0.02	0.92	0.64
		Unconstrained #3	-1560.11	0.13	0.92	0.34
		Unconstrained #4	-1560.11	0.13	0.92	0.34
		Unconstrained #5	-1560.13	0.15	0.86	0.23
		Unconstrained #6	-1560.13	0.15	0.86	0.23
		Constrained #1	-1556.00	0.01	0.64	0.55
		Constrained #2	-1561.40	1.42	0.38	0.14

Gene	Order	Tree	LH	Δ(LH)	SH	AU
Tecta	Afrotheria	Unconstrained #1	-16200.52	(best)		
		Unconstrained #2	-16201.03	0.51	0.68	0.43
		Constrained	-16222.23	21.71	0.03	0.01*
	Carnivora	Unconstrained	-15797.77	(best)		
		Constrained	-15805.02	7.25	0.08	0.04*
	Cetartiodactyla	Unconstrained	-17170.29	(best)		
		Constrained	-17170.29	0.00	1.00	0.50
	Chiroptera	Unconstrained	-18704.76	(best)		
		Constrained	-18704.76	0.00	1.00	0.50
	Primates	Constrained	-21291.28	(best)		
		Unconstrained #1	-21293.09	1.81	0.54	0.53
		Unconstrained #2	-21295.62	4.34	0.37	0.13
	Rodentia	Unconstrained	-27073.70	(best)		
		Constrained	-27079.36	5.65	0.26	0.24
Cytb	Afrotheria	Unconstrained	-5699.85	(best)		
		Constrained	-5706.19	6.34	0.15	0.13
	Carnivora	Unconstrained #1	-7308.92	(best)		
		Unconstrained #2	-7310.54	1.61	0.10	0.09
		Constrained	-7309.22	0.30	0.53	0.51
	Cetartiodactyla	Unconstrained	-8580.81	(best)		
		Constrained	-8585.75	4.93	0.19	0.17
	Chiroptera	Unconstrained	-6906.37	(best)		
		Constrained	-6906.77	0.40	0.45	0.44
	Primates	Unconstrained	-13314.48	(best)		
		Constrained	-13575.30	260.82	<0.0001*	<0.0001*
	Rodentia	Unconstrained	-11741.73	(best)		
		Constrained	-11743.11	1.38	0.47	0.47

Table S5. PAML summary results and likelihood ratio test statistics for models of variable selective pressure for each gene. Shown are log likelihood scores (LH) and parameter estimates for variable dN/dS models within the F3×4 codon frequency method. Models M7 (neutral, assumes different dN/dS for each branch) and M8 (selection, assumes different dN/dS for each branch and site) estimate beta-distributed, variable selective pressures, with selection at sites confirmed by Bayes Empirical Bayes statistical support. df = 1, degrees of freedom between the number of parameters used in Models M7 and M8 for each gene. The test statistic equals double the difference in log likelihood values between models being tested. Asterisks denote significant *P*-values.

Gene	Model	Parameter Estimates	LH	2(ΔLH)	P-value
Acr	M7: neutral	ω = 0.000 *f* = 0.200	-43297.80		
		ω = 0.000 *f* = 0.200			
		ω = 0.999 *f* = 0.200			
		ω = 0.999 *f* = 0.200			
		ω = 1.000 *f* = 0.200			
	M8: beta&ω>1	ω = 0.040 *f* = 0.175	-43154.80	143.00	<0.001*
		ω = 0.180 *f* = 0.175			
		ω = 0.460 *f* = 0.175			
		ω = 0.985 *f* = 0.175			
		ω = 0.999 *f* = 0.175			
		ω = 5.31 *f* = 0.129			
Adam2	M7: neutral	ω = 0.018 *f* = 0.200	-62335.28		
		ω = 0.190 *f* = 0.200			
		ω = 0.497 *f* = 0.200			
		ω = 0.806 *f* = 0.200			
		ω = 0.981 *f* = 0.200			
	M8: beta&ω>1	ω = 0.021 *f* = 0.175	-61919.46	831.64	<<0.001*
		ω = 0.170 *f* = 0.175			
		ω = 0.425 *f* = 0.175			
		ω = 0.716 *f* = 0.175			
		ω = 0.949 *f* = 0.175			
		ω = 2.344 *f* = 0.124			
Adam18	M7: neutral	ω = 0.010 *f* = 0.200	-40717.20		
		ω = 0.045 *f* = 0.200			
		ω = 0.129 *f* = 0.200			
		ω = 0.459 *f* = 0.200			
		ω = 0.992 *f* = 0.200			
	M8: beta&ω>1	ω = 0.018 *f* = 0.199	-40609.01	108.19	<0.001*
		ω = 0.194 *f* = 0.199			
		ω = 0.409 *f* = 0.199			
		ω = 0.600 *f* = 0.199			
		ω = 0.877 *f* = 0.199			
		ω = 12.05 *f* = 0.0028			

Gene	Model	Parameter Estimates	LH	2(ΔLH)	P-value
Adam32	M7: neutral	ω = 0.050 *f* = 0.200	-63856.11		
		ω = 0.166 *f* = 0.200			
		ω = 0.397 *f* = 0.200			
		ω = 0.995 *f* = 0.200			
		ω = 0.999 *f* = 0.200			
	M8: beta&ω>1	ω = 0.008 *f* = 0.192	-64233.10	1775.52	<<0.001*
		ω = 0.332 *f* = 0.192			
		ω = 0.863 *f* = 0.192			
		ω = 0.989 *f* = 0.192			
		ω = 0.999 *f* = 0.192			
		ω = 4.250 *f* = 0.0383			
Adgre1	M7: neutral	ω = 0.001 *f* = 0.200	-94872.77		
		ω = 0.052 *f* = 0.200			
		ω = 0.129 *f* = 0.200			
		ω = 0.899 *f* = 0.200			
		ω = 0.992 *f* = 0.200			
	M8: beta&ω>1	ω = 0.031 *f* = 0.196	-94131.50	1482.54	<<0.001*
		ω = 0.680 *f* = 0.196			
		ω = 0.682 *f* = 0.196			
		ω = 0.688 *f* = 0.196			
		ω = 0.998 *f* = 0.196			
		ω = 8.220 *f* = 0.0188			
C5ar1	M7: neutral	ω = 0.100 *f* = 0.200	-43909.81		
		ω = 0.100 *f* = 0.200			
		ω = 0.110 *f* = 0.200			
		ω = 0.980 *f* = 0.200			
		ω = 0.998 *f* = 0.200			
	M8: beta&ω>1	ω = 0.001 *f* = 0.187	-43901.10	17.42	<0.001*
		ω = 0.012 *f* = 0.187			
		ω = 0.280 *f* = 0.187			
		ω = 0.287 *f* = 0.187			
		ω = 0.289 *f* = 0.187			
		ω = 2.360 *f* = 0.0668			
Ccdc54	M7: neutral	ω = 0.001 *f* = 0.200	-31494.54		
		ω = 0.200 *f* = 0.200			
		ω = 0.679 *f* = 0.200			
		ω = 0.897 *f* = 0.200			
		ω = 0.999 *f* = 0.200			
	M8: beta&ω>1	ω = 0.007 *f* = 0.190	-31468.40	51.20	<0.001*
		ω = 0.198 *f* = 0.190			
		ω = 0.650 *f* = 0.190			
		ω = 0.986 *f* = 0.190			
		ω = 0.999 *f* = 0.190			
		ω = 4.870 *f* = 0.0487			

**Gene**	**Model**	**Parameter Estimates**	**LH**	**2(ΔLH)**	**P-value**
Ccl1	M7: neutral	$\omega = 0.001$ $f = 0.200$	-11310.00		
		$\omega = 0.008$ $f = 0.200$			
		$\omega = 0.644$ $f = 0.200$			
		$\omega = 0.891$ $f = 0.200$			
		$\omega = 0.999$ $f = 0.200$			
	M8: beta&ω>1	$\omega = 0.002$ $f = 0.157$	-11272.41	75.18	<0.001*
		$\omega = 0.229$ $f = 0.157$			
		$\omega = 0.230$ $f = 0.157$			
		$\omega = 0.234$ $f = 0.157$			
		$\omega = 0.998$ $f = 0.157$			
		$\omega = 3.670$ $f = 0.2152$			
Cfh	M7: neutral	$\omega = 0.001$ $f = 0.200$	-116628.42		
		$\omega = 0.003$ $f = 0.200$			
		$\omega = 0.999$ $f = 0.200$			
		$\omega = 0.999$ $f = 0.200$			
		$\omega = 0.999$ $f = 0.200$			
	M8: beta&ω>1	$\omega = 0.077$ $f = 0.191$	-115761.30	1734.24	<<0.001*
		$\omega = 0.078$ $f = 0.191$			
		$\omega = 0.078$ $f = 0.191$			
		$\omega = 0.078$ $f = 0.191$			
		$\omega = 0.078$ $f = 0.191$			
		$\omega = 2.344$ $f = 0.0433$			
Cr2	M7: neutral	$\omega = 0.030$ $f = 0.200$	-109467.52		
		$\omega = 0.040$ $f = 0.200$			
		$\omega = 0.988$ $f = 0.200$			
		$\omega = 0.990$ $f = 0.200$			
		$\omega = 0.999$ $f = 0.200$			
	M8: beta&ω>1	$\omega = 0.040$ $f = 0.199$	-109238.71	457.62	<<0.001*
		$\omega = 0.045$ $f = 0.199$			
		$\omega = 0.820$ $f = 0.199$			
		$\omega = 0.949$ $f = 0.199$			
		$\omega = 0.998$ $f = 0.199$			
		$\omega = 6.680$ $f = 0.0022$			
Crisp2	M7: neutral	$\omega = 0.001$ $f = 0.200$	-11014.23		
		$\omega = 0.002$ $f = 0.200$			
		$\omega = 0.004$ $f = 0.200$			
		$\omega = 0.998$ $f = 0.200$			
		$\omega = 0.999$ $f = 0.200$			
	M8: beta&ω>1	$\omega = 0.001$ $f = 0.198$	-10986.11	56.24	<0.001*
		$\omega = 0.270$ $f = 0.198$			
		$\omega = 0.271$ $f = 0.198$			
		$\omega = 0.274$ $f = 0.198$			
		$\omega = 0.274$ $f = 0.198$			
		$\omega = 2.280$ $f = 0.0104$			

Gene	Model	Parameter Estimates	LH	2(ΔLH)	P-value
Cytb	M7: neutral	ω = 0.001 *f* = 0.200	-51560.70		
		ω = 0.002 *f* = 0.200			
		ω = 0.014 *f* = 0.200			
		ω = 0.045 *f* = 0.200			
		ω = 0.139 *f* = 0.200			
	M8: beta&ω>1	ω = 0.0001 *f* = 0.200	-51560.70	0.00	>0.99
		ω = 0.003 *f* = 0.200			
		ω = 0.014 *f* = 0.200			
		ω = 0.045 *f* = 0.200			
		ω = 0.139 *f* = 0.200			
		ω = 2.450 *f* = 0.000			
Gpr50	M7: neutral	ω = 0.060 *f* = 0.200	-31879.94		
		ω = 0.062 *f* = 0.200			
		ω = 0.988 *f* = 0.200			
		ω = 0.998 *f* = 0.200			
		ω = 0.999 *f* = 0.200			
	M8: beta&ω>1	ω = 0.020 *f* = 0.186	-31852.30	55.28	<0.001*
		ω = 0.500 *f* = 0.186			
		ω = 0.503 *f* = 0.186			
		ω = 0.503 *f* = 0.186			
		ω = 0.504 *f* = 0.186			
		ω = 4.170 *f* = 0.070			
Izumo1	M7: neutral	ω = 0.999 *f* = 0.200	-40842.20		
		ω = 0.999 *f* = 0.200			
		ω = 0.999 *f* = 0.200			
		ω = 0.999 *f* = 0.200			
		ω = 0.999 *f* = 0.200			
	M8: beta&ω>1	ω = 0.951 *f* = 0.195	-40630.82	422.76	<<0.001*
		ω = 0.954 *f* = 0.195			
		ω = 0.948 *f* = 0.195			
		ω = 0.950 *f* = 0.195			
		ω = 0.999 *f* = 0.195			
		ω = 7.981 *f* = 0.0229			
Juno	M7: neutral	ω = 0.040 *f* = 0.200	-23276.81		
		ω = 0.044 *f* = 0.200			
		ω = 0.986 *f* = 0.200			
		ω = 0.999 *f* = 0.200			
		ω = 0.999 *f* = 0.200			
	M8: beta&ω>1	ω = 0.100 *f* = 0.181	-23264.00	25.62	<0.001*
		ω = 0.109 *f* = 0.181			
		ω = 0.141 *f* = 0.181			
		ω = 0.999 *f* = 0.181			
		ω = 0.999 *f* = 0.181			
		ω = 2.660 *f* = 0.0965			

Gene	Model	Parameter Estimates	LH	2(ΔLH)	P-value
Lelp1	M7: neutral	ω = 0.001 *f* = 0.200	-11674.21		
		ω = 0.010 *f* = 0.200			
		ω = 0.966 *f* = 0.200			
		ω = 0.999 *f* = 0.200			
		ω = 0.999 *f* = 0.200			
	M8: beta&ω>1	ω = 0.030 *f* = 0.189	-11589.90	168.62	<0.001*
		ω = 0.033 *f* = 0.189			
		ω = 0.998 *f* = 0.189			
		ω = 0.999 *f* = 0.189			
		ω = 0.999 *f* = 0.189			
		ω = 2.344 *f* = 0.0569			
Man2b1	M7: neutral	ω = 0.010 *f* = 0.200	-101596.43		
		ω = 0.011 *f* = 0.200			
		ω = 0.014 *f* = 0.200			
		ω = 0.806 *f* = 0.200			
		ω = 0.999 *f* = 0.200			
	M8: beta&ω>1	ω = 0.020 *f* = 0.199	-101261.70	669.46	<<0.001*
		ω = 0.021 *f* = 0.199			
		ω = 0.984 *f* = 0.199			
		ω = 0.997 *f* = 0.199			
		ω = 0.999 *f* = 0.199			
		ω = 9.950 *f* = 0.004			
Mgam	M7: neutral	ω = 0.001 *f* = 0.200	-117802.00		
		ω = 0.003 *f* = 0.200			
		ω = 0.988 *f* = 0.200			
		ω = 0.999 *f* = 0.200			
		ω = 0.999 *f* = 0.200			
	M8: beta&ω>1	ω = 0.001 *f* = 0.199	-117585.71	432.58	<<0.001*
		ω = 0.984 *f* = 0.199			
		ω = 0.999 *f* = 0.199			
		ω = 0.999 *f* = 0.199			
		ω = 0.999 *f* = 0.199			
		ω = 4.771 *f* = 0.0054			
Prdm9	M7: neutral	ω = 0.999 *f* = 0.200	-15698.10		
		ω = 0.999 *f* = 0.200			
		ω = 0.999 *f* = 0.200			
		ω = 0.999 *f* = 0.200			
		ω = 0.999 *f* = 0.200			
	M8: beta&ω>1	ω = 0.870 *f* = 0.182	-15655.61	84.98	<0.001*
		ω = 0.873 *f* = 0.182			
		ω = 0.999 *f* = 0.182			
		ω = 0.999 *f* = 0.182			
		ω = 0.999 *f* = 0.182			
		ω = 4.990 *f* = 0.092			

Gene	Model	Parameter Estimates	LH	2(ΔLH)	P-value
Prm1	M7: neutral	ω = 0.094 f_0 = 0.200	-7284.73		
		ω = 0.456 f_1 = 0.200			
		ω = 0.780 f_1 = 0.200			
		ω = 0.951 f_1 = 0.200			
		ω = 0.998 f_1 = 0.200			
	M8: beta&ω>1	ω = 0.095 f = 0.155	-7214.98	139.50	<0.001*
		ω = 0.383 f = 0.155			
		ω = 0.666 f = 0.155			
		ω = 0.878 f = 0.155			
		ω = 0.987 f = 0.155			
		ω = 4.026 f = 0.227			
Prm2	M7: neutral	ω = 0.020 f_0 = 0.200	-6864.00		
		ω = 0.021 f_1 = 0.200			
		ω = 0.640 f_1 = 0.200			
		ω = 0.999 f_1 = 0.200			
		ω = 0.999 f_1 = 0.200			
	M8: beta&ω>1	ω = 0.400 f = 0.159	-6861.51	4.98	<0.05*
		ω = 0.401 f = 0.159			
		ω = 0.404 f = 0.159			
		ω = 0.411 f = 0.159			
		ω = 0.414 f = 0.159			
		ω = 4.026 f = 0.2055			
S100a2	M7: neutral	ω = 0.150 f_0 = 0.200	-6569.11		
		ω = 0.154 f_1 = 0.200			
		ω = 0.162 f_1 = 0.200			
		ω = 0.998 f_1 = 0.200			
		ω = 0.999 f_1 = 0.200			
	M8: beta&ω>1	ω = 0.380 f = 0.191	-6565.66	6.90	0.05*
		ω = 0.381 f = 0.191			
		ω = 0.383 f = 0.191			
		ω = 0.384 f = 0.191			
		ω = 0.400 f = 0.191			
		ω = 4.026 f = 0.0472			
Slc6a5	M7: neutral	ω = 0.999 f_0 = 0.200	-45445.80		
		ω = 0.999 f_1 = 0.200			
		ω = 0.999 f_1 = 0.200			
		ω = 0.999 f_1 = 0.200			
		ω = 0.999 f_1 = 0.200			
	M8: beta&ω>1	ω = 0.999 f = 0.150	-45003.51	884.58	<<0.001*
		ω = 0.999 f = 0.150			
		ω = 0.999 f = 0.150			
		ω = 0.999 f = 0.150			
		ω = 0.999 f = 0.150			
		ω = 4.026 f = 0.2523			

Gene	Model	Parameter Estimates	LH	2(ΔLH)	P-value
Spaca6	M7: neutral	ω = 0.080 *f* = 0.200	-35863.30		
		ω = 0.081 *f* = 0.200			
		ω = 0.998 *f* = 0.200			
		ω = 0.999 *f* = 0.200			
		ω = 0.999 *f* = 0.200			
	M8: beta&ω>1	ω = 0.200 *f* = 0.196	-35654.41	417.78	<<0.001*
		ω = 0.202 *f* = 0.196			
		ω = 0.203 *f* = 0.196			
		ω = 0.999 *f* = 0.196			
		ω = 0.999 *f* = 0.196			
		ω = 6.900 *f* = 0.0208			
Spam1	M7: neutral	ω = 0.001 *f* = 0.200	-52071.73		
		ω = 0.002 *f* = 0.200			
		ω = 0.997 *f* = 0.200			
		ω = 0.999 *f* = 0.200			
		ω = 0.999 *f* = 0.200			
	M8: beta&ω>1	ω = 0.010 *f* = 0.197	-51946.00	251.46	<<0.001*
		ω = 0.014 *f* = 0.197			
		ω = 0.996 *f* = 0.197			
		ω = 0.999 *f* = 0.197			
		ω = 0.999 *f* = 0.197			
		ω = 4.440 *f* = 0.0143			
Spink2	M7: neutral	ω = 0.001 *f* = 0.200	-11256.72		
		ω = 0.002 *f* = 0.200			
		ω = 0.998 *f* = 0.200			
		ω = 0.999 *f* = 0.200			
		ω = 0.999 *f* = 0.200			
	M8: beta&ω>1	ω = 0.001 *f* = 0.186	-11144.01	225.42	<<0.001*
		ω = 0.986 *f* = 0.186			
		ω = 0.998 *f* = 0.186			
		ω = 0.999 *f* = 0.186			
		ω = 0.999 *f* = 0.186			
		ω = 7.690 *f* = 0.0683			
Sprr4	M7: neutral	ω = 0.000 *f* = 0.200	-7130.72		
		ω = 0.001 *f* = 0.200			
		ω = 0.998 *f* = 0.200			
		ω = 0.999 *f* = 0.200			
		ω = 0.999 *f* = 0.200			
	M8: beta&ω>1	ω = 0.220 *f* = 0.175	-7183.50	105.56	<0.001*
		ω = 0.222 *f* = 0.175			
		ω = 0.230 *f* = 0.175			
		ω = 0.999 *f* = 0.175			
		ω = 0.999 *f* = 0.175			
		ω = 5.781 *f* = 0.0848			

Gene	Model	Parameter Estimates	LH	2(ΔLH)	P-value
Tas1r2	M7: neutral	ω = 0.000 *f* = 0.200	-86368.64		
		ω = 0.001 *f* = 0.200			
		ω = 0.987 *f* = 0.200			
		ω = 0.993 *f* = 0.200			
		ω = 0.999 *f* = 0.200			
	M8: beta&ω>1	ω = 0.001 *f* = 0.199	-85916.51	904.26	<<0.001*
		ω = 0.002 *f* = 0.199			
		ω = 0.988 *f* = 0.199			
		ω = 0.999 *f* = 0.199			
		ω = 0.999 *f* = 0.199			
		ω = 6.970 *f* = 0.0043			
Tchhl1	M7: neutral	ω = 0.001 *f* = 0.200	-67157.11		
		ω = 0.776 *f* = 0.200			
		ω = 0.809 *f* = 0.200			
		ω = 0.998 *f* = 0.200			
		ω = 0.999 *f* = 0.200			
	M8: beta&ω>1	ω = 0.330 *f* = 0.183	-66967.40	379.42	<<0.001*
		ω = 0.332 *f* = 0.183			
		ω = 0.950 *f* = 0.183			
		ω = 0.951 *f* = 0.183			
		ω = 0.954 *f* = 0.183			
		ω = 6.621 *f* = 0.0837			
Tcn1	M7: neutral	ω = 0.000 *f* = 0.200	-67157.09		
		ω = 0.999 *f* = 0.200			
		ω = 0.999 *f* = 0.200			
		ω = 0.999 *f* = 0.200			
		ω = 0.999 *f* = 0.200			
	M8: beta&ω>1	ω = 0.330 *f* = 0.198	-66967.38	379.42	<<0.001*
		ω = 0.331 *f* = 0.198			
		ω = 0.950 *f* = 0.198			
		ω = 0.953 *f* = 0.198			
		ω = 0.954 *f* = 0.198			
		ω = 6.620 *f* = 0.0084			
Tcte1	M7: neutral	ω = 0.999 *f* = 0.200	-46523.80		
		ω = 0.999 *f* = 0.200			
		ω = 0.999 *f* = 0.200			
		ω = 0.999 *f* = 0.200			
		ω = 0.999 *f* = 0.200			
	M8: beta&ω>1	ω = 0.998 *f* = 0.167	-46208.63	630.34	<<0.001*
		ω = 0.999 *f* = 0.167			
		ω = 0.999 *f* = 0.167			
		ω = 0.999 *f* = 0.167			
		ω = 0.999 *f* = 0.167			
		ω = 6.900 *f* = 0.1644			

Gene	Model	Parameter Estimates	LH	2(ΔLH)	P-value
Tecta	M7: neutral	ω = 0.001 *f* = 0.200	-113678.56		
		ω = 0.040 *f* = 0.200			
		ω = 0.211 *f* = 0.200			
		ω = 0.554 *f* = 0.200			
		ω = 0.922 *f* = 0.200			
	M8: beta&ω>1	ω = 0.003 *f* = 0.185	-113326.98	703.16	<<0.001*
		ω = 0.041 *f* = 0.185			
		ω = 0.150 *f* = 0.185			
		ω = 0.358 *f* = 0.185			
		ω = 0.711 *f* = 0.185			
		ω = 1.790 *f* = 0.077			
Tectb	M7: neutral	ω = 0.999 *f* = 0.200	-22017.40		
		ω = 0.999 *f* = 0.200			
		ω = 0.999 *f* = 0.200			
		ω = 0.999 *f* = 0.200			
		ω = 0.999 *f* = 0.200			
	M8: beta&ω>1	ω = 0.001 *f* = 0.155	-21793.11	448.58	<<0.001*
		ω = 0.003 *f* = 0.155			
		ω = 0.998 *f* = 0.155			
		ω = 0.999 *f* = 0.155			
		ω = 0.999 *f* = 0.155			
		ω = 2.150 *f* = 0.2229			
Tex11	M7: neutral	ω = 0.000 *f* = 0.200	-72237.13		
		ω = 0.001 *f* = 0.200			
		ω = 0.984 *f* = 0.200			
		ω = 0.997 *f* = 0.200			
		ω = 0.999 *f* = 0.200			
	M8: beta&ω>1	ω = 0.000 *f* = 0.194	-71808.40	857.46	<<0.001*
		ω = 0.997 *f* = 0.194			
		ω = 0.998 *f* = 0.194			
		ω = 0.999 *f* = 0.194			
		ω = 0.999 *f* = 0.194			
		ω = 9.240 *f* = 0.0323			
Tex14	M7: neutral	ω = 0.000 *f* = 0.200	-110884.60		
		ω = 0.001 *f* = 0.200			
		ω = 0.999 *f* = 0.200			
		ω = 0.999 *f* = 0.200			
		ω = 0.999 *f* = 0.200			
	M8: beta&ω>1	ω = 0.000 *f* = 0.152	-110682.80	201.80	<<0.001*
		ω = 0.001 *f* = 0.152			
		ω = 0.999 *f* = 0.152			
		ω = 0.999 *f* = 0.152			
		ω = 0.999 *f* = 0.152			
		ω = 5.680 *f* = 0.240			

Gene	Model	Parameter Estimates	LH	2(ΔLH)	P-value
Tnp2	M7: neutral	ω = 0.001 *f* = 0.200	-13337.30		
		ω = 0.956 *f* = 0.200			
		ω = 0.987 *f* = 0.200			
		ω = 0.997 *f* = 0.200			
		ω = 0.999 *f* = 0.200			
	M8: beta&ω>1	ω = 0.000 *f* = 0.196	-13280.10	114.40	<0.001*
		ω = 0.988 *f* = 0.196			
		ω = 0.989 *f* = 0.196			
		ω = 0.995 *f* = 0.196			
		ω = 0.999 *f* = 0.196			
		ω = 3.801 *f* = 0.0186			
Usp26	M7: neutral	ω = 0.000 *f* = 0.200	-92606.61		
		ω = 0.999 *f* = 0.200			
		ω = 0.999 *f* = 0.200			
		ω = 0.999 *f* = 0.200			
		ω = 0.999 *f* = 0.200			
	M8: beta&ω>1	ω = 0.000 *f* = 0.195	-92258.82	695.58	<<0.001*
		ω = 0.999 *f* = 0.195			
		ω = 0.999 *f* = 0.195			
		ω = 0.999 *f* = 0.195			
		ω = 0.999 *f* = 0.195			
		ω = 7.160 *f* = 0.0253			
Wbp2nl	M7: neutral	ω = 0.000 *f* = 0.200	-31965.00		
		ω = 0.000 *f* = 0.200			
		ω = 0.976 *f* = 0.200			
		ω = 0.983 *f* = 0.200			
		ω = 0.999 *f* = 0.200			
	M8: beta&ω>1	ω = 0.001 *f* = 0.199	-31843.42	243.16	<<0.001*
		ω = 0.001 *f* = 0.199			
		ω = 0.988 *f* = 0.199			
		ω = 0.997 *f* = 0.199			
		ω = 0.999 *f* = 0.199			
		ω = 5.301 *f* = 0.0054			
Zp2	M7: neutral	ω = 0.233 *f* = 0.200	-58710.15		
		ω = 0.720 *f* = 0.200			
		ω = 0.940 *f* = 0.200			
		ω = 0.995 *f* = 0.200			
		ω = 0.999 *f* = 0.200			
	M8: beta&ω>1	ω = 0.163 *f* = 0.132	-58266.27	887.76	<<0.001*
		ω = 0.560 *f* = 0.132			
		ω = 0.873 *f* = 0.132			
		ω = 0.980 *f* = 0.132			
		ω = 0.999 *f* = 0.132			
		ω = 2.125 *f* = 0.338			

Gene	Model	Parameter Estimates	LH	2(ΔLH)	P-value
Zp3	M7: neutral	ω = 0.999 *f* = 0.200	-41521.90		
		ω = 0.999 *f* = 0.200			
		ω = 0.999 *f* = 0.200			
		ω = 0.999 *f* = 0.200			
		ω = 0.999 *f* = 0.200			
	M8: beta&ω>1	ω = 0.999 *f* = 0.168	-41382.44	278.92	<<0.001*
		ω = 0.999 *f* = 0.168			
		ω = 0.999 *f* = 0.168			
		ω = 0.999 *f* = 0.168			
		ω = 0.999 *f* = 0.168			
		ω = 6.320 *f* = 0.1591			
Zan	M7: neutral	ω = 0.147 *f* = 0.200	-163181.28		
		ω = 0.328 *f* = 0.200			
		ω = 0.673 *f* = 0.200			
		ω = 0.911 *f* = 0.200			
		ω = 0.995 *f* = 0.200			
	M8: beta&ω>1	ω = 0.058 *f* = 0.156	-162732.89	896.78	<<0.001*
		ω = 0.287 *f* = 0.156			
		ω = 0.562 *f* = 0.156			
		ω = 0.810 *f* = 0.156			
		ω = 0.972 *f* = 0.156			
		ω = 8.666 *f* = 0.220			

Table S6. Test statistics for ANOVA and post-hoc analysis of global gene divergence rates: Kruskal-Wallis H rank test (upper), ANOVA of divergence rate between and within Orders among all three genes collectively and by individual gene (middle), and Games-Howell post-hoc test comparing log-transformed divergence between genes (lower). df = degrees of freedom between Orders. Asterisks denote *P*<0.05.

Gene	Test statistic	df	*P*-value
Zan	71.313	8	<0.0001*
Adam2	73.283	8	<0.0001*
Zp2	65.848	8	<0.0001*
Prm1	46.161	5	<0.0001*
Tecta	68.146	8	<0.0001*
Cytb	71.768	8	<0.0001*
All	372.350	5	<0.0001*

Gene	Sum of squares		Degrees of freedom		Mean squares		F	*P*-value
	Between	Within	Between	Within	Between	Within		
Zan	0.938	0.299	8	99	0.117	0.003	38.83	<0.0001*
Adam2	2.623	0.633	8	92	0.328	0.007	47.69	<0.0001*
Zp2	0.722	0.553	8	106	0.090	0.005	17.30	<0.0001*
Prm1	4.369	0.819	8	50	0.874	0.016	53.35	<0.0001*
Tecta	0.998	0.557	8	95	0.125	0.006	21.26	<0.0001*
Cytb	12.112	5.469	8	101	1.514	0.054	27.96	<0.0001*
All	88.678	14.306	5	591	17.736	0.024	732.70	<0.0001*

Comparison	Mean difference	Std. Error	*P*-value	95% Confidence Interval	
				Lower bound	Upper bound
Zan vs Adam2	0.177	0.021	<0.0001*	0.117	0.236
Zan vs Zp2	0.075	0.014	<0.0001*	0.034	0.117
Zan vs Prm1	0.237	0.042	<0.0001*	0.113	0.361
Zan vs *Tecta*	0.265	0.016	<0.0001*	0.220	0.311
Zan vs *Cytb*	-0.826	0.015	<0.0001*	-0.868	-0.784
Adam2 vs Zp2	-0.101	0.020	<0.0001*	-0.160	-0.042
Adam2 vs Prm1	0.061	0.045	0.751	-0.070	0.191
Adam2 vs Tecta	-0.089	0.022	0.001*	-0.151	-0.027
Adam2 vs Cytb	-0.028	0.043	0.986	-0.153	0.097
Zp2 vs Prm1	0.162	0.042	0.004*	0.038	0.286
Zp2 vs Tecta	-0.190	0.016	<0.0001*	-0.235	-0.145
Zp2 vs Cytb	1.064	0.042	<0.0001*	0.940	1.187
Prm1 vs Tecta	-0.028	0.043	0.986	-0.153	0.097
Prm1 vs Cytb	0.061	0.045	0.751	-0.070	0.191
Tecta vs Cytb	-1.092	0.016	<0.0001*	-1.137	-1.046

Table S7. Games-Howell post-hoc test summary results for *Zan* ordinal comparisons of log-transformed divergence rate. Significant *P*-values are indicated by an asterisk.

Comparison	Mean difference	Std. Error	*P*-value	95% Confidence Interval	
				Lower	Upper
Afrotheria vs Perissodactyla	-0.008	0.048	1.000	-0.238	0.223
Afrotheria vs Chiroptera	-0.072	0.049	0.840	-0.299	0.156
Afrotheria vs Carnivora	-0.117	0.049	0.428	-0.345	0.111
Afrotheria vs Primates	-0.134	0.048	0.305	-0.364	0.096
Afrotheria vs Cetartiodactyla	-0.141	0.050	0.273	-0.367	0.085
Afrotheria vs Lagomorpha	-0.241	0.063	0.148	-0.580	0.098
Afrotheria vs Eulipotyphla	-0.337	0.089	0.162	-0.836	0.162
Afrotheria vs Rodentia	-0.315	0.050	0.010*	-0.540	-0.090
Perissodactyla vs Afrotheria	0.008	0.048	1.000	-0.223	0.238
Perissodactyla vs Chiroptera	-0.064	0.011	0.003*	-0.106	-0.022
Perissodactyla vs Carnivora	-0.109	0.010	<0.0001*	-0.146	-0.072
Perissodactyla vs Primates	-0.126	0.007	<0.0001*	-0.150	-0.103
Perissodactyla vs Cetartiodactyla	-0.133	0.014	<0.0001*	-0.180	-0.087
Perissodactyla vs Lagomorpha	-0.233	0.042	0.286	-1.568	1.101
Perissodactyla vs Eulipotyphla	-0.329	0.075	0.211	-1.044	0.386
Perissodactyla vs Rodentia	-0.307	0.015	<0.0001*	-0.360	-0.255
Chiroptera vs Afrotheria	0.072	0.049	0.840	-0.156	0.299
Chiroptera vs Perissodactyla	0.064	0.011	0.003*	0.022	0.106
Chiroptera vs Carnivora	-0.045	0.014	0.090	-0.094	0.004
Chiroptera vs Primates	-0.062	0.012	0.003*	-0.105	-0.019
Chiroptera vs Cetartiodactyla	-0.069	0.017	0.008*	-0.125	-0.013
Chiroptera vs Lagomorpha	-0.169	0.043	0.372	-1.223	0.889
Chiroptera vs Eulipoytyphla	-0.265	0.076	0.300	-0.959	0.428
Chiroptera vs Rodentia	-0.243	0.018	<0.0001*	-0.304	-0.182
Carnivora vs Afrotheria	0.117	0.049	0.428	-0.111	0.345
Carnivora vs Perissodactyla	0.109	0.010	<0.0001*	0.072	0.146
Carnivora vs Chiroptera	0.045	0.014	0.090	-0.004	0.094
Carnivora vs Primates	-0.017	0.011	0.826	-0.055	0.021
Carnivora vs Cetartiodactyla	-0.024	0.016	0.849	-0.078	0.030
Carnivora vs Lagomorpha	-0.124	0.043	0.501	-1.224	0.975
Carnivora vs Eulipotyphla	-0.220	0.076	0.399	-0.917	0.477
Carnivora vs Rodentia	-0.198	0.018	<0.0001*	-0.257	-0.139
Primates vs Afrotheria	0.134	0.048	0.305	-0.096	0.364
Primates vs Perissodactyla	0.126	0.007	<0.0001*	0.103	0.150
Primates vs Chiroptera	0.062	0.012	0.003*	0.019	0.105
Primates vs Carnivora	0.017	0.011	0.826	-0.021	0.055
Primates vs Cetartiodactyla	-0.007	0.014	1.000	-0.055	0.041
Primates vs Lagomorpha	-0.107	0.042	0.571	-1.383	1.169

**Comparison cont.**	**Mean difference**	**Std. Error**	***P*-value**	**95% Confidence Interval**	
				**Lower**	**Upper**
Primates vs Eulipotyphla	-0.203	0.075	0.447	-0.914	0.508
Primates vs Rodentia	-0.181	0.016	<0.0001*	-0.235	-0.127
Cetartiodactyla vs Afrotheria	0.141	0.050	0.273	-0.085	0.367
Cetartiodactyla vs Perissodactyla	0.133	0.014	<0.0001*	0.087	0.180
Cetartiodactyla vs Chiroptera	0.069	0.017	0.008*	0.013	0.125
Cetartiodactyla vs Carnivora	0.024	0.016	0.849	-0.030	0.078
Cetartiodactyla vs Primates	0.007	0.014	1.000	-0.041	0.055
Cetartiodactyla vs Lagomorpha	-0.100	0.043	0.602	-1.036	0.836
Cetartiodactyla vs Eulipotyphla	-0.196	0.076	0.470	-0.877	0.485
Cetartiodactyla vs Rodentia	-0.174	0.020	<0.0001*	-0.239	-0.109
Lagomorpha vs Afrotheria	0.241	0.063	0.148	-0.098	0.580
Lagomorpha vs Perissodactyla	0.233	0.042	0.286	-1.101	1.568
Lagomorpha vs Chiroptera	0.169	0.043	0.372	-0.889	1.228
Lagomorpha vs Carnivora	0.124	0.043	0.501	-0.975	1.224
Lagomorpha vs Primates	0.107	0.042	0.571	-1.169	1.383
Lagomorpha vs Cetartiodactyla	0.100	0.043	0.602	-0.836	1.036
Lagomorpha vs Eulipotyphla	-0.096	0.086	0.934	-0.671	0.480
Lagomorpha vs Rodentia	-0.074	0.044	0.756	-0.930	0.782
Eulipotyphla vs Afrotheria	0.337	0.089	0.162	-0.162	0.836
Eulipotyphla vs Perissodactyla	0.329	0.075	0.211	-0.386	1.044
Eulipotyphla vs Chiroptera	0.265	0.076	0.300	-0.428	0.959
Eulipotyphla vs Carnivora	0.220	0.076	0.399	-0.477	0.917
Eulipotyphla vs Primates	0.203	0.075	0.447	-0.508	0.914
Eulipotyphla vs Cetartiodactyla	0.196	0.076	0.470	-0.485	0.877
Eulipotyphla vs Lagomorpha	0.096	0.086	0.934	-0.480	0.671
Eulipotyphla vs Rodentia	0.022	0.076	1.000	-0.650	0.694
Rodentia vs Afrotheria	0.315	0.050	0.010*	0.090	0.540
Rodentia vs Perissodactyla	0.307	0.015	<0.0001*	0.255	0.360
Rodentia vs Chiroptera	0.243	0.018	<0.0001*	0.182	0.304
Rodentia vs Carnivora	0.198	0.018	<0.0001*	0.139	0.257
Rodentia vs Primates	0.181	0.016	<0.0001*	0.127	0.235
Rodentia vs Cetartiodactyla	0.174	0.020	<0.0001*	0.109	0.239
Rodentia vs Lagomorpha	0.074	0.044	0.756	-0.782	0.930
Rodentia vs Eulipotyphla	-0.022	0.076	1.000	-0.694	0.650

Table S8, Games-Howell post-hoc test summary results for *Adam2* ordinal comparisons of log-transformed divergence rate. Significant *P*-values are indicated by an asterisk.

Comparison	Mean difference	Std. Error	*P*-value	95% Confidence Interval	
				Lower	Upper
Afrotheria vs Perissodactyla	-0.0004	0.078	1.000	-0.621	0.620
Afrotheria vs Chiroptera	-0.108	0.080	0.865	-0.677	0.461
Afrotheria vs Carnivora	0.043	0.076	0.998	-0.631	0.717
Afrotheria vs Primates	-0.121	0.075	0.768	-0.815	0.573
Afrotheria vs Cetartiodactyla	-0.064	0.078	0.982	0.676	0.547
Afrotheria vs Lagomorpha	-0.223	0.085	0.417	-0.794	0.348
Afrotheria vs Eulipotyphla	-0.277	0.149	0.691	-2.058	1.503
Afrotheria vs Rodentia	-0.419	0.077	0.121	-1.052	0.215
Perissodactyla vs Afrotheria	0.0004	0.078	1.000	-0.620	0.621
Perissodactyla vs Chiroptera	-0.108	0.038	0.291	-0.289	0.073
Perissodactyla vs Carnivora	0.043	0.027	0.779	-0.277	0.363
Perissodactyla vs Primates	-0.121	0.025	0.277	-0.589	0.347
Perissodactyla vs Cetartiodactyla	-0.064	0.033	0.643	-0.253	0.125
Perissodactyla vs Lagomorpha	-0.223	0.047	0.231	-0.826	0.381
Perissodactyla vs Eulipotyphla	-0.277	0.131	0.656	-3.990	3.436
Perissodactyla vs Rodentia	-0.418	0.031	0.008*	-0.629	-0.208
Chiroptera vs Afrotheria	0.108	0.080	0.865	-0.461	0.677
Chiroptera vs Perissodactyla	0.108	0.038	0.291	-0.073	0.289
Chiroptera vs Carnivora	0.151	0.033	0.011*	0.029	0.273
Chiroptera vs Primates	-0.013	0.031	1.000	-0.133	0.107
Chiroptera vs Cetartiodactyla	0.044	0.038	0.959	-0.089	0.176
Chiroptera vs Lagomorpha	-0.115	0.051	0.544	-0.528	0.298
Chiroptera vs Eulipoytyphla	-0.169	0.132	0.869	-3.580	3.242
Chiroptera vs Rodentia	-0.311	0.036	<0.0001*	-0.438	-0.183
Carnivora vs Afrotheria	-0.043	0.076	0.998	-0.717	0.631
Carnivora vs Perissodactyla	-0.043	0.027	0.779	-0.363	0.277
Carnivora vs Chiroptera	-0.151	0.033	0.011*	-0.273	-0.029
Carnivora vs Primates	-0.164	0.016	<0.0001*	-0.218	-0.110
Carnivora vs Cetartiodactyla	-0.107	0.027	0.011*	-0.198	-0.017
Carnivora vs Lagomorpha	-0.266	0.043	0.214	-1.161	0.629
Carnivora vs Eulipotyphla	-0.320	0.129	0.589	-4.423	3.783
Carnivora vs Rodentia	-0.462	0.024	<0.0001*	-0.541	-0.382
Primates vs Afrotheria	0.121	0.075	0.768	-0.573	0.815
Primates vs Perissodactyla	0.121	0.025	0.277	-0.347	0.589
Primates vs Chiroptera	0.013	0.031	1.000	-0.107	0.133
Primates vs Carnivora	0.164	0.016	<0.0001*	-0.110	0.218
Primates vs Cetartiodactyla	0.057	0.025	0.402	-0.029	0.142
Primates vs Lagomorpha	-0.102	0.042	0.585	-1.204	1.001

Comparison cont.	Mean difference	Std. Error	*P*-value	95% Confidence Interval	
				Lower	Upper
Primates vs Eulipotyphla	-0.156	0.129	0.886	-4.369	4.057
Primates vs Rodentia	-0.297	0.022	<0.0001*	-0.371	-0.224
Cetartiodactyla vs Afrotheria	0.064	0.078	0.982	-0.547	0.676
Cetartiodactyla vs Perissodactyla	0.064	0.033	0.643	-0.125	0.253
Cetartiodactyla vs Chiroptera	-0.044	0.038	0.959	-0.176	0.089
Cetartiodactyla vs Carnivora	0.107	0.027	0.011*	0.017	0.198
Cetartiodactyla vs Primates	-0.057	0.025	0.402	-0.142	0.029
Cetartiodactyla vs Lagomorpha	-0.159	0.047	0.349	-0.688	0.371
Cetartiodactyla vs Eulipotyphla	-0.213	0.131	0.776	-3.934	3.509
Cetartiodactyla vs Rodentia	-0.354	0.031	<0.0001*	-0.456	-0.253
Lagomorpha vs Afrotheria	0.223	0.085	0.417	-0.348	0.794
Lagomorpha vs Perissodactyla	0.223	0.047	0.231	-0.381	0.826
Lagomorpha vs Chiroptera	0.115	0.051	0.544	-0.298	0.528
Lagomorpha vs Carnivora	0.266	0.043	0.214	-0.629	1.161
Lagomorpha vs Primates	0.102	0.042	0.585	-1.001	1.204
Lagomorpha vs Cetartiodactyla	0.159	0.047	0.349	-0.371	0.688
Lagomorpha vs Eulipotyphla	-0.054	0.135	0.999	-3.008	2.899
Lagomorpha vs Rodentia	-0.196	0.045	0.273	-0.821	0.430
Eulipotyphla vs Afrotheria	0.277	0.149	0.691	-1.503	2.058
Eulipotyphla vs Perissodactyla	0.277	0. 131	0.656	-3.436	3.990
Eulipotyphla vs Chiroptera	0.169	0. 132	0.869	-3.242	3.580
Eulipotyphla vs Carnivora	0.320	0. 129	0.589	-3.783	4.423
Eulipotyphla vs Primates	0.156	0. 129	0.886	-4.057	4.369
Eulipotyphla vs Cetartiodactyla	0.213	0. 131	0.776	-3.509	3.934
Eulipotyphla vs Lagomorpha	0.054	0.135	0.999	-2.899	3.008
Eulipotyphla vs Rodentia	-0.141	0.130	0.917	-4.007	3.724
Rodentia vs Afrotheria	0.419	0.077	0.121	-0.215	1.052
Rodentia vs Perissodactyla	0.418	0.031	0.008*	0.208	0.629
Rodentia vs Chiroptera	0.311	0.036	<0.0001*	0.183	0.438
Rodentia vs Carnivora	0.462	0.024	<0.0001*	0.382	0.541
Rodentia vs Primates	0.297	0.022	<0.0001*	0.224	0.371
Rodentia vs Cetartiodactyla	0.354	0.031	<0.0001*	0.253	0.456
Rodentia vs Lagomorpha	0.196	0.045	0.273	-0.430	0.821
Rodentia vs Eulipotyphla	0.141	0.130	0.917	-3.724	4.007

Table S9. Games-Howell post-hoc test summary results for *Zp2* Ordinal comparisons of log-transformed divergence rate. Significant *P*-values are indicated by an asterisk.

Comparison	Mean difference	Std. Error	*P*-value	95% Confidence Interval	
				Lower	Upper
Afrotheria vs Perissodactyla	0.103	0.049	0.565	-0.160	0.366
Afrotheria vs Chiroptera	-0.212	0.057	0.091	-0.455	0.300
Afrotheria vs Carnivora	-0.082	0.049	0.751	-0.343	0.179
Afrotheria vs Primates	0.008	0.050	1.000	-0.253	0.270
Afrotheria vs Cetartiodactyla	0.010	0.051	1.000	-0.245	0.265
Afrotheria vs Lagomorpha	-0.075	0.050	0.825	-0.333	0.183
Afrotheria vs Eulipotyphla	-0.130	0.058	0487	-0.391	0.132
Afrotheria vs Rodentia	-0.134	0.057	0.416	-0.375	0.107
Perissodactyla vs Afrotheria	-0.103	0.049	0.565	-0.366	0.160
Perissodactyla vs Chiroptera	-0.315	0.029	<0.0001*	-0.428	-0.203
Perissodactyla vs Carnivora	-0.185	0.008	<0.0001*	-0.212	-0.158
Perissodactyla vs Primates	-0.095	0.008	<0.0001*	-0.122	-0.067
Perissodactyla vs Cetartiodactyla	-0.093	0.013	<0.0001*	-0.138	-0.049
Perissodactyla vs Lagomorpha	-0.178	0.012	0.068	-0.403	-0.048
Perissodactyla vs Eulipotyphla	-0.233	0.032	0.079	-0.527	0.062
Perissodactyla vs Rodentia	-0.237	0.029	<0.0001*	-0.336	-0.137
Chiroptera vs Afrotheria	0.212	0.057	0.091	-0.030	0.455
Chiroptera vs Perissodactyla	0.315	0.029	<0.0001*	0.203	0.428
Chiroptera vs Carnivora	0.130	0.030	0.020*	0.018	0.243
Chiroptera vs Primates	0.221	0.030	<0.0001*	0.108	0.334
Chiroptera vs Cetartiodactyla	0.222	0.032	<0.0001*	0.107	0.337
Chiroptera vs Lagomorpha	0.138	0.031	0.019*	0.020	0.256
Chiroptera vs Eulipoytyphla	0.083	0.043	0.625	-0.110	0.276
Chiroptera vs Rodentia	0.079	0.041	0.594	-0.058	0.216
Carnivora vs Afrotheria	0.082	0.049	0.751	-0.179	0.343
Carnivora vs Perissodactyla	0.185	0.008	<0.0001*	0.158	0.212
Carnivora vs Chiroptera	-0.130	0.030	0.020*	-0.243	-0.018
Carnivora vs Primates	0.090	0.010	<0.0001*	0.061	0.120
Carnivora vs Cetartiodactyla	0.092	0.014	<0.0001*	0.046	0.138
Carnivora vs Lagomorpha	0.008	0.013	0.996	-0.138	0.153
Carnivora vs Eulipotyphla	-0.048	0.032	0.819	-0.330	0.235
Carnivora vs Rodentia	-0.052	0.029	0.692	-0.152	0.049
Primates vs Afrotheria	-0.008	0.050	1.000	-0.270	0.253
Primates vs Perissodactyla	0.095	0.008	<0.0001*	0.067	0.122
Primates vs Chiroptera	-0.221	0.030	<0.0001*	-0.334	-0.108
Primates vs Carnivora	-0.090	0.009	<0.0001*	-0.120	-0.061
Primates vs Cetartiodactyla	0.001	0.014	1.000	0.045	0.048
Primates vs Lagomorpha	-0.083	0.013	0.122	-0.221	0.055

Comparison cont.	Mean difference	Std. Error	*P*-value	95% Confidence Interval	
				Lower	Upper
Primates vs Eulipotyphla	-0.138	0.032	0.205	-0.419	0.143
Primates vs Rodentia	-0.142	0.029	0.002*	-0.242	-0.042
Cetartiodactyla vs Afrotheria	-0.010	0.051	1.000	-0.265	0.245
Cetartiodactyla vs Perissodactyla	0.093	0.013	<0.0001*	0.049	0.138
Cetartiodactyla vs Chiroptera	-0.222	0.032	<0.0001*	-0.337	-0.107
Cetartiodactyla vs Carnivora	-0.092	0.014	<0.0001*	-0.138	-0.046
Cetartiodactyla vs Primates	-0.001	0.014	1.000	-0.048	0.045
Cetartiodactyla vs Lagomorpha	-0.084	0.017	0.044*	-0.166	-0.003
Cetartiodactyla vs Eulipotyphla	-0.139	0.034	0.184	-0.383	0.104
Cetartiodactyla vs Rodentia	-0.143	0.031	0.003*	-0.248	-0.039
Lagomorpha vs Afrotheria	0.075	0.050	0.825	-0.183	0.333
Lagomorpha vs Perissodactyla	0.178	0.012	0.068	-0.048	0.403
Lagomorpha vs Chiroptera	-0.138	0.031	0.019*	-0.256	-0.020
Lagomorpha vs Carnivora	-0.008	0.013	0.996	-0.153	0.138
Lagomorpha vs Primates	0.083	0.013	0.122	-0.055	0.221
Lagomorpha vs Cetartiodactyla	0.084	0.017	0.044*	0.003	0.166
Lagomorpha vs Eulipotyphla	-0.055	0.034	0.759	-0.314	0.204
Lagomorpha vs Rodentia	-0.059	0.030	0.594	-0.166	0.048
Eulipotyphla vs Afrotheria	0.130	0.058	0.487	-0.132	0.391
Eulipotyphla vs Perissodactyla	0.233	0.032	0.079	-0.062	0.527
Eulipotyphla vs Chiroptera	-0.083	0.043	0.625	-0.276	0.110
Eulipotyphla vs Carnivora	0.048	0.032	0.819	-0.235	0.330
Eulipotyphla vs Primates	0.138	0.032	0.205	-0.143	0.419
Eulipotyphla vs Cetartiodactyla	0.139	0.034	0.184	-0.104	0.383
Eulipotyphla vs Lagomorpha	0.055	0.034	0.759	-0.204	0.314
Eulipotyphla vs Rodentia	-0.004	0.042	1.000	-0.194	0.185
Rodentia vs Afrotheria	0.134	0.057	0.416	-0.107	0.375
Rodentia vs Perissodactyla	0.237	0.029	<0.0001*	0.137	0.336
Rodentia vs Chiroptera	-0.079	0.041	0.594	-0.216	0.058
Rodentia vs Carnivora	0.052	0.029	0.692	-0.049	0.152
Rodentia vs Primates	0.142	0.029	0.002*	0.042	0.242
Rodentia vs Cetartiodactyla	0.143	0.031	0.003*	0.039	0.248
Rodentia vs Lagomorpha	0.059	0.030	0.594	-0.048	0.166
Rodentia vs Eulipotyphla	0.004	0.042	1.000	-0.185	0.194

Table S10. Ryan's Q post-hoc test summary results for *Prm1* ordinal comparisons of divergence rate. Significant *P*-values are indicated by an asterisk.

Comparison	Mean difference	Std. Error	*P*-value	95% Confidence Interval	
				Lower	Upper
Perissodactyla vs Chiroptera	-0.021	0.025	0.999	0.098	0.056
Perissodactyla vs Carnivora	0.175	0.025	<0.0001*	0.252	0.098
Perissodactyla vs Primates	0.114	0.019	<0.0001*	0.057	0.171
Perissodactyla vs Cetartiodactyla	0.086	0.019	<0.0001*	0.143	0.028
Perissodactyla vs Rodentia	0.145	0.020	<0.0001*	0.085	0.028
Chiroptera vs Perissodactyla	0.021	0.025	0.999	-0.056	0.098
Chiroptera vs Carnivora	0.154	0.025	<0.0001*	-0.023	-0.076
Chiroptera vs Primates	0.135	0.019	<0.0001*	0.078	0.192
Chiroptera vs Cetartiodactyla	0.065	0.019	0.017*	-0.122	0.007
Chiroptera vs Rodentia	0.166	0.020	<0.0001*	0.106	0.226
Carnivora vs Perissodactyla	0.175	0.025	<0.0001*	0.098	0.252
Carnivora vs Chiroptera	0.154	0.025	<0.0001*	0.076	0.231
Carnivora vs Primates	0.289	0.019	<0.0001*	0.231	0.346
Carnivora vs Cetartiodactyla	0.089	0.019	<0.0001*	0.031	0.147
Carnivora vs Rodentia	0.320	0.020	<0.0001*	0.260	0.380
Primates vs Perissodactyla	0.114	0.019	<0.0001*	-0.171	0.057
Primates vs Chiroptera	-0.135	0.019	<0.0001*	-0.192	0.078
Primates vs Carnivora	-0.289	0.019	<0.0001*	-0.346	0.231
Primates vs Cetartiodactyla	-0.199	0.008	<0.0001*	0.224	0.175
Primates vs Rodentia	0.031	0.010	0.028*	0.002	0.061
Cetartiodactyla vs Perissodactyla	0.086	0.019	<0.0001*	0.028	0.143
Cetartiodactyla vs Chiroptera	0.065	0.019	0.017*	0.007	0.122
Cetartiodactyla vs Carnivora	-0.089	0.019	<0.0001*	-0.147	-0.031
Cetartiodactyla vs Primates	0.199	0.008	<0.0001*	0.175	0.224
Cetartiodactyla vs Rodentia	0.231	0.010	<0.0001*	0.200	0.261
Rodentia vs Perissodactyla	-0.145	0.020	<0.0001*	-0.205	-0.085
Rodentia vs Chiroptera	-0.166	0.020	<0.0001*	-0.226	-0.106
Rodentia vs Carnivora	-0.320	0.020	<0.0001*	-0.380	-0.260
Rodentia vs Primates	-0.031	0.010	0.028*	-0.061	-0.002
Rodentia vs Cetartiodactyla	-0.231	0.010	<0.0001*	-0.261	-0.200

Table S11. Games-Howell post-hoc test summary results for *Tecta* Ordinal comparisons of log-transformed divergence rate. Significant *P*-values are indicated by an asterisk.

Comparison	Mean difference	Std. Error	*P*-value	95% Confidence Interval	
				Lower	Upper
Afrotheria vs Perissodactyla	0.149	0.035	0.061	-0.007	0.304
Afrotheria vs Chiroptera	-0.134	0.060	0.437	-0.350	0.082
Afrotheria vs Carnivora	-0.035	0.036	0.976	-0.190	0.119
Afrotheria vs Primates	0.069	0.037	0.634	-0.084	0.223
Afrotheria vs Cetartiodactyla	-0.074	0.037	0.572	0.228	0.079
Afrotheria vs Lagomorpha	-0.040	0.052	0.990	-0.388	0.308
Afrotheria vs Eulipotyphla	-0.219	0.043	0.018*	-0.399	-0.038
Afrotheria vs Rodentia	-0.178	0.040	0.022*	-0.333	-0.023
Perissodactyla vs Afrotheria	-0.149	0.035	0.061	-0.304	0.007
Perissodactyla vs Chiroptera	-0.282	0.049	0.005*	-0.476	-0.089
Perissodactyla vs Carnivora	-0.184	0.010	<0.0001*	-0.221	-0.148
Perissodactyla vs Primates	-0.079	0.011	<0.0001*	-0.117	-0.041
Perissodactyla vs Cetartiodactyla	-0.223	0.012	<0.0001*	-0.265	-0.181
Perissodactyla vs Lagomorpha	-0.189	0.039	0.332	-1.460	1.082
Perissodactyla vs Eulipotyphla	-0.367	0.026	0.022*	-0.609	-0.125
Perissodactyla vs Rodentia	-0.326	0.021	<0.0001*	-0.399	-0.254
Chiroptera vs Afrotheria	-0.134	0.060	0.437	-0.082	0.350
Chiroptera vs Perissodactyla	0.282	0.049	0.005*	0.089	0.476
Chiroptera vs Carnivora	0.098	0.050	0.588	-0.095	0.292
Chiroptera vs Primates	0.203	0.050	0.038*	0.010	0.397
Chiroptera vs Cetartiodactyla	0.059	0.050	0.943	-0.134	0.253
Chiroptera vs Lagomorpha	0.094	0.063	0.823	-0.203	0.391
Chiroptera vs Eulipoytyphla	-0.085	0.055	0.819	-0.295	0.125
Chiroptera vs Rodentia	-0.044	0.053	0.993	-0.241	0.152
Carnivora vs Afrotheria	0.035	0.036	0.976	-0.119	0.190
Carnivora vs Perissodactyla	0.184	0.010	<0.0001*	0.148	0.221
Carnivora vs Chiroptera	-0.098	0.050	0.588	-0.292	0.095
Carnivora vs Primates	0.105	0.014	<0.0001*	0.057	0.153
Carnivora vs Cetartiodactyla	-0.039	0.015	0.237	0.089	0.011
Carnivora vs Lagomorpha	-0.005	0.040	1.000	-1.023	1.014
Carnivora vs Eulipotyphla	-0.183	0.028	0.065	-0.387	0.021
Carnivora vs Rodentia	-0.142	0.023	<0.0001*	-0.219	-0.065
Primates vs Afrotheria	-0.069	0.037	0.634	-0.223	0.084
Primates vs Perissodactyla	0.079	0.011	<0.0001*	0.041	0.117
Primates vs Chiroptera	-0.203	0.050	0.038*	-0.397	-0.010
Primates vs Carnivora	-0.105	0.014	<0.0001*	-0.153	-0.057
Primates vs Cetartiodactyla	-0.144	0.016	<0.0001*	-0.196	-0.092
Primates vs Lagomorpha	-0.109	0.041	0.531	-1.061	0.842

**Comparison cont.**	**Mean difference**	**Std. Error**	***P*-value**	**95% Confidence Interval**	
				**Lower**	**Upper**
Primates vs Eulipotyphla	-0.288	0.028	0.018*	-0.483	-0.093
Primates vs Rodentia	-0.247	0.023	<0.0001*	-0.326	-0.169
Cetartiodactyla vs Afrotheria	0.074	0.037	0.572	-0.079	0.228
Cetartiodactyla vs Perissodactyla	0.223	0.012	<0.0001*	0.181	0.265
Cetartiodactyla vs Chiroptera	-0.059	0.050	0.943	-0.253	0.134
Cetartiodactyla vs Carnivora	0.039	0.015	0.237	-0.011	0.089
Cetartiodactyla vs Primates	0.144	0.016	<0.0001*	-0.092	0.196
Cetartiodactyla vs Lagomorpha	0.034	0.041	0.969	-0.880	0.948
Cetartiodactyla vs Eulipotyphla	-0.144	0.028	0.102	-0.335	0.046
Cetartiodactyla vs Rodentia	-0.103	0.024	0.004*	-0.183	-0.024
Lagomorpha vs Afrotheria	0.040	0.052	0.990	-0.308	0.388
Lagomorpha vs Perissodactyla	0.189	0.039	0.332	-1.082	1.460
Lagomorpha vs Chiroptera	-0.094	0.063	0.823	-0.391	0.203
Lagomorpha vs Carnivora	0.005	0.040	1.000	-1.014	1.023
Lagomorpha vs Primates	0.109	0.041	0.531	-0.842	1.061
Lagomorpha vs Cetartiodactyla	-0.034	0.041	0.969	-0.948	0.880
Lagomorpha vs Eulipotyphla	-0.179	0.147	0.280	-0.660	0.303
Lagomorpha vs Rodentia	-0.138	0.044	0.404	-0.700	0.424
Eulipotyphla vs Afrotheria	0.219	0.043	0.018*	0.038	0.399
Eulipotyphla vs Perissodactyla	0.367	0.026	0.022*	0.125	0.609
Eulipotyphla vs Chiroptera	0.085	0.055	0.819	-0.125	0.295
Eulipotyphla vs Carnivora	0.183	0.028	0.065	-0.021	0.387
Eulipotyphla vs Primates	0.288	0.028	0.018*	0.093	0.483
Eulipotyphla vs Cetartiodactyla	0.144	0.028	0.102	-0.046	0.335
Eulipotyphla vs Lagomorpha	0.179	0.047	0.280	-0.303	0.660
Eulipotyphla vs Rodentia	0.041	0.033	0.916	-0.118	0.199
Rodentia vs Afrotheria	0.178	0.040	0.022*	0.023	0.333
Rodentia vs Perissodactyla	0.326	0.021	<0.0001*	0.254	0.399
Rodentia vs Chiroptera	0.044	0.053	0.993	-0.152	0.241
Rodentia vs Carnivora	0.142	0.023	<0.0001*	0.065	0.219
Rodentia vs Primates	0.247	0.023	<0.0001*	0.169	0.326
Rodentia vs Cetartiodactyla	0.103	0.024	0.004*	0.024	0.183
Rodentia vs Lagomorpha	0.138	0.044	0.404	-0.424	0.700
Rodentia vs Eulipotyphla	0.041	0.033	0.916	-0.199	0.118

Table S12. Games-Howell post-hoc test summary results for *Cytb* Ordinal comparisons of divergence rate. Significant *P*-values are indicated by an asterisk.

Comparison	Mean difference	Std. Error	*P*-value	95% Confidence Interval	
				Lower	Upper
Afrotheria vs Perissodactyla	-0.231	0.112	0.554	-0.702	0.240
Afrotheria vs Chiroptera	-0.484	0.128	0.049*	-0.966	-0.002
Afrotheria vs Carnivora	-0.352	0.116	0.182	-0.821	0.118
Afrotheria vs Primates	-0.773	0.122	0.002*	-1.243	-0.302
Afrotheria vs Cetartiodactyla	-0.437	0.119	0.072	-0.907	0.032
Afrotheria vs Lagomorpha	0.101	0.111	0.985	-0.371	0.572
Afrotheria vs Eulipotyphla	-0.181	0.368	0.999	-3.061	2.699
Afrotheria vs Rodentia	0.158	0.121	0.906	-0.312	0.629
Perissodactyla vs Afrotheria	0.231	0.112	0.554	-0.240	0.702
Perissodactyla vs Chiroptera	-0.253	0.066	0.050	-0.506	0.0001
Perissodactyla vs Carnivora	-0.121	0.036	0.071	-0.248	0.007
Perissodactyla vs Primates	-0.542	0.051	<0.0001*	-0.712	-0.371
Perissodactyla vs Cetartiodactyla	-0.206	0.044	0.003*	-0.356	-0.056
Perissodactyla vs Lagomorpha	0.332	0.016	0.001*	0.235	0.429
Perissodactyla vs Eulipotyphla	0.050	0.352	1.000	-3.299	3.400
Perissodactyla vs Rodentia	0.389	0.050	<0.0001*	0.218	0.560
Chiroptera vs Afrotheria	0.484	0.128	0.049*	0.002	0.966
Chiroptera vs Perissodactyla	0.253	0.066	0.050	-0.0001	0.506
Chiroptera vs Carnivora	0132	0.071	0.652	-0.128	0.392
Chiroptera vs Primates	-0.289	0.080	0.037*	-0.566	-0.011
Chiroptera vs Cetartiodactyla	0.047	0.076	0.999	-0.221	0.315
Chiroptera vs Lagomorpha	0.585	0.064	<0.0001*	0.333	0.837
Chiroptera vs Eulipoytyphla	0.303	0.357	0.977	-2.869	3.476
Chiroptera vs Rodentia	0.642	0.079	<0.0001*	0.366	0.919
Carnivora vs Afrotheria	0.352	0.116	0.182	-0.118	0.821
Carnivora vs Perissodactyla	0.121	0.036	0.071	-0.007	0.248
Carnivora vs Chiroptera	-0.132	0.071	0.652	-0.393	0.128
Carnivora vs Primates	-0.421	0.059	<0.0001*	-0.613	-0.229
Carnivora vs Cetartiodactyla	-0.085	0.052	0.778	-0.259	0.088
Carnivora vs Lagomorpha	0.453	0.032	<0.0001*	0.334	0.571
Carnivora vs Eulipotyphla	0.171	0.353	0.999	-3.139	3.480
Carnivora vs Rodentia	0.510	0.057	<0.0001*	0.319	0.701
Primates vs Afrotheria	0.773	0.122	0.002*	0.302	1.243
Primates vs Perissodactyla	0.542	0.051	<0.0001*	0.371	0.712
Primates vs Chiroptera	0.289	0.080	0.037*	0.011	0.566
Primates vs Carnivora	0.421	0.059	<0.0001*	0.229	0.613
Primates vs Cetartiodactyla	0.336	0.064	<0.0001*	0.128	0.543
Primates vs Lagomorpha	0.874	0.049	<0.0001*	0.710	1.038

Comparison cont.	Mean difference	Std. Error	*P*-value	95% Confidence Interval	
				Lower	Upper
Primates vs Eulipotyphla	0.592	0.355	0.749	-2.654	3.837
Primates vs Rodentia	0.931	0.068	<0.0001*	0.710	1.152
Cetartiodactyla vs Afrotheria	0.437	0.119	0.072	-0.032	0.907
Cetartiodactyla vs Perissodactyla	0.206	0.044	0.003*	0.056	0.356
Cetartiodactyla vs Chiroptera	-0.047	0.076	0.999	-0.315	0.221
Cetartiodactyla vs Carnivora	0.085	0.052	0.778	-0.088	0.259
Cetartiodactyla vs Primates	-0.336	0.064	<0.0001*	-0.543	-0.128
Cetartiodactyla vs Lagomorpha	0.538	0.041	<0.0001*	0.395	0.681
Cetartiodactyla vs Eulipotyphla	0.256	0.354	0.990	-3.023	3.535
Cetartiodactyla vs Rodentia	0.595	0.062	<0.0001*	0.390	0.801
Lagomorpha vs Afrotheria	-0.101	0.111	0.985	-0.573	0.371
Lagomorpha vs Perissodactyla	-0.332	0.016	0.001*	-0.429	-0.235
Lagomorpha vs Chiroptera	-0.585	0.064	<0.0001*	-0.837	-0.333
Lagomorpha vs Carnivora	-0.453	0.032	<0.0001*	-0.571	-0.334
Lagomorpha vs Primates	-0.874	0.049	<0.0001*	-1.038	-0.710
Lagomorpha vs Cetartiodactyla	-0.538	0.041	<0.0001*	-0.681	-0.395
Lagomorpha vs Eulipotyphla	-0.282	0.351	0.982	-3.643	3.079
Lagomorpha vs Rodentia	0.057	0.047	0.942	-0.108	0.223
Eulipotyphla vs Afrotheria	0.181	0.368	0.999	-2.699	3.061
Eulipotyphla vs Perissodactyla	-0.502	0.352	1.000	-3.400	3.299
Eulipotyphla vs Chiroptera	-0.303	0.357	0.977	-3.476	2.870
Eulipotyphla vs Carnivora	-0.171	0.353	0.999	-3.480	3.139
Eulipotyphla vs Primates	-0.591	0.355	0.749	-3.837	2.654
Eulipotyphla vs Cetartiodactyla	-0.256	0.354	0.990	-3.535	3.023
Eulipotyphla vs Lagomorpha	0.282	0.351	0.982	-3.079	3.643
Eulipotyphla vs Rodentia	0.339	0.354	0.960	-2.914	3.593
Rodentia vs Afrotheria	-0.158	0.121	0.906	-0.629	0.312
Rodentia vs Perissodactyla	-0.389	0.050	<0.0001*	-0.560	-0.218
Rodentia vs Chiroptera	-0.642	0.080	<0.0001*	-0.919	-0.366
Rodentia vs Carnivora	-0.510	0.057	<0.0001*	-0.701	-0.319
Rodentia vs Primates	-0.931	0.068	<0.0001*	-1.152	-0.710
Rodentia vs Cetartiodactyla	-0.595	0.062	<0.0001*	-0.801	-0.390
Rodentia vs Lagomorpha	-0.057	0.047	0.942	-0.223	0.108
Rodentia vs Eulipotyphla	-0.339	0.354	0.960	-3.593	2.914
