# Supplementary material for: Rapid divergence of a gamete recognition gene promoted macroevolution of Eutheria
Source: Genome Biol. 2022 Jul 11;23:155. doi: 10.1186/s13059-022-02721-y (PMC9275260; doi:10.1186/s13059-022-02721-y)
Supplement: Supplementary file 2 — Additional file 2. Comprises supplemental figures S1-S3 illustrating linear comparison of mouse and opossum Zan loci, a zonadhesin protein sequence tree, and Zan divergence rate ranked by species, respectively. [file 13059_2022_2721_MOESM2_ESM.pdf]

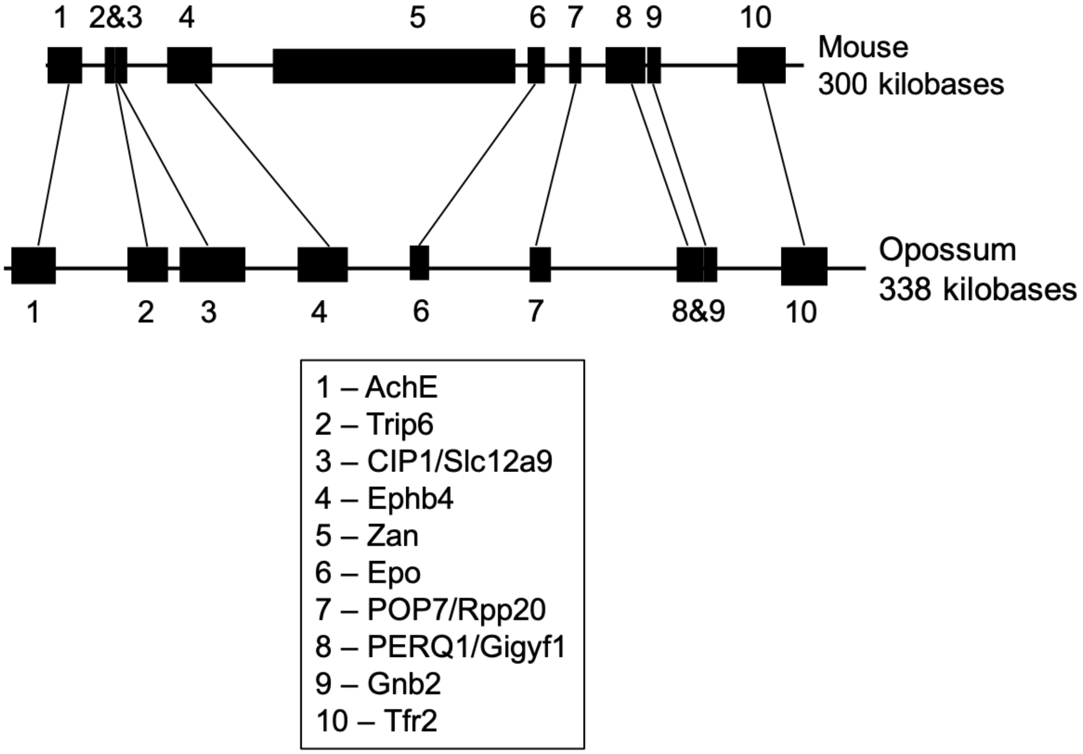

Fig. S1. Linear comparison of mouse and opossum loci spanning *AchE* to *Tfr2*, showing absence of *Zan* between *Epo* and *Ephb4*.

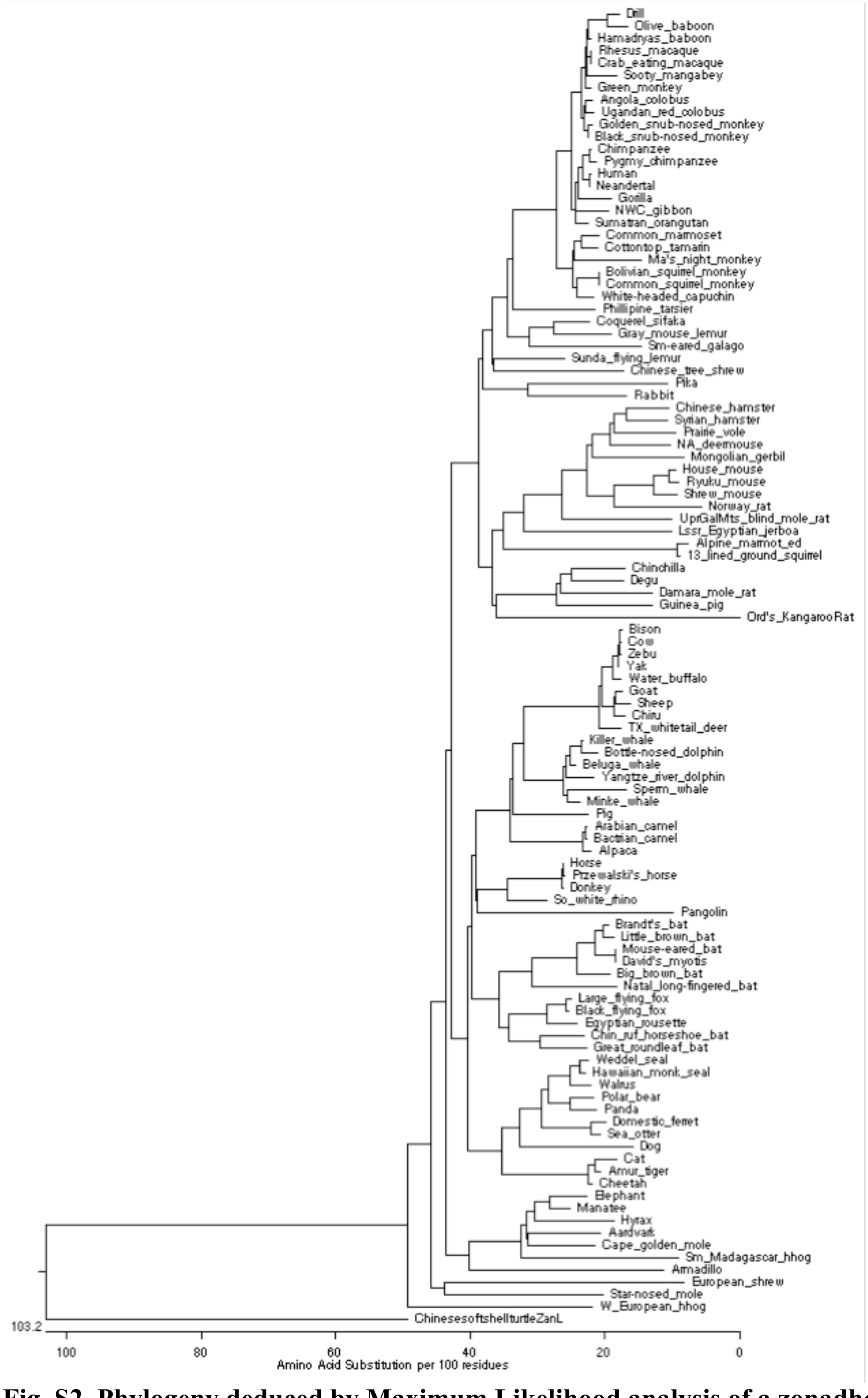

Fig. S2. Phylogeny deduced by Maximum Likelihood analysis of a zonadhesin protein sequence alignment.

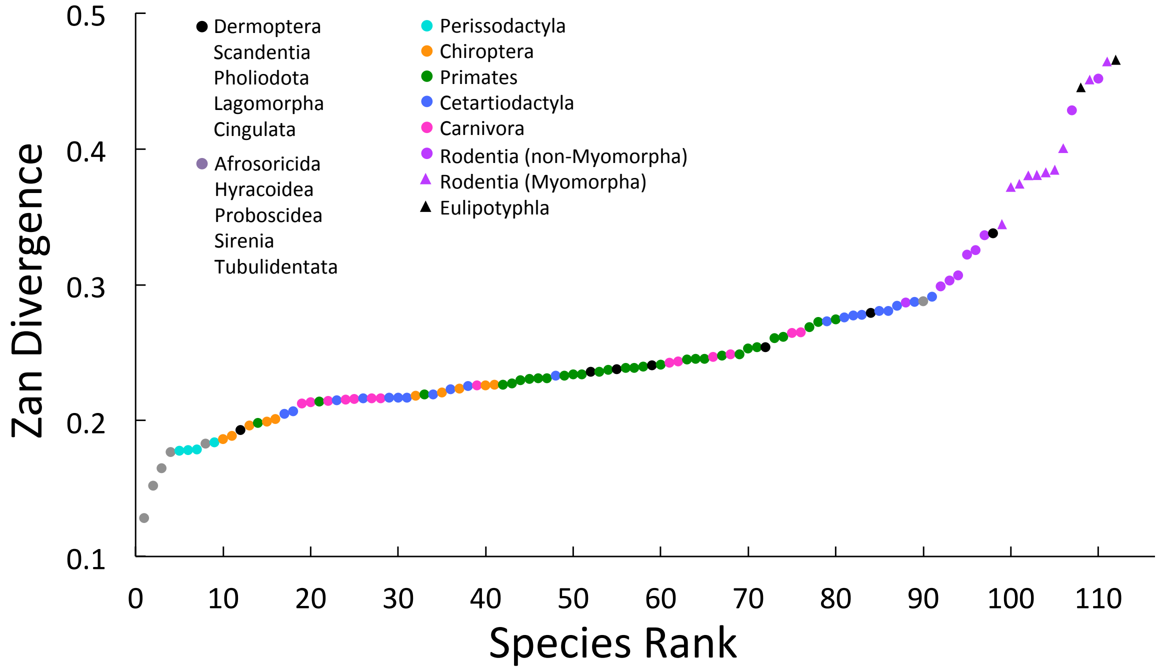

Fig. S3. Rank rate plot of *Zan* DNA sequence divergence. Shown are divergence rates calculated by branch length analysis from nodes representing the origins of the species' respective Superorders (Euarchontoglires, 98.9 MYA; Laurasiatheria, 98.9 MYA; Afrotheria; 101.3 MYA) ranked from lowest divergence rate (1) to highest (112). Note the relatively similar divergence rates among species from most Orders, including Primates, Artiodactyla, Perissodactyla, Cetacea, Chiroptera, and Carnivora, and the accelerated divergence rates among members of the Myomorpha Suborder of Rodentia.
