# Supplementary material for: Rapid divergence of a gamete recognition gene promoted macroevolution of Eutheria
Source: Genome Biol. 2022 Jul 11;23:155. doi: 10.1186/s13059-022-02721-y (PMC9275260; doi:10.1186/s13059-022-02721-y)
Supplement: Supplementary file 3 — Additional file 3. Presents full results narratives for topological comparisons of the Adam2, Zp2, Prm1, Tecta, and Cytb gene trees, as well as the DRYAD DOI and URL for shared data, including accession numbers for all database sequence files. [file 13059_2022_2721_MOESM3_ESM.pdf]

Supplemental Results

Tree topology comparisons

Parsimony analysis of aligned *Adam2* sequences yielded 96 candidate trees, each about equally likely, and two candidate trees constrained to the supertree topology, each also about equally likely. The global topology of the best *Adam2* tree differed ($P<0.0001$, AU test) from only one of the two supertree-constrained topologies (S3-S4 Tables). Single best *Adam2* ordinal topologies for Carnivora and Chiroptera did not differ from their respective supertree topologies. Among the more speciose Orders represented in the *Adam2* dataset (Rodentia, Primates, Cetartiodactyla), a single best Rodentia ordinal topology differed from its supertree topology ($P<0.02$, AU test), but none of eight equally likely Primates topologies differed from its supertree, and among five Cetartiodactyla topologies, four were about equally likely, with the best topology differing both from the least likely *Adam2* topology and from its supertree topology ($P<0.006$, AU test). Altogether, the numerous global and ordinal *Adam2* candidate topologies and absence of topology differences for Orders not fully resolved in the supertree (Carnivora, Chiroptera) diminished the utility of *Adam2* for inferring mammalian phylogeny, especially at terminal branches. Thus, in contrast to *Zan*, molecular evolution of *Adam2* has not occurred in strict concordance with Eutherian species divergence.

For *Zp2*, parsimony analysis yielded 10 candidate trees, each about equally likely, and three candidate supertree-constrained topologies, each also about equally likely. The global topology of the best *Zp2* tree differed ($P<0.0001$, AU test) from all three of the constrained topologies (S3-S4 Tables). Single best *Zp2* ordinal topologies for Carnivora, Primates, and Rodentia, as well as the best of five topologies for Cetartiodactyla, also each differed from their respective supertree-constrained topologies ($P<0.02$, AU test), but single best topologies for

Superorder Afrotheria and Order Chiroptera did not. Consequently, similar to *Adam2*, multiple candidate *Zp2* topologies limited the gene's global and ordinal phylogenetic utility.

Parsimony analysis of the *Prm1* alignment yielded 100 candidate trees, each about equally likely, and two candidate supertree-constrained topologies, each also about equally likely, none of which differed in global topology (all $P>0.06$, both tests; S3-S4 Tables). None of the three Orders represented by more than four species yielded single best ordinal topologies. Among the 12 Primates topologies one differed from the best tree ($P = 0.008$), and the best tree differed from its corresponding supertree-constrained topology ($P = 0.01$). In contrast, none of nine equally likely Cetartiodactyla topologies differed from a single best supertree-constrained topology, and none of six equally likely Rodentia topologies differed from either of two supertree-constrained topologies (all $P>0.14$). Overall, the *Prm1* tree yielded poor phylogenetic utility because of its many, similarly likely candidate topologies, discrepant groupings of taxa, and large proportion of unsupported nodes.

Parsimony analysis yielded four candidate *Tecta* trees, each about equally likely, and a single candidate supertree topology, with the global topology of the best *Tecta* tree differing (P <0.0001, AU test) from the supertree (S3-S4 Tables). Among single best ordinal *Tecta* topologies for Carnivora, Cetartiodactyla, Chiroptera, and Rodentia, only the Carnivora topology differed ($P = 0.04$, AU test) from its corresponding supertree-constrained topology. Similarly, among two topologies for Superorder Afrotheria, the best topology differed ($P = 0.01$, AU test) from its corresponding supertree-constrained topology. In Primates, two candidate trees did not differ ($P < 0.13$, both tests) from a single supertree-constrained topology; surprisingly, among the three trees the supertree-constrained topology was the more likely, highlighting a generally poor utility of *Tecta* for resolving relationships among primate species.

Finally, parsimony analysis yielded four candidate *Cytb* trees, each about equally likely, and a single candidate supertree-constrained topology. In contrast to the other five genes, which each yielded at least one candidate (unconstrained) topology that was more likely than the corresponding supertree-constrained topology, among the five *Cytb* trees the constrained tree yielded the most likely global topology, which did not differ (P>0.017, Bonferroni corrected, both tests) from the four candidate trees (S3-S4 Tables). Furthermore, among single best *Cytb* topologies for the Superorder Afrotheria and the Orders Cetartiodactyla, Chiroptera, Primates, and Rodentia as well as two ordinal *Cytb* topologies for Order Carnivora, only the Primates topology differed from the supertree (P<0.0001, AU test). Given its generally poor resolution (>38% of nodes unsupported), profoundly discrepant groupings of taxa, and multiple candidate tree topologies that were less likely than the supertree-constrained topology, the *Cytb* tree also yielded much poorer phylogenetic utility than *Zan*.

Altogether, among the six genes examined, only *Zan* yielded a phylogenetic tree that was more highly resolved than and congruent with accepted relationships portrayed in a well-established Eutherian supertree.

<u>Data Availability</u>

The species and accession numbers, nucleotide and protein alignments, and resultant trees are available in the DRYAD Digital Repository: Roberts, Emma K., Tardif, Steve, Wright, Emily A., Platt II, Roy N., Bradley, Robert D., Hardy, Daniel M. Rapid divergence of a gamete recognition promoted macroevolution of Eutheria. Datasets. DRYAD.doi 10.5061 (2022), accessible at URL https://datadryad.org/stash/dataset/doi:10.5061/dryad.44j0zpcdf.
