# Supplementary material for: Rapid divergence of a gamete recognition gene promoted macroevolution of Eutheria
Source: Genome Biol. 2022 Jul 11;23:155. doi: 10.1186/s13059-022-02721-y (PMC9275260; doi:10.1186/s13059-022-02721-y)
Supplement: Supplementary file 4 — Additional file 4. Comprises the peer review history. [file 13059_2022_2721_MOESM4_ESM.docx]

**Review history**

**First round of review**

**Reviewer 1**

The case laid about by the authors would be very interesting if true. I don't think there is any disagreement here. I also see no contention about several findings (e.g. Zan is under positive selection). The case made on lines 436-453 make a strong case for species specificity (why is this material not in the introduction?) However, where the core issues reside are over whether the authors have done enough to demonstrate their case.

Here I have a big issue: the authors are not clear what they wish to conclude. The title seems unambiguous:
"Rapid divergence of a gamete recognition gene promoted macroevolution of Eutheria"
That could have read:
"Rapid divergence of a gamete recognition gene may have promoted macroevolution of Eutheria".
or
"Zan is a candidate "speciation gene" in eutheria".
But no, the authors went for a title that makes a strong claim and it is this that the reviewers are questioning - have they shown this? Actually, I'm not sure the authors know the answer. We need only go to the end of the abstract to find:
"We therefore propose that species-specific egg recognition conferred by Zan's functional divergence served as a mode of prezygotic reproductive isolation that promoted the extraordinary adaptive radiation and success of Eutheria".
Why did they not write that "We therefore conclude that…"
In the conclusion we have a third take "Zan appears to be a speciation gene …". But then again on page 21 we get: "we conclude it is a speciation gene in placental mammals". On p 22 we get "Zan is only the second speciation gene identified in mammals". On line 21 in the abstract we have another firm claim " Here we identify Zan as a speciation gene".
So we have a deep problem here - the authors don't know the strength of what they want to conclude. We have everything from "appears to be" to absolutely is. I'll return to this issue in my final recommendations at the end.
I have other problems with the language and conclusions. In the abstract they say that Zan "promoted" the adaptive radiation. But I don't see strong evidence for this. Indeed, they note cases where Zan is little diverged and hybrids are made. But if sometimes these hybrids are sterile (Haldane's rule etc), then it is the hybrid sterility gene that promotes the speciation as it is the first to show up. Indeed, I could indeed imagine a model in which hybrid sterility evolves first and Zan variants are then selected for as they prevent fertilization thus permitting reproductive compensation (the mother just mates again, so a Zan variant is beneficial saving the mother all the resources of raising a sterile progeny (see also Charlesworth, B. (1994). "The evolution of lethals in the t-haplotype system of the mouse." Proceedings of the Royal Society of London Series B-Biological Sciences 258: 101-107 for how selection can favour earlier reproductive failure when the progeny will be sterile.). Would this be compatible with "promoted"? It is also possible that Zan just keeps ticking away and way after speciation has happened prevents sperm egg fusion as an incidental by product. Perhaps receptor and Zan continuously co-evolving within a species and after some time the divergence is great enough to cause a species-species pre-zygotic (just) isolating mechanism? If so, why is Zan and the receptor co-evolving? In this model there is no sense of "promoted". I can't however see any robust evidence that this model is discounted. Unless this model can be ruled out then the strong claim on line 421 is premature (the claim that "extraordinary congruence between Zan evolution and species diversification reflects a function of zonadhesin in speciation."). It might just sit in a sweet spot of rates of evolution that give it excellent resolution (the worry of many referees).

So I am confused as to what the authors wish to claim and the exact speciation model they are arguing for when claiming this actually promotes speciation.

The next question is what is the strength of the evidence. My summary would be that this is intriguing but far from solid.
A core issue is the strength of the evidence that this gene's phylogeny is concordant with the canonical mammalian phylogeny. Sensibly reviewers pointed out that we need evidence that Zan is somehow exceptional. There is then a lot of noise about whether fast evolving genes should well describe phylogenies. The authors claim that fast evolving genes are left out and (line 426) that as it is under positive selection it should create a rubbish phylogeny. Neither are accurate and it depends on how recent the events are to be reconstructed: if recent then fast evolution DNA sequence (not slow) are needed, if ancient then indeed we usually assume the fast ones lose signal, as do synonymous sites, so restrict analysis to protein alignments of slow to moderates - depending on age to be reconstructed.

In this regard the most striking result would be concordance between a fast gene and really old nodes. The authors claim this but I'd like better evidence here. We need to show better concordance as a function of node age (I expect good concordance with recent nodes - perhaps this explains the overall high concordance?).

Second, why (line 223) did the group use parsimony (for Fig 2 they use Bayes)? Parsimony is known to be really misleading especially in the face of mammalian mutation biases (eg high CpG->TpG) and much better methods are available. I'm not sure what the meaning of the number of equally parsimonious trees actually is in this context. The Bayesian output should give a series of approximately equally likely trees - is this space more constrained?

Third, the plot I'd like to see is, on the x -axis some measure of mean rate (PAML's estimate of omega assuming one rate may do) and on the Y axis some measure of agreement (the authors use % nodes not in agreement). One either does this in 3 dimensions with alignment size as the Z axis or control for alignment size. If on such a plot Zan sits as a lovely outlier given size and omega then your case is looking much stronger. One referee suggested using all positively selected genes previously identified - so to answer the authors question as to how many are enough the answer is clear - all. If it was me I'd do all genes regardless of omega that I could align over the same distance. I wasn't convinced by the few hand-picked examples although I understand the motivation of the authors. But why not do phylogeny properly (ML or Bayesian) and use a properly large sample of genes and really see where Zan sits in omega-node prediction space? This, after all is the key evidence in the paper.

Overall then I see confusions about the strength of claims and many holes in the analysis to support the (common) strong claims. I see three ways to go:
1. Reject on the basis that the strong claims are not supported - the same as MBE's decisions (which I agree with).
2. Do the analysis really properly as requested by referees (a full omega %nodes plot) or
3. Shrink this to a brief communication with the tone much qualified.
I note that the last option is viable as many of the results (part 1) can easily go to a supplement. The discussion is also way too long. You start with Zan as a good candidate given prior data (fertilization evidence, prior evidence of high omega) then briefly detail the core results. The paper should then be titled "Is Zan a Eutherian speciation gene?" or "Zan is a candidate "speciation gene" in eutheria". Tone down all the claims to have proven anything, suggest the key next tests (the ones you argue yourselves out of), clarify models and alternative explanations for the data (as above).
I would be happy with any of these but I can easily see why the authors might well prefer option 3.
I hope my comments prove helpful.

**Authors’ response to reviewers**

The reviewer requested a major new experiment (support vs selection for all positively selected genes), which we have done. We believe the results address the reviewer's concern, and thank her/him for the suggestion, as the new data have greatly strengthened the paper. The reviewer also requested revisions to the manuscript, mostly shortening, which we have also done, both by removing extraneous text

(residual bloat from revisions we were asked to do in response to review at another journal), and by moving some Results text to the SI. Finally, we revised the text for consistency of our conclusions, also as the reviewer requested.

**Second round of review**

**Reviewer 1**

The authors have done the large analysis suggested. Fig 8 is a major addition to the paper and clearly shows that Zan is indeed an outlier. The debate as to whether fast evolving genes necessarily recover the deeper nodes is now well resolved by the authors. I see no reason not to recommend acceptance.
